# Evaluation of the clinical effectiveness of a work-based mentoring programme to develop clinical reasoning on patient outcome: A stepped wedge cluster randomised controlled trial

**DOI:** 10.1371/journal.pone.0220110

**Published:** 2019-07-31

**Authors:** Aled Williams, Alison Rushton, James J. Lewis, Ceri Phillips

**Affiliations:** 1 University Hospital of Wales Physiotherapy Department, Cardiff and Vale University Health Board, Cardiff, Wales, United Kingdom; 2 School of Sport, Exercise and Rehabilitation Sciences, University of Birmingham, Birmingham, England, United Kingdom; 3 Y Lab (Public Services Innovation Lab for Wales), School of Social Sciences, Cardiff University, Cardiff, Wales, United Kingdom; 4 College of Human and Health Sciences, Swansea University, Swansea, Wales, United Kingdom; Universite de Bretagne Occidentale, FRANCE

## Abstract

**Background:**

Despite persistent calls to measure the effectiveness of educational interventions on patient outcomes, few studies have been conducted. Within musculoskeletal physiotherapy, the effects of clinical mentoring on postgraduate physiotherapists have been explored, but its impact on patient outcomes is unknown. The objective of this trial was to assess the effectiveness of a work-based mentoring programme to facilitate physiotherapist clinical reasoning on patient outcomes.

**Methods:**

In a stepped-wedge cluster RCT in the musculoskeletal physiotherapy outpatient departments of a large NHS organisation, 16 physiotherapists were randomised by cluster to receive the intervention—150 hours of mentored clinical practice—at one of 3 time periods; control was usual training. 441 patients submitted outcome measures: Patient-Specific Functional Scale (PSFS) (primary outcome measure), EQ-5D-5L, patient activation and patient satisfaction (secondary outcome measures). A further secondary outcome measure of physiotherapist performance was collected by an independent assessor observing the physiotherapists practice.

**Results:**

80.0% of intervention patients achieved clinically significant PSFS scores compared with 63.8% of control patients. Binary logistic regression analysis modelling for time, cluster and patient characteristics showed strong statistical evidence for this difference (p = 0.023; odds ratio 4.24, 95%CI 1.22, 14.79). Physiotherapist performance scores improved from a mean of 47.8% (SD 3.60) pre-intervention to a mean of 56.0% (SD 4.24) (p<0.001). There was no statistical evidence for differences between groups on other secondary outcomes.

**Conclusion:**

This is the first study that we aware of that provides patient outcomes measurement of an established educational intervention in physiotherapy, providing evidence that this type of intervention positively impacts patient outcomes and physiotherapist performance. This provides a basis for further research in education across other healthcare disciplines and outcome measures.

## Introduction

Minimal research has investigated the effect of healthcare education and training on quality of care and patient outcome. The paucity of evidence has led the UK Department of Health to call for research investigating healthcare education and patient outcomes [1], specifically measuring staff and patient experience, clinical effectiveness and safety [2]. This call to measure the association between healthcare intervention and patient outcome is not new in the medical education literature, where it has long been argued that the education and development of clinicians should be evaluated on the basis of whether they achieve better health outcomes [3–8]. Different levels of evidence have been outlined, advocated and widely used for healthcare education, as illustrated by Kirkpatrick’s 4-level method for training evaluation [9] and Moore’s 7-level [10] outcomes model of continuing medical education. Table 1 below illustrates the longer established Kirkpatrick’s model along with examples of typical research measurements:

**Table 1:** Kirkpatrick’s 4 levels of training evaluation [11].

Levels and Descriptors [11]		Examples [12]
**Level 1: Reaction**	A measure of the satisfaction of the participants who attended the program	Survey
**Level 2: Learning**	The extent to which participants increased their knowledge, learned or improved present skills, or changed their attitudes	Test/examination
**Level 3: Behaviour**	The extent to which participants applied what they learned when they returned to their jobs	Ratings by supervisor, peers, patients; direct observation
**Level 4: Results**	The improvement of morale, the increase in sales or production, the reduction in turnover, the increase in customer satisfaction, the return-on-investment, and any other benefits that came from attending the program.	Validated patient outcome scales

It has been generally acknowledged that “Level 4 evidence” of patient outcomes on Kirkpatrick’s scale is difficult to obtain [13–17] commonly leading to lower level “surrogate outcomes” such as competency and clinician performance being used to evaluate educational interventions [3, 7]. Where level 4 outcomes have been obtained in medicine [18, 19] and nursing [20–22] the most frequently used outcomes to evaluate training and education are mortality rates, length of time in theatre, length of stay in hospital, complication rates and patient satisfaction. While this represents progress within the published research by exploring experience and safety (adverse events and patient mortality rates], measuring the clinical effectiveness of clinician education on patient outcomes continues to lack published evidence. Multiple authors across specialties in healthcare continue to call for the “gold standard” of high-level patient outcomes in the recent literature [12, 15, 16, 23–27].

This is illustrated within the field of musculoskeletal physiotherapy, where there are only two published studies to the best of our knowledge which have explored the clinical effectiveness of an educational intervention for physiotherapists on their patient outcomes. The first [28, 29] showed no effect on patient outcomes of an 8-day psychosocial course when compared with the outcomes of a waiting list control group of clinicians. The fact that the educational intervention was delivered away from the clinical context of the clinicians was a weakness of this study and conceded by the authors to be a likely factor in the lack of effect on patient outcomes. The second [30] demonstrated a positive effect on patient outcomes by utilising an educational approach within the usual clinical context of the clinicians for patients with neck pain and disability. However, this trial has a narrow clinical focus on the cervical spine, as well as some methodological issues (retrospective data collection, limited confounding variables factored into analysis, individuals in different arms of the trial working in the same clinic introducing the risk of contamination) which caveat these positive findings. Apart from studies using patient outcome to measure effectiveness, multiple studies have explored education for advanced practice at postgraduate level, where mentoring in the clinical environment is used nationally and internationally to develop clinical reasoning and other features of expert clinical practice [31–36]. Qualitative studies investigating the clinical mentoring component of such postgraduate programmes have demonstrated positive effects on physiotherapist performance and career, and the mentored practice component of postgraduate programmes is consistently evaluated as an effective component of Masters level education on transforming practice [31–39]. While the effects of this educational intervention have been explored on the physiotherapist, to the best of our knowledge, its clinical effectiveness on patient outcomes has not been investigated to date.

In summary, there is a gap in the current evidence of the effectiveness of educational interventions as measured by patient outcome; the objective of this trial was to evaluate the clinical effectiveness of a work-based mentoring programme that aims to develop clinical reasoning for physiotherapists, on patient outcome. This objective was achieved as follows.

## Methods

### Design

**Fig 1:**
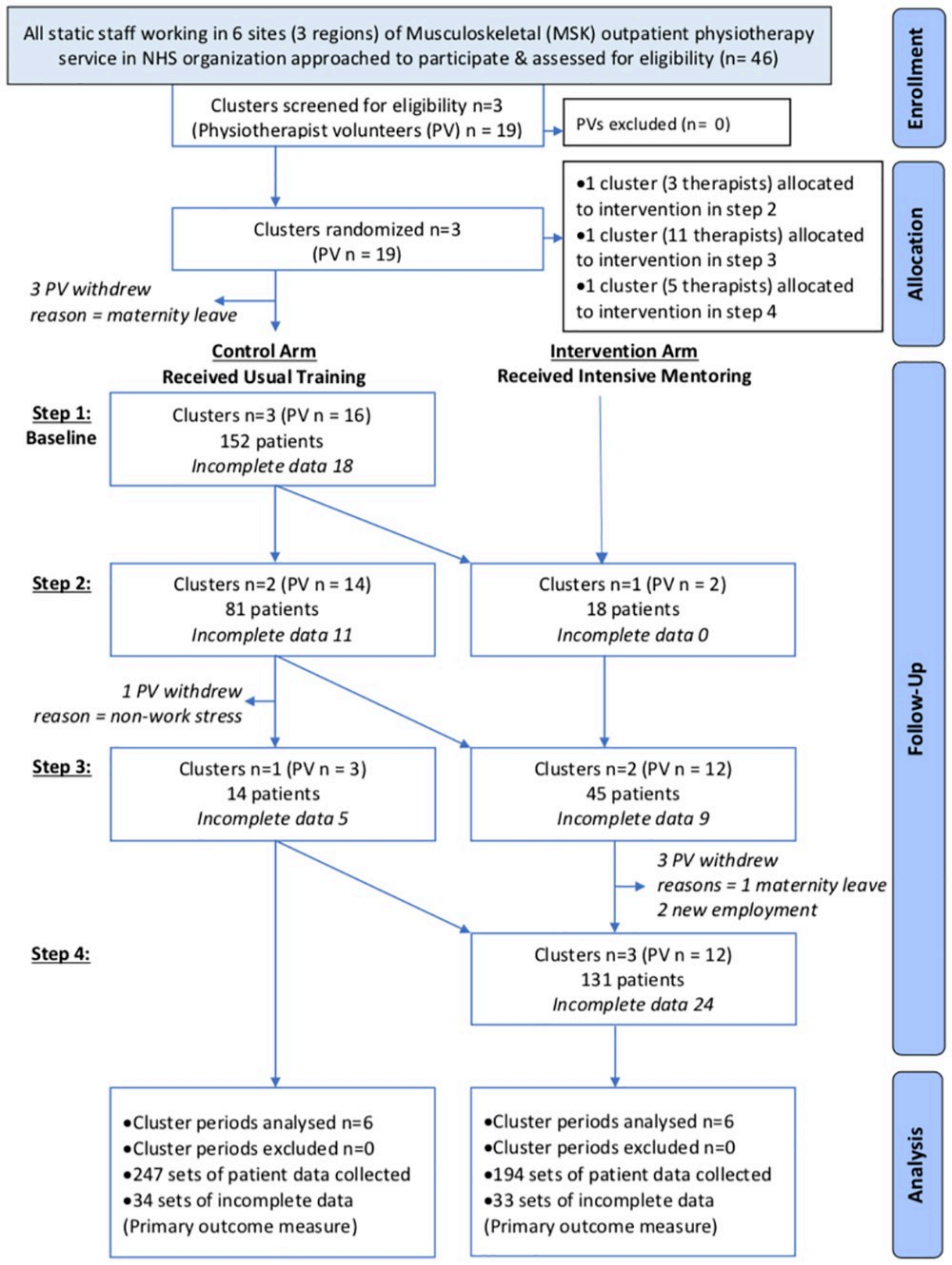
Modified CONSORT 2010 flow diagram.

A stepped wedge cluster randomised controlled trial (SWT) was designed and registration was assigned on 31/07/2012 (ISRCTN79599220); there are no ongoing or related trials for this intervention and therefore none are registered. The protocol was published [40] in line with the SPIRIT 2013 statement [41]. There were no deviations from the protocol. Clusters were defined as groups of physiotherapists (departments) within the outpatient musculoskeletal physiotherapy service of a large NHS organisation. This organisation had 6 sites for service delivery each based in a separate hospital with specific staffing localised to each site. The 6 sites were organised into pairs for training purposes, and so to reduce the risk of any potential contamination across sites, those pairs formed the 3 clusters (North, South and West) which was the unit of randomisation. Fig 1 illustrates the flow of participants through the trial (using the CONSORT 2010 extension [42] incorporating a flow diagram structure used in reporting of recent SWTs [43, 44]) as well as the patient outcome data collected at each point in each arm of the trial.

The rationale for a clustered design was the nature of the intervention and outcome. The training of clinicians was a cluster-level intervention as such education programmes are frequently targeted at departments or practices, and it is assumed that physiotherapists within a department might share their experiences [45–47]. The effect of the intervention on patient outcome also required measurement of clusters of patients treated by the physiotherapists before and after the intervention.

In the SWT design, the intervention is rolled out to all clusters over time, with the order in which they receive the intervention being randomised [48]. This design was selected primarily for logistical and pragmatic reasons as this research was interested in the effects of this intervention being rolled out using outcomes for which it has not previously been considered, and the logistical and practical infeasibility of delivering this intervention (which is time and specialist ‘heavy’) in a parallel or crossover design [49–53].

**Fig 2:**
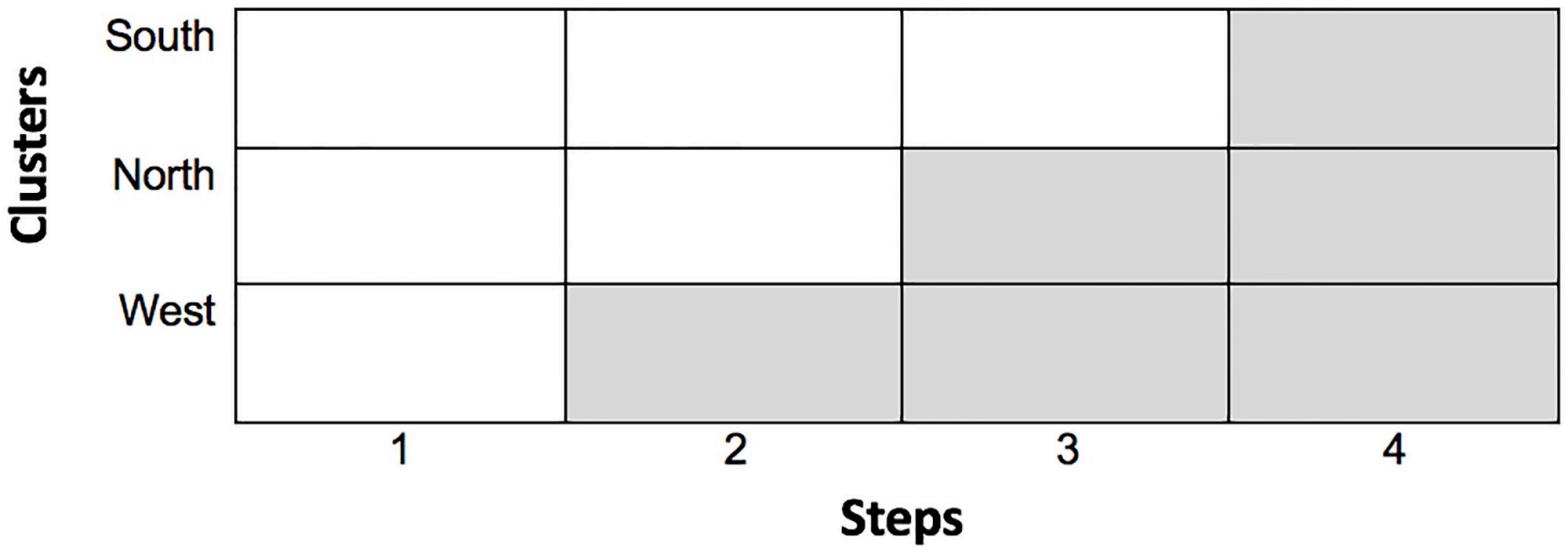
The stepped-wedge design for the trial.

Intervention: The intervention was rolled out as illustrated in Fig 2 below, with white cells representing control periods, grey cells representing intervention periods; each cell also represents a data collection point.

The first step (time point) corresponds to the baseline measurement where none of the clusters had received the intervention, which is usual practice for this design [53]. At each subsequent step a cluster of participating physiotherapists crossed over from control to receive the intervention. The intervention was delivered at the start of the 6-month time-period, to allow for consolidation and application of the programme, before data collection occurred at the end of the time-period.

The intervention was a 150-hour clinical mentorship programme aimed at facilitating clinical reasoning. The rationale for the intervention was drawn from the educational standards document of the International Federation of Orthopaedic Manipulative Physical Therapists (IFOMPT) [54], a non-governmental federation promoting international excellence and unity in clinical and academic standards in the field of musculoskeletal physiotherapy; it is a subgroup of the World Confederation for Physical Therapy, which is a recognised partner of the World Health Organization. A minimum of 150 hours of mentored clinical practice is recommended for students, where the clinical mentor is a member of the member organization of IFOMPT. Furthermore, the clinical mentoring component of masters programmes that has been explored in terms of its impact on physiotherapist performance and career (as highlighted in the introduction) utilises this same educational approach. The intervention was delivered by mentors who were members of the Musculoskeletal Association of Chartered Physiotherapists (MACP), the UK member organisation of IFOMPT, having qualified at MSc / PgD level from a higher education establishment and who also had previous experience in delivering such mentorship at post-graduate level. The intervention took place in the usual clinical context of the participating physiotherapists, consisting of the mentors observing the participating physiotherapists assessing and treating new and follow-up patients, discussing and facilitating clinical reasoning processes immediately after the patients left the clinic. The model of clinical reasoning utilised for discussion was the dialectical model of Edwards et al [55] derived from qualitative studies of expert physiotherapists incorporating diagnostic and management aspects of clinical reasoning (such as narrative, collaborative and hypothetico-deductive reasoning) [56–58].

Control: During the control steps of the trial, participants received their usual training allocation which involved in-service training on current evidence applied to practice (4 hours per month), technique sessions on practical skills (30 minutes per week), as well as mentoring sessions observing clinical reasoning (1.5 hours per month). This choice of control was on the basis that the comparison group needed to be plausible and fair [59] and that comparisons with non- intervention controls fail to inform on the issue of selecting the most effective methods from multiple available options [60].

### Participants

All qualified physiotherapists working in the selected NHS organisation whose majority of time practising was in the musculoskeletal outpatient context were approached to participate. Consecutive consenting patients attending the outpatient musculoskeletal physiotherapy service for treatment by the participating physiotherapists during data collection periods who were eligible to participate were also approached. Exclusion criteria are outlined in Table 2 below:

**Table 2:** Exclusion criteria.

Criteria	Reason:
***Physiotherapist***	
Majority of working practice outside of MSK outpatient context	Expertise accepted as context-dependent [55, 61–64]
Already undertaken postgraduate placement	Already exposed to similar intervention
Rotational staff	Not present in service for duration of trial
***Patient***	
<18 years of age	Outcome measures validated for adults and English language [65–72]
Not English-literate	

#### Ethical considerations

**Fig 3:**
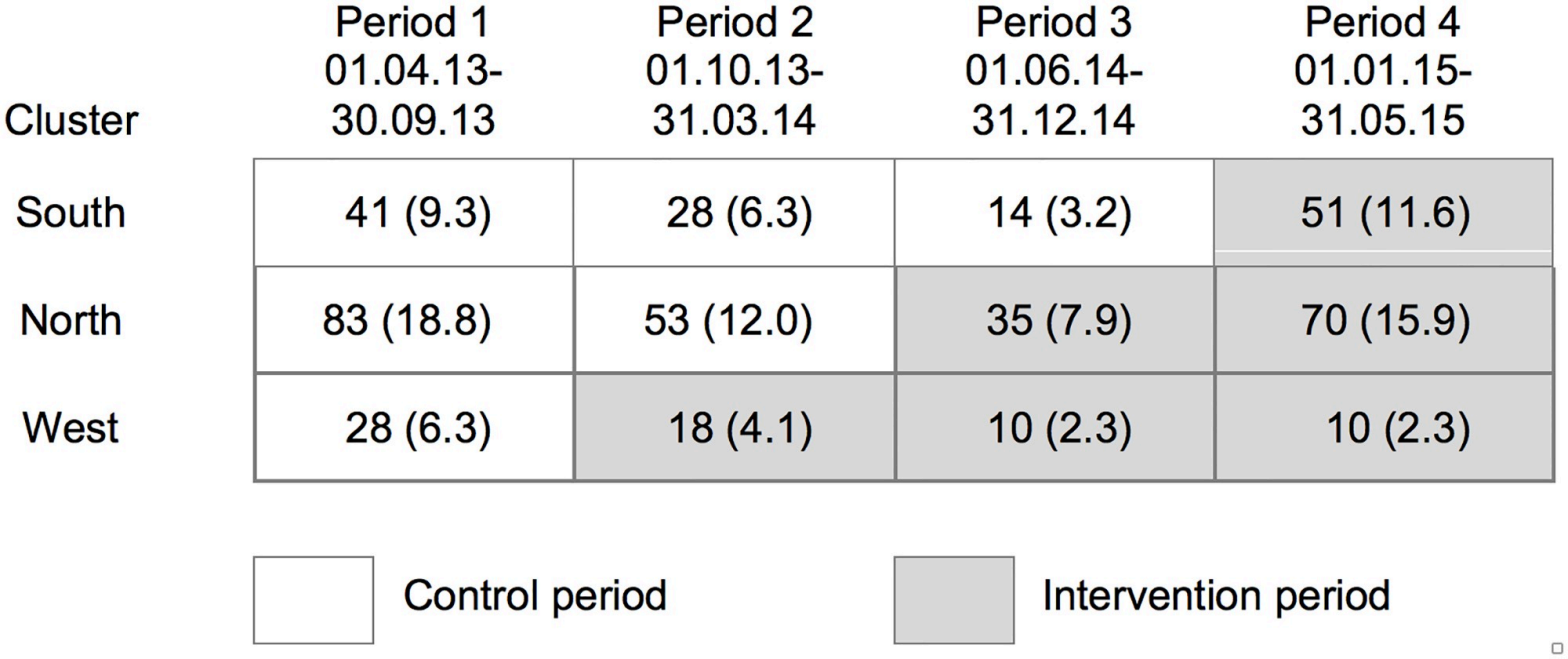
Patients recruited, and data collected in each cluster period n (%).

Ethical approval for the trial was sought and obtained from the regional Research Ethics Committee (ref: 12/WA/0078) on 20/04/2012 and informed written consent was gained from the participating physiotherapists and their patients. The physiotherapist recruitment period was from 01/09/2012 to 28/02/2013 and patient recruitment was from 01/04/2013 to 31/05/2014 (as seen in Fig 3). Follow up was at discharge with a cut-off at 12 months post-recruitment; no patients exceeded this cut-off. Participation in the trial was on a voluntary basis. Physiotherapists were reassured that refusal to consent to be part of this trial would not influence their receipt of training or have any other impact on their professional development and participating patients were assured that refusal to consent to complete outcome data for this trial would have no impact on their care.

### Outcome measures

#### Patient level outcomes

The primary outcome measure was the proportion of patients achieving clinically significant improvements on the Patient Specific Functional Scale (PSFS), a patient-specific tool for measuring health-related quality of life (HRQL). The tool asks that patients select their most relevant activities or functions and then rate their ability to complete this activity on a 11-point scale at a level experienced prior to injury or change in functional status, where "0" represents “unable to perform” and "10" represents “able to perform at prior level”. Patients were asked to select a value that best described their current level of ability on each activity. These outcomes were collected in collaboration with the patient’s physiotherapist (which is the advised method for use of the tool [73]) and entered on to the patient’s questionnaire containing the other outcome measures. Clinical significance was set at a level using established data for the minimal clinically important difference (MCID) being reported to be 3 points for each single identified activity and 2 points for aggregate activities. The proportion of patients achieving clinically significant outcomes (i.e. achieving the level for MCID) was calculated.

The secondary outcome measures at patient-level were EQ-5D-5L, the Patient Activation Measure and the MedRisk instrument. The EQ-5D-5L is a two-section generic measure of HRQL [74], the descriptive section yielding a 5 digit health state profile which was then converted into a single index value and the visual analogue scale giving respondent’s self-rated health on a scale of 0–100; higher scores in both sections indicate higher levels indices of health. The Patient Activation Measure (PAM) is a measurement of the knowledge, skills and confidence a person has in managing their own health and healthcare [68], delivering a patient activation score of between 0 and 100, higher scores indicating greater levels of activation. The MedRisk instrument for Measuring Patient Satisfaction with Physical Therapy Care (MRPS) is a measure of patient satisfaction in the musculoskeletal physiotherapy context [72], mean scores being used to assess satisfaction from 1.0 (highly dissatisfied) to 5.0 (highly satisfied). These secondary measures all have established measurement properties [67–69, 74–80]. The patients completed self-assessment questionnaires which contained a unique identifier for the treating physiotherapist on each questionnaire to ensure blinding and sent to the lead researcher. Patient outcomes were completed at baseline (after the first appointment with the physiotherapist) and endpoint (discharge); timescales between these points varied between patients depending on the length of the course of treatment from 3 weeks to 13 weeks. No patients crossed over from control to intervention periods.

#### Physiotherapist level outcomes

Physiotherapist performance was assessed by each physiotherapist being observed with a new patient and a follow-up patient and then discussing their practice. This was performed by an assessor who was blinded (to whether the participant had received the intervention), independent (from outside the organisation) and experienced (familiar with using the performance criteria). Two physiotherapist-level outcomes were used; both derived from the assessment of Master’s level post-graduate student performance in the musculoskeletal context [32]. One outcome was the score and the other outcome was the score divided into 10% bands (e.g. 40–49%; 50–59%). The specific tool selected for measurement of performance was selected on the basis of its supporting empirical data where the construct of the assessment tool was supported by triangulation of data from multiple methods and studies [31, 33, 81, 82].

### Sample size

Analysis was to be performed using 2 different estimates of intergroup differences; clinically, the more relevant use of the primary outcome measure was estimated to be 10% more patients achieving clinically significant outcomes on PSFS but as there was limited data on which to accurately base a sample size calculation, a second estimate was used for the power calculation. Sample size calculations followed the approach outlined for stepped-wedge designs in Hussey and Hughes [53] and used conventional values of 0.80 for statistical power and 0.05 for statistical significance, and were performed for a realistic sample of 12 participating physiotherapists organised into 3 clusters. A mean improvement of 1 point on the PSFS with a standard deviation of 2.0 (assuming a PSFS difference of 0.5 across the control arm and a 1.5 difference across the intervention arm) with an ICC of 0.05, a sample of 432 patients was required. An increase in the assumed ICC to 0.5 would result in a small drop in power to 77%.

### Randomisation and blinding

The unit of randomisation was the cluster, defined as physiotherapy departments. The participating physiotherapists and clinical mentors implementing the intervention were aware of which cluster was receiving the intervention [49, 52]. The clusters of participating physiotherapists were randomly allocated to the sequence of intervention (to receive the intervention in time period 2, 3 or 4), by computer programme [83] by one of the clinical mentors. To ensure allocation concealment and the blinding of the lead researcher to the sequence generation and intervention allocation, this mentor also allocated a unique identification code to each physiotherapist that was used on all outcome measure questionnaires and kept a sealed copy of the key code linking codes to physiotherapists. This mentor was involved in delivering mentoring in all 3 clusters and was not involved in any data analysis. The key code was only opened by the lead researcher once all outcome data had been collected and analysed from each of the 4 time points. The independent assessor of physiotherapist performance was blinded as to whether the physiotherapist has received the intervention during their assessment. Patients were also blinded to whether their treating physiotherapist had received the intervention. In summary, patient outcome assessment was standardized but not blinded to the data collector, physiotherapist outcome assessment was blinded to the data collector and data analysis was blinded.

### Statistical methods

The characteristics of the patient populations exposed and unexposed to the intervention in the trial are summarised using frequencies and percentages, means and standard deviations, as appropriate. The null hypothesis, that there was no difference in the proportion of eligible patients achieving clinically significant outcomes before and after their physiotherapists had exposure to the intervention, was tested using a logistic regression model with a binary outcome—whether or not each patient achieved the MCID on the PSFS.

As this was a SWT, it was essential to adjust outcomes for cluster [84] and for step (time) [85]. This was achieved by fitting simple logistic/linear regression with linear trend for step and fixed effect for cluster. The analysis took account of the within cluster correlation by adjusting for each cluster with a fixed effect. This is not possible in a parallel cluster randomised trial, due to the multicollinearity between cluster and trial arm, which would over-parameterise the model. However, because this is a stepped wedge trial, where each cluster is in both the control arm and then the intervention arm, this is a valid approach. A recent article has highlighted different extensions where time can be fitted to the model as a random or linear variable [84]. In our trial with only 3 clusters it was not possible to estimate the variance parameter of a random effect. In addition, estimating cluster, step and arm all as fixed effects would over-parameterise the model; as a compromise step was fitted as a linear trend. This adjustment for cluster and step is reported as the minimally adjusted model. In addition, a second model additionally adjusted independent variables that were deemed important to consider including: patients’ duration of symptoms (chronicity being a likely indicator of poor outcome [86]); exercise history (previously shown to be an indicator of outcome [87]); OrebroMPQ (predictive of poor outcome [88, 89]); initial PAM (patients’ ability to self-manage being likely to influence outcome [69, 90, 91]); and other patient factors (initial PSFS, EQ5D index, sex, age, ethnicity) [87]. This is referred to as the fully-adjusted model. Significance was set at the 5% level and 95% confidence intervals were reported. Null hypotheses and analyses for secondary outcomes took a similar form to that for the primary outcome. Two sensitivity analyses were also performed to address missing data for best (all missing data achieving MCID) and worst case (all missing data not achieving MCID) scenarios. All analysis was by intention to treat.

## Results

The baseline characteristics of the patients recruited to the trial are outlined in Table 3. The control arm contained more female patients, and more patients who had less of a background in exercise. The intervention arm contained more patients who the Örebro questionnaire classifies as high risk i.e. at higher likelihood of a poor outcome. It was important, therefore, to model for these factors in the final analysis. Other between group differences appear small.

**Table 3:** Baseline characteristics of exposed and unexposed patients (N = 441).

Variable		Control Arm N (%)	Intervention Arm N (%)
Gender	Male	74 (30.0)	78 (40.2)
	Female	173 (70.0)	115 (59.3)
	*Missing*	*0*	*1 (0*.*5)*
Ethnicity	White British	220 (89.1)	170 (87.6)
	Other	24 (9.7)	24(12.4)
	*Missing*	*3 (1*.*2)*	*0*
Örebro (ÖMPQ) Risk	Low	161 (65.2)	125 (64.4)
	Moderate	55 (22.3)	35 (18.2)
	High	23 (9.3)	32 (16.7)
	*Missing*	*8 (3*.*2)*	*2 (1*.*0)*
	Mean (SD)	93.1 (28.75)	96.1 (33.35)
Activation (PAM) Level	1	31 (12.6)	21 (10.8)
	2	45 (18.2)	33 (17.0)
	3	77 (31.2)	64 (33.0)
	4	93 (37.7)	76 (39.2)
	*Missing*	*1 (0*.*4)*	*0*
	Mean (SD)	62.1 (15.57)	63.9 (14.45)
Exercise History	Seldom/Never	64 (29.0)	42 (21.6)
	X1/2 per week	72 (29.1)	58 (29.9)
	At least X3/week	85 (34.4)	81 (44.8)
	*Missing*	*26 (10*.*5)*	*13 (6*.*7)*
		**Mean (SD)**	**Mean (SD)**
Age (years)		51.8 (15.26)	49.4 (15.65)
	*Missing*	*1*	*1*
Pain duration (months)		50.79 (90.52)	47.19 (78.12)
	*Missing*	*14*	*6*

### Recruitment and follow-up to the trial

The stepped wedge CRT design was carried out as represented in Fig 3. 441 patients were recruited by 16 physiotherapists (power calculations required 432 patients). The clusters were different sizes–North being the biggest and West the smallest.

### Primary outcome measure

**Fig 4:**
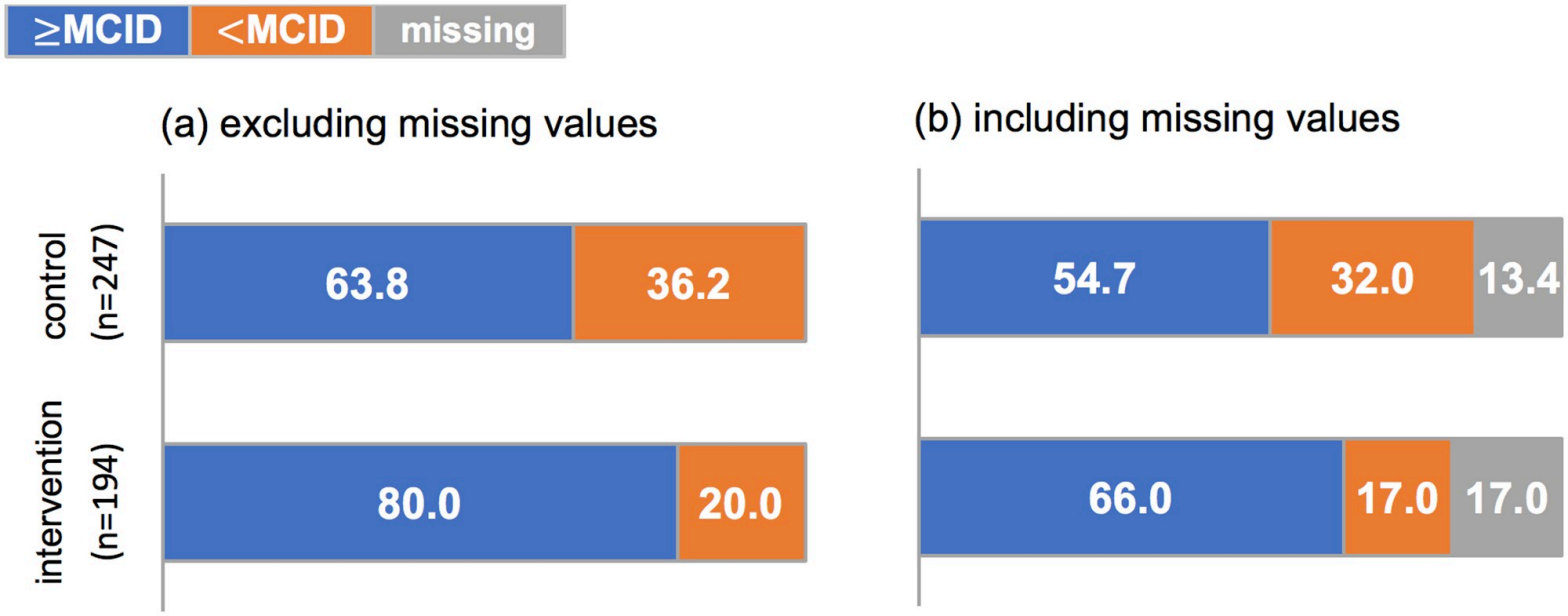
Distribution of clinically significant Improvements in PSFS by trial arm excluding (a) and including (b) missing values.

Using the established data for the minimal clinically important difference (MCID) of 3 points for each single identified activity and 2 points for aggregate activities, the proportion of patients achieving the level for MDC and MCID was 80.0% of the patients in the intervention arm compared with 63.8% of patients in the control arm (Fig 4).

Using logistic regression analysis, in the minimally adjusted model the difference in the proportion of patients achieving the MCID between the intervention and control arm gives an odds ratio of 4.14 and a p value of 0.009. In the fully adjusted model, the odds ratio strengthens to 4.24 with a p value of 0.023 (Table 4).

**Table 4:** Summary of primary outcome measure with adjusted and unadjusted intervention effects.

	Control Arm N (%)	Intervention Arm N (%)	Logistic Regression Model					
			Minimally adjusted^1^			Fully adjusted^2^		
Patients	247	194						
PSFS change			P value	Odds ratio	CI (95%)	P value	Odds ratio	CI (95%)
MCID achieved	135/212 (54.7)	128/161 (66)	.009	4.14	1.42 to 12.03	.023	4.24	1.22 to 14.79
*Missing*	*35 (13*.*4)*	*33 (17*.*0)*						

^1^ adjusted for cluster and time;

^2^ adjusted for sex, age, duration of symptoms, exercise history, OrebroMPQ, initial PSFS, initial PAM, initial EQ5D index

#### Sensitivity analyses

To explore the potential effect of missing data, two sensitivity analyses were performed, assuming all patients with missing outcome data to have made a good clinical outcome (for a best-case scenario), and then assuming all patients with missing outcome data to have made a poor clinical outcome (for a worst-case scenario). In the first sensitivity analysis, all missing outcomes were recoded as clinically significant. This adjustment still yielded a statistically significant value for the intervention in the minimally adjusted model (p = 0.018) with an odds ratio of 3.38 (95%CI 1.24, 9.27) as well as in the fully adjusted model (p = 0.020) with an odds ratio of 4.23 (95%CI 1.25, 14.31). In the second sensitivity analysis, recoding all missing outcomes as clinically non-significant, the effect of the intervention was again statistically significant in the minimally adjusted model (p = 0.012) with an odds ratio of 3.19 (95%CI 1.29, 7.89) but this statistical significance is lost in the fully adjusted model (p = 0.074, odds ratio 2.74 95%CI 0.91, 8.27).

A further analysis was performed by aggregating the PSFS data and comparing mean changes as a second view of the primary outcome, as pre-planned in the protocol. This was performed to explore whether similar effects were seen using scale data and linear regression modelling as were seen using the binary data with logistic regression. During the power calculation for the study, a difference of 1.0 points was used as the estimate for what could be perceived as clinically significant. The minimally adjusted model (using the same factors as used for the logistic regression modelling for the binary values) yielded a difference of +1.35 points for the intervention over control and is statistically significant (95%CI 0.14 to 2.55; p = 0.028). However, this loses statistical significance in the fully adjusted model to +0.42 points (95%CI -0.61 to 1.45; p = 0.42).

### Secondary outcome measures

#### Physiotherapist performance

**Fig 5:**
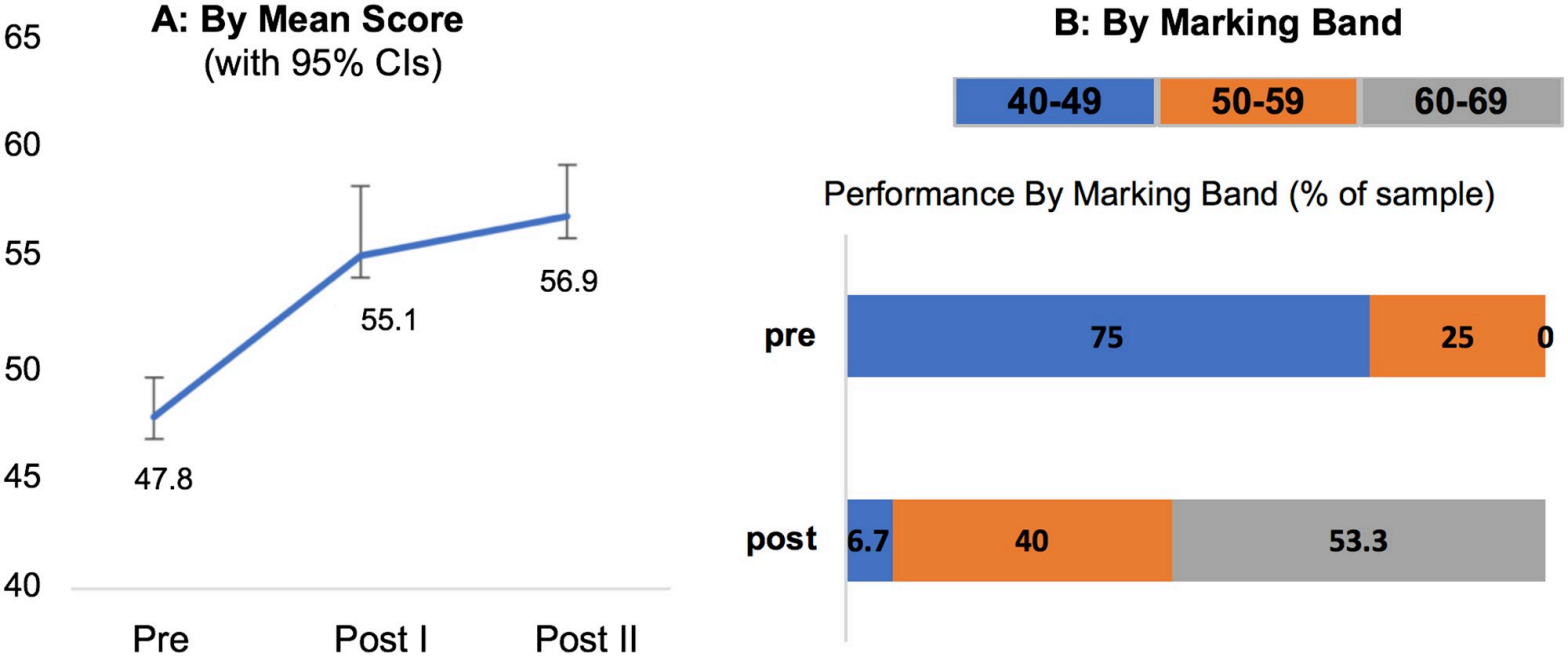
Changes in physiotherapist performance.

The physiotherapists’ performances improved as can be seen in Fig 5. Fig 5A shows the mean score of the physiotherapists’ performance as 47.8 prior to the intervention, and 55.1 post intervention (post I). A subsequent measure of performance post-intervention shows a further mean increase to 56.9 (post II). Fig 5B captures the clinical and educational relevance of these score changes. Displaying performance by banding, 75% of physiotherapists prior to intensive mentoring would have been below the mark of 50, which is the pass mark for Masters level performance on the University assessment criteria. This reduces to only 6.7% of physiotherapists post-intervention, with 53.3% of physiotherapists achieving a score in a higher band of between 60 and 70, which none achieved prior to the intervention.

#### Patient outcomes

The effect on patients measured using the primary outcome measure (selected amongst other reasons due to its responsiveness) was not reflected in the secondary outcome measures (Table 5). There was no statistical evidence for any differences between groups on generic HRQL (measured using EQ-5D-5L index and VAS), patient satisfaction (measured using the MRPS), or patient activation (measured with the PAM). It should be noted that there were more missing data in this section, with approximately a third of secondary outcome measures incomplete with little difference by trial arm. For the MRPS there were 77 incomplete outcomes in the unexposed arm (31.2%) and 71 in the exposed arm (36.6%); for the PAM and EQ5D there were 78 incomplete outcomes in the unexposed arm (31.2%) and 71 in the exposed arm (36.6%).

**Table 5:** Summary of secondary outcome measure by exposure to the intervention, with adjusted and unadjusted intervention effects.

			Linear Regression Model					
	Unexposed Arm N (%)	Exposed Arm N (%)	Minimally adjusted^1^			Fully adjusted^2^		
Patients	247	194	change	CI (95%)	P value	change	CI (95%)	P value
**EQ5D change**								
Index change	+0.097	+0.092	+0.033	-0.049 to +0.115	0.429	+0.023	-0.056 to +0.102	0.567
VAS change	+9.8	+9.6	-0.8	-8.65 to +7.04	0.840	-1.9	-9.50 to +5.71	0.624
*Missing*	*78 (31*.*6)*	*71 (36*.*6)*						
**Medrisk**								
Satisfaction score	4.6	4.6	+0.1	-0.17 to +0.27	0.649	+0.1	-0.17 to +0.28	0.645
*Missing*	*77 (31*.*2)*	*71 (36*.*6)*						
**PAM change**								
Activation score change	+8.4	+7.0	+3.5 (-6.04 to +12.98) p = 0.473			+2.8	-6.71 to +12.32	0.562
*Missing*	*78 (31*.*6)*	*71 (36*.*6)*						

^1^ adjusted for cluster and time;

^2^ adjusted for sex, age, ethnicity, duration of symptoms, exercise history, OrebroMPQ, initial PAM, initial EQ5D index

## Discussion

### Primary outcome

Facilitating physiotherapists’ clinical reasoning through work-based mentoring significantly increases the proportion of their patients achieving clinically significant patient-specific HRQL outcomes. While the statistical evidence demonstrated is important, the increase from just under 2 in 3 patients attending the physiotherapy service achieving the MCID on PSFS to 4 in 5 patients achieving this is a welcome increase for physiotherapists, referring clinicians, managers and not least of all, patients. This is especially important given that this outcome measure is set for the functional activities that patients themselves have identified as being most affected by their musculoskeletal condition and therefore the most relevant to address in the rehabilitation process. The greater effect of this educational intervention over that of usual training could be attributed to its nature: the facilitation of clinical reasoning, with a focus on narrative reasoning (exploring and understanding the patient’s lived experience), diagnostic reasoning (incorporating analytic and non-analytic reasoning), management reasoning (both instrumental and communicative strategies) as well as developing collaborative reasoning and consensual approaches to arriving at a diagnosis and selecting management goals and options are likely to foster a more patient-centred and efficient approach [55, 57]. It could also be attributed to its context; being conducted in the workplace allowed real time feedback specific to the clinician in the context of their usual work. A third factor could be its delivery being regular over several weeks, making reinforcement and practice change more likely [30].

### Secondary outcomes

The clinical effectiveness of the intervention on patient-specific outcomes is also reflected in improvements in physiotherapist performance, as seen in the blinded measures pre- and post-intervention. The finding of 75% of the participants being below the pass mark for a masters clinical module is unsurprising as the participants were without the input of a taught masters curriculum leading up to their assessment, making the improvements in performance in response to the intervention impressive in that over 90% of the participants would have ‘passed’ on a tool that measures performance at masters level. In addition, over half of the participants were assessed to be at a higher banding than the pass level. The validity of these findings are supported by the assessments being performed by a blinded assessor who was familiar in using the tool as a clinical mentor for the University where the tool is normally used. One may question whether these improvements were not due to the intervention but due to improvements over time with the physiotherapists learning through experience. The changes in physiotherapist performance (Fig 3A) show measures taken at approximately 12-month intervals, with 1 set of pre-intervention and 2 sets of post-intervention measures. The biggest increase is the 7.3-point difference between pre-intervention and post intervention compared with the 1.8-point difference between the 2 post-intervention measures, pointing to the biggest gains being made due to the intervention rather than just over time.

The findings of the primary outcome measure in this trial are not reflected in the secondary outcome measures of generic HRQL, satisfaction or activation. Possible reasons for this lack of effectiveness may be due to the trial being underpowered for these measures, as the power calculation for this trial was performed on the primary outcome measure of the PSFS. Furthermore, this may have been compounded by missing data being greater for these secondary measures. This was owing to the PSFS data being able to be obtained by the physiotherapist during a telephone call to notify patients who were not present to complete the questionnaire at discharge that a postal questionnaire was being sent. The secondary measures being self-report questionnaires that required the patient to complete and return them meant that they could not be captured by telephone. A second reason may be the responsiveness of the instruments. Studies exploring the comparative responsiveness of the PSFS have demonstrated it to be more responsive than condition-specific outcomes used in the MSK context [66, 92, 93]. While the EQ-5D-5L was selected due to its extensive evidence base and validity as well as being accessible and its broad currency across multiple disciplines, it was not selected as the primary outcome measure on the basis that, as a generic measure of HRQL, it was likely to be less responsive than the patient-specific measure of HRQL, the PSFS [94, 95]. The lack of significant difference in patient satisfaction may well be attributable to a ceiling effect. International data as presented in a meta-analysis of use of the MRPS across outpatient settings in northern Europe, North America, the United Kingdom and Ireland shows patient satisfaction with MSK physiotherapy to be consistently high, with a pooled estimate of 4.44 (95% CI 4.41, 4.46) out of 5.0 [77]. In this trial, the patients in the intervention group reported a mean satisfaction of 4.6 which is higher than the data from the international studies. Ceiling effects are considered to be present if more than 15% of respondents achieve the highest possible total score [96] and indeed, the most common response to the questionnaire was 5.0 out of 5.0, with 14% of the sample submitting this response. While this figure is just outside the cut-off for ceiling effects, with the level of satisfaction so high for the control group, achieving statistically evidence for improvements in satisfaction for the intervention group was extremely challenging. The lack of difference between groups in patient activation may be attributable to the nature of the training, requiring a more specific educational approach on developing patients’ skills in self-management. The intervention in this trial was to enhance the physiotherapists’ clinical reasoning skills within a framework that was explicitly patient-centred. Conceivably, this should encompass empowering the patient’s self-management skills, and equipping them to manage their own health effectively. However, the results of our trial show that while the physiotherapy service improves patients’ levels of activation, in that both arms of the trial showed improvements in PAM scores, the intervention added no value in this area of patient care. The developers of the PAM suggest approaches to training of clinicians specifically tailored to improve their ability to improve patient activation [97].

### Comparison with existing literature

The results of this trial contrast with those of a trial by Overmeer and colleagues [28, 29] investigating the effect of an 8-day university course for physiotherapists on patient outcome. While the education programme did produce changes in clinicians’ attitudes, beliefs and knowledge, there was no effect on patient outcomes. The authors propose that this lack of effect may be due to the delivery of the education being away from the clinical context, and that the educational strategy most likely to change practice behaviour (and therefore clinical outcomes) is to educate within the clinical context and provide direct feedback on the clinician’s encounter with actual patients [28]. This is precisely the strategy that the intervention in the current trial employed and may explain the difference in results.

One trial by Cleland and colleagues [30] has previously demonstrated a positive effect on patient outcomes of an educational intervention to the treating physiotherapists, in the private sector in the United States. Employing a parallel design, both groups of clinicians received education via a 2-day course on the management of neck pain. The intervention group received ongoing education (part of which was delivered in the clinical setting); the control group received no further education. While the changes in pain scores were not significantly different for patients treated by the two groups of physiotherapists, reductions in disability scores were significantly greater (p = 0.019) in patients of the intervention group, with a mean difference of 4.2 points (95% CI 0.69 to 7.7) on the Neck Disability Index. Our trial has similar results to this trial but for a much broader caseload of patients (the focus of Cleland’s trial is only on patients with neck pain and uses a condition-specific measure). Also, the Cleland trial only controlled for the patient variables of age, gender and initial pain and disability scores; with prognostic factors not explored. There was no exploration of therapist factors or performance change and the intervention being delivered to individuals rather than clusters gave the potential for contamination bias (clinicians from different groups worked in the same clinics). It is worth noting that while the educational interventions were quite different between our trial and that of Cleland’s, both were ongoing and involved actual patients within the physiotherapist’s own clinical practice setting, supporting the assertion that ongoing education that occurs within the clinicians’ context is likely to affect patient outcome [28, 29].

Looking outside of the physiotherapy literature at other disciplines shows other examples of where level 4 evidence has been delivered to measure clinical effectiveness. This trial contributes new evidence to this broader literature in terms of the type of level 4 evidence provided, with the PSFS being aligned with the World Health Organisation’s International Classification of Functioning, Disability and Health (ICF) framework [98–100] and measuring functional activities selected by—and therefore highly relevant to—the patient. Analysing the level 4 outcomes used in medicine shows that those most frequently used are the absence of harm, or procedural success measured by time taken. Reviews of simulation-based medical education [18] and residency training on patient outcomes [19] and studies on the effect of baccalaureate degrees on Nursing [20–22] use mortality rates, length of time in theatre, length of stay in hospital, complication rates and patient satisfaction as the patient outcomes to evaluate training and education. This trial provides an alternative pattern for selecting outcomes for educational interventions. Accepting that different disciplines will have different norms and expectations of what to measure, and that surgical approaches carry high risk, the focus on health and function, activation and participation of our trial provides a positive outcomes approach by measuring functional activities identified as meaningful by the patient. Other disciplines could utilise such outcomes alongside their traditional measures to give a more holistic appraisal of the educational interventions under exploration. Furthermore, this trial provides evidence that is robust in that it compares the intervention with an educational control and demonstrates statistical and clinically significant improvements over this control. The reviews of the medical education disciplines cited above found that some of the studies showed no effects, or small effects when compared with other training approaches, or positive effects that were not statistically significant.

Our trial also builds on findings of qualitative studies exploring masters courses where this type of intervention is used, adding quantitative data to the qualitative themes identified. It is important to clarify that this is not a claim for the superiority of quantitative data over the established qualitative findings; rather, it provides new Level 3 outcomes evidence (outcomes which focus on the change in clinical behaviour in routine practice resulting from training) through direct observation of the participants in the clinical setting which is the ideal means of assessment [12]. By utilising a blinded assessor and a validated tool in a randomised controlled trial format we were able to demonstrate statistically significant change across the intervention, thus substantiating the claims of practice change in response to this type of intervention. Furthermore, these statistically significant changes in performance are reflected in statistically significant changes in the primary outcome measure of the patients treated by the participants.

### Clinical Implications

This trial answers the calls from the medical education literature to evidence educational interventions for clinicians with patient outcomes. The clinical effectiveness of the intervention may be due to its nature, evidencing claims and convictions that have been asserted in the medical education literature for some time that training applied in context and incorporating real time feedback for clinicians is most likely to affect patient outcome [63, 101–105]. Clinicians who are seeking to undertake external courses or higher education degrees to develop their level of expertise, may wish to select those that offer modules involving clinical placement or mentoring in the workplace. Hospital managers, rather than reducing training budgets and opportunities [106, 107] might wish to develop and fund training programmes for clinical staff with the goal of achieving improved patient outcomes. Training co-ordinators may wish to review the context within which their training occurs; could it be delivered over a sustained period, more specifically to the learning needs of the clinician, with real time feedback?

### Strengths and weaknesses

This is the first rigorous trial that has measured the clinical effectiveness of an educational intervention not just on clinician performance but also on patient outcomes, using a responsive patient-centred primary outcome measure. The diverse patient population represented here provides generalisability and the trial approach comes from a “realistic evaluation” standpoint, being delivered and measured in an NHS organisation which could be reasonably replicated.

A limitation of this trial is that the patients providing outcome data were required to be recruited by the physiotherapists participating in the trial. It has been highlighted elsewhere that in SWTs where recruitment is extended over time or there is no blinding to the intervention there is a risk of selection bias [108]. Even though clusters were allocated to receive the intervention in a random order and patients were blinded to their therapists’ intervention exposure, it was not possible to blind the participating therapists; introducing the possibility that physiotherapists could have recruited patients more likely to have a positive outcome after their intervention while neglecting to recruit patients more likely to have a negative outcome. The results show that demographics between the intervention and control patients were similar on all factors other than gender (and these factors were included in the regression model for analysis) and so there is no evidence of this occurring; nonetheless, the recruitment of patients by participating physiotherapists is a methodological weakness.

### Unanswered questions and future research

While this trial has shown a positive effect on patient-specific outcomes, it did not have an effect on all outcomes. In particular, the lack of effect on patient activation was surprising, raising the question ‘how do we train our clinicians in order to improve patients’ abilities to manage their own health?’ Future research might also explore the reproduction of similar interventions in broader professional contexts and the subsequent effect on patient outcome.

## Conclusion

This is the first trial to measure the clinical effectiveness of an educational intervention with level 4 outcomes in the field of physiotherapy. It is also the first trial to provide level 4 evidence for the clinical effectiveness of an educational intervention that is utilised in higher education modules in masters degrees aimed at developing expertise in physiotherapy. As such this trial adds to the rich qualitative data supporting such an intervention by providing a quantitative viewpoint and data to show that such an intervention ‘works’ in terms of delivering improved patient outcomes, augmented by the secondary outcome of level 3 evidence demonstrating performance change in physiotherapist clinical reasoning alongside the improvements in patient outcome.

## Supporting information

S1 Checklist Consort checklist. (PDF)

S1 File Ethics protocol. (PDF)
