# Supplementary material for: Evaluation of the clinical effectiveness of a work-based mentoring programme to develop clinical reasoning on patient outcome: A stepped wedge cluster randomised controlled trial
Source: PLoS One. 2019 Jul 31;14(7):e0220110. doi: 10.1371/journal.pone.0220110 (PMC6668791; doi:10.1371/journal.pone.0220110)
Supplement: S1 File — (PDF) [file pone.0220110.s002.pdf]

The Effect of Work-Based Facilitation in Clinical Reasoning on Outcome in Musculo-skeletal Physiotherapy

PhD Proposal / Protocol

Aled Williams

2/22/2012

Version 2.0. REC REFERENCE NO: 12/WA/0078

Contents:

Introduction & Literature Review **3**

- The research problem 3
- Past research on the problem 3
- Limitations of the literature 9
- Questions arising from the literature 10
- Audiences 10

Study Aims & Reasons **11**

- Purpose statement 11
- Research questions 11
- Selection of the research design 12

Research Methods **14**

- Mixed methods definition 14

Quantitative Component **15**

- Type and Definition of Design Used 15
- Scientific background and rationale 15
- Methods – Participants 17
- Methods – Interventions 18
- Objectives 20
- Outcomes 20
- Sample Size 27
- Randomisation 29
- Blinding/Masking 30
- Statistical Methods 30
- Challenges in using this type of design 31
- Variables 32
- Validity approaches 32

Qualitative Component **35**

- Type and Definition of Design Used 35
- Challenges in using this design and how they will be addressed 38
- Examples of use of this type of design 39
- Recruitment, Enrollment & Baseline Data Collection 39
- Data collection & Analysis 39

Economic Evaluation Component **47**

- Rationale for Economic Evaluation 47
- Aim of Economic Evaluation 47
- Design of Economic Evaluation 47
- Economic Evaluation Methods 47
- Data Analysis 49

Potential Ethical Issues **51**

- Clarification of the Participants' Experiences 51
- Ethical Considerations 51
- Underlying Ethical Principles 51
- Gaining Ethical Approval 51

References **55**

Appendices **61**

The Effect of Work-Based Facilitation in Clinical Reasoning on Outcome in Musculo-skeletal Physiotherapy

Introduction

The research problem

In healthcare, the accomplishment of quality patient outcomes is seen as the ultimate goal of all evidence-based practice evaluation (Fineout-Overholt and Johnston, 2007). Outcomes research, put simply, is the assessment of what does and does not work in the delivery of healthcare (Prystowsky and Bordage, 2001) and the education and development of clinicians should be evaluated in order to ascertain whether it actually achieves better health outcomes; this is the assertion of several medical educational researchers (Chen et al., 2004, Fineout-Overholt and Johnston, 2007, Fincher et al., 2010, Kalet et al., 2010, Prystowsky and Bordage, 2001, Whitcomb, 2002b). With steadily increasing health costs, increasing pressure on budgets, greater accountability for training expenditure, ageing patient populations, increasing burdens of chronic illness and rapid changes in healthcare technology and care delivery, the link between education and patient outcome is seen as greater than ever (Kalet et al 2010; Prystowsky and Bordage 2001; Whitcomb 2002). Positively, establishing such links would enable researchers to delineate how the clinical component of healthcare education courses contributes directly to the health of individuals and the public, improving the relevance and impact of medical education research, and enable patients and practitioners to make better-informed, cost-effective healthcare decisions (Kalet 2010; Prystowsky and Bordage 2001). Negatively, neglect of this research topic could lead to reduced external funding of educational research and prompts the editor of one medical education journal to state that priority of publication would be given to research investigating patient outcomes (Whitcomb 2002).

Past research on the problem

<u>Medical Education Literature and Outcomes</u>

So how successful have the calls been for research to meet this objective? Despite the fact that the call for research into this link has been persistent over the last 30 years, a review of the medical education literature suggests that it has been largely unheeded as there appears to be a dearth of literature examining the link between professional education and patient outcome (Chen et al., 2004, Gruppen, 2007, Kalet et al., 2010, Mourad and Redelmeier, 2006, Prystowsky and Bordage, 2001, Whitcomb, 2002a, Magraw et al., 1978). Instead, the focus of educational research has been on trainee performance and trainee satisfaction (Prystowsky and Bordage 2001; Kalet, 2010), and a recent collaboration of US medical schools to set research priorities still identified trainee performance as the highest-rated priority ahead of patient impact (Fincher, 2010). Reasons for the failure of researchers to meet this challenge have been suggested: difficulty in obtaining data due to lack of recording or confidentiality, methodological difficulties such as many confounding variables, limited impetus due to lack of investment in educational research, and the different research paradigms used by medical education and health service researchers leading to a lack of collaboration (Chen, 2004; Kalet, 2010; Prystowsky and Bordage 2001; Whitcomb, 2002).

<u>Education plus other intervention and Outcomes</u>

There are a small number of published research articles which have attempted to measure the effect of educational intervention by assessing the clinical outcomes of the patients treated by the clinicians who

have received the educational interventions. However, these studies do not measure the efficacy of the educational programme *per se*; rather they evaluate treatment approaches to which the educational intervention is attached against usual care or another treatment approach; but they are worthy of attention for this study in that their discussion sections deal helpfully with strengths and weaknesses of the educational approaches utilised.

The first series of studies considered training associated with new low back pain guideline implementation. Bekkering and colleagues (Bekkering et al., 2005), and Engers and colleagues (Engers et al., 2005), performed studies which implemented psychosocial training programmes to assist with the implementation of the Dutch Low Back Pain Guidelines. While Engers et al looked at General Practitioner referral and prescription rates in their cluster RCT, Bekkering – also in a cluster RCT -used patient outcomes (physical functioning, pain, sick leave, coping, and beliefs) to examine the effect of an active education strategy developed for the implementation of clinical guidelines. The results of both cluster RCTs were underwhelming - there were no significant improvements in the patient outcomes of physiotherapists who had received training over the patients of those who had not, and there were no differences in GP behaviour (explanations, referral rates and prescribing patterns) of GPs who had received training over the GPs who had not.

The findings that GP behaviour was unchanged (Engers et al., 2005) is echoed in the physiotherapy literature by a cluster RCT (Stevenson et al., 2006) looking at the change in physiotherapists' management of patients with low back pain following an educational programme. Few significant differences in the treatment options were found between the intervention (evidence-based training programme) and control (standard in-service training) groups post training and little change in what physiotherapists perceived to be important to patient recovery and actual clinical practice following the intervention as measured by physiotherapist discharge summaries. This raises the valid question that if education does not change behaviour of clinicians then how likely is patient outcome to change? (Overmeer et al., 2009).

Two papers (Jellema et al., 2005a, Jellema et al., 2005b) published from the findings of a cluster RCT which used educational programmes to aid clinicians' implementation of psychosocial models of care explored these issues. Jellema and colleagues (Jellema et al., 2005b) compared usual care with a strategy aimed at assessment and modification of psychosocial prognostic factors in 314 patients with sub-acute low back pain carried out by 60 general practitioners in 41 practices. The intervention strategy used two 2.5 hour training sessions given by a general practitioner with recognised expertise in development of and training in psychosocial interventions. The training programme involved theory, role play, feedback on the practised skills, and was reinforced with a treatment manual. While GP attitudes who had received the training became significantly less biomedically orientated (measured on a modified PABS_PT) compared with colleagues receiving usual care, but while treatment contents were significantly different between the groups (Jellema et al., 2005a), no significant differences were found between the groups in patient outcomes (Roland-Morris disability questionnaire scores, perceived recovery, or sick leave) during 12 months of follow-up in the whole group or in relevant subgroups (Jellema et al., 2005b).

<u>Physiotherapy Education and Patient Outcomes</u>

The findings of Jellema and colleagues are very similar to those of another two recently published papers (Overmeer et al., 2011, Overmeer et al., 2009) exploring the education of physiotherapists in an 8-day University course aimed at identifying and addressing psychosocial risk factors in patients with musculoskeletal pain. The RCT randomised course applicants to intervention (course participants) and control (course waiting list). Outcomes (pain, disability, catastrophising, and mood) from patients treated before and after the course were measured in both groups. No significant differences were found between patients of physiotherapists who had attended the course, and those who had not, either in the patient

groups as a whole, or in the patients who were at risk of developing long-term disability (having higher levels of catastrophising or depression). However, physiotherapists' attitudes and beliefs did change - becoming more biopsychosocially and less biomedically orientated, less convinced that pain justifies disability and limitation of activities, and increasing their knowledge and skills on psychosocial risk factors after the training course (measured by the Pain Attitudes and Beliefs scale for physical therapists (PABS_PT) (Houben et al., 2005, Ostelo et al., 2003), the Health Care Providers Pain and Impairment Relationship scale (Houben et al., 2004, Rainville et al., 1995) and patient vignettes constructed by Rainville (Rainville et al., 2000)). This RCT showed that while the education programme did elicit changes in clinicians' attitudes, beliefs and knowledge, there was still no impact on clinical patient outcomes; neither was there any impact on patient satisfaction or perception. However, this study is of note in that the variable under investigation is educational (rather than a treatment approach to which the educational approach is a means to an end), and the measurement is patient outcome.

Only one other study (Cleland et al., 2009) investigated the impact on patient outcomes of an educational intervention to the treating physiotherapists. This study is worthy of particular note in that in contrast with all the previous studies discussed, it demonstrated a significantly positive impact. In this RCT, clinical outcomes (pain and disability) achieved by groups of patients treated in pre-training and post-training periods by physiotherapists who attended a 2-day course on the management of neck pain were compared. After the course, physiotherapists were randomised to intervention or control. In the intervention group, physiotherapists received ongoing education consisting of small group sessions and an educational outreach session in which clinicians received training in their clinical settings; the control group received no further education. While the changes in numerical rating scale measures of pain did not differ for patients treated by the two groups of physiotherapists during the study period, patients treated by physiotherapists who received additional training exhibited significantly greater reductions in disability during the study period (pre-training to post-training) than those treated by therapists who did not; they also required fewer visits during the post-training period.

The question must be asked why the educational intervention was successful in this study (Cleland et al., 2009) as opposed to the lack of impact on patient outcomes from all the other studies (Bekkering et al., 2005, Engers et al., 2005, Overmeer et al., 2011, Jellema et al., 2005b, Stevenson et al., 2006). Questions regarding the content of guidelines and treatment approaches should always be permissible, but do call into question all the underpinning evidence that informed the development of those guidelines and treatment approaches. The more obvious and immediate area of focus is the distinguishing features of the educational approaches implemented. The length of training is cited as one potential reason for disappointing results (Jellema et al., 2005a); while time differences in these studies vary from 2 hours (Engers et al., 2005) to 8 days (Overmeer et al., 2011), the successful study (Cleland et al., 2009) used a 2 day course followed by two 1.5 hour meetings within the following 4-7 weeks and a clinical outreach visit (1 hour co-treatment of a patient) followed by discussion, which sits between the two extremes. The two distinguishing features of the successful approach are that the education programme was ongoing, rather than start and finish, and included an outreach visit included a 1-hour co-treatment of an actual patient with neck pain in the physiotherapist's own clinical practice setting with the principal investigator of the study. Overmeer and colleagues (Overmeer et al., 2011) - citing this study - suggest that this may be the key element, where education "...in a physical therapist's clinical environment and with an actual patient may provide the direct clinical feedback needed to change actual behaviour and thereby create the possibility of improved outcomes in patients" (p 815).

<u>Expertise Development and Outcomes:</u>

Outside of the medical education literature, there has been some interesting work examining outcomes research and professional development in the expertise literature. In expertise research an expert has been defined as 'someone capable of doing the right thing at the right time' (Holyoak, 1991), or as 'someone who performs at the level of an experienced professional' (Rikers et al., 2005). Experts have also been defined as top performers who excel in a particular field, such as elite athletes or musicians, or – interestingly - those clinicians who achieve the best clinical outcomes (Rothstein, 1999). Much of the study of clinical expertise in healthcare has relied on the assumption of experience being a critical factor (Benner et al., 1997, Jensen et al., 2000, Dreyfus and Dreyfus, 1996). As a result, much of the research has been performed on samples of practitioners who have years experience, or seniority, or who are expert by reputation (Benner et al., 1996, Benner et al., 1999, Gwyer et al., 2004, Shepard et al., 1999).

This assumption was subsequently challenged, however, in the study of expertise in physiotherapy by Linda Resnik and Gail Jensen (Resnik and Jensen, 2003), who used clinical outcomes to define expertise. Their study incorporated a retrospective analysis of health related quality of life outcome data (the overall health status measure) from the Focus On Therapeutic Outcomes Inc (FOTO) database, to calculate mean patient outcomes for each therapist participating in the database. Using a generalized linear model to control for patient factors on outcomes such as patient age, severity of condition, sex, onset of condition, number of surgeries for condition, reimbursement, exercise history, and employment status, the authors predicted outcomes for patients after linear modelling. By looking at differences between actual and predicted outcome scores, the authors were able to identify an “expert group” of therapists (the top 10% of therapists whose patients had the highest mean outcome scores) and an “average group” of therapists (the 10% of therapists whose patients had average - 45th-55th percentiles - mean outcome scores). Interestingly, they discovered that years experience between “expert” and “average” physiotherapists were similar.

Within the clinical expertise literature is the claim that the key behaviours of expertise can be identified, nurtured and taught (Resnik, 2007, Jensen et al., 2008, Jensen et al., 2007b, Jensen et al., 2007c, Purtilo, 2007). If this assertion is true, then this is certainly of interest to the research problem, because if patient outcomes can be used to identify expert clinicians, it stands to reason that development of traits of expertise within clinicians will yield improved patient outcomes. While the key behaviours of physiotherapy expertise have indeed been identified in qualitative studies (Resnik and Jensen, 2003, Resnik, 2007, Jensen et al., 2000, Jensen et al., 2007b) the assertion that such behaviours can be taught and nurtured is apparently based on personal conviction, as no empirical evidence is cited in support of it. It is possible that it arises from interview data from both Jensen’s and Resnik’s research into physiotherapists who were experts in the musculoskeletal field (Resnik and Jensen, 2003, Jensen et al., 2008, Jensen et al., 2007a, Resnik, 2007). The authors identified that every expert who was interviewed mentioned clinical mentors who had influenced their thinking and practice, and attributed much of their understanding to working with mentors who facilitated their clinical thinking and reasoning processes.

<u>Master’s level clinical practice</u>

The development of expertise by means of Master’s education is an area of literature which picks up many of these themes and has investigated them to differing extents. Of course, any method chosen for continuing professional development should - in theory - develop clinical expertise. However, there are observations made as to why such development might not occur. The circular tendency for clinicians to experience what they expect to experience can lead to difficulties in learning and enhancement of practice, further exacerbated by isolated clinical practice within treatment rooms or screened cubicles (Dall’Alba and Sandberg, 2006, Petty and Morley, 2009). Clinicians may also routinely apply assessment and treatment techniques and strategies to patients, having developed them automatically or habitually (Eraut, 2006a, Eraut, 2005, Eraut, 2004, Eraut, 2001); this lack of reflection and deliberation will limit learning and

development, and may be exacerbated by working in organizations where time pressures are brought to bear as efficiency and patient through-put are the priority. Eraut (Eraut, 2006a, Eraut, 2006b) also points out that the complexity and uncertainty of the problems being managed in clinical practice means that for learning to occur there is a requirement that the clinician receives accurate and specific feedback on his/her clinical decisions. Much CPD activity occurs away from patients and the clinical practice context (Petty and Morley, 2009); if learning experiences are situated where they will be applied, their value is much higher (Fish and Coles, 1998; Billett, 2004;(Dall'Alba and Sandberg, 2006, Johns, 2010) – in short, to enhance clinical practice, learning needs to occur in clinical practice (Petty and Morley 2009(Petty et al., 2011a)). Also, the scope of many short courses or in-service training may be limited to a narrow aspect of clinical practice. For example, a weekend course to develop technical aspects – for example enhancing hands-on skills – or an evening lecture to enhance knowledge (discussion of literature on a specific topic). While these are not to be undervalued, the clinician needs to acknowledge that clinical practice expertise is more than technical proficiency and propositional knowledge (Petty and Morley, 2009; Jensen et al 2007). Procedural knowledge, for example, skills at developing effective therapeutic relationships with patients or how to make clinical decisions in response to patient cues are not addressed by such CPD activities (Edwards and Jones, 2007, Petty and Morley, 2009).

The literature evaluating the impact of Master's education has been wide-ranging and also gives some helpful insight into what may be the strongest component of this form of CPD and the most likely to elicit change in clinical practice and patient outcome. A series of studies using mixed methods by Rushton and Lindsay explored the role of Master's postgraduate courses in developing clinical expertise (Rushton and Lindsay, 2007a), the clinical placement component of Master's courses (Rushton and Lindsay, 2007b), defined the behaviours that are indicative of the construct of masters level clinical practice (Rushton and Lindsay, 2008), and of masters level manipulative physiotherapy clinical practice (Rushton and Lindsay, 2010). The survey in the first of these publications used the whole population of course tutors responsible for Master's courses in the selected professions of Nursing, Occupational Therapy, Speech and Language Therapy, Podiatry, Physiotherapy and Radiography. 148 questionnaires were submitted representing 132 courses (a response rate of 86.5%) and demonstrated the different means used to develop clinical expertise, courses using one or all of theoretical components (90%), mentoring (46%) and clinical placement (39%) as well as other means. Professions varied in emphasis, a theme picked up on in a subsequent publication from the same study (Rushton and Lindsay, 2007b) which further explored the use of clinical placements in developing clinical expertise. The majority of Physiotherapy (52%) and Radiography courses (91%) used clinical placements, with less usage of this method in Nursing (18%) and Interdisciplinary programmes (14%) and none at all in Occupational Therapy, Podiatry and Speech and Language Therapy. The qualitative data analysed in this study produced themes of clinical reasoning, communication, and development of role and skills as characteristic of Master's level practice. These themes were built upon in a further study (Rushton and Lindsay, 2008) which used a modified Delphi study to define the behaviours that are indicative of the construct of Master's level clinical practice. The sample of tutors from Master's courses in the United Kingdom which aim to develop clinical expertise and which directly assess clinical practice by means of a clinical placement suggested and ranked behaviours indicative of Master's level clinical practice over the 3 rounds of the Delphi methodology. 20 behaviours remaining at the end of the 3rd round demonstrated good measurement validity and external validity, and the top ranked behaviours of course tutors were the critical use of evidence to inform practice, high levels of clinical reasoning skills, and critical analysis in approach to practice. This construct then formed the basis of a further study (Rushton and Lindsay, 2010) which used multiple methods of documentary analysis, interviews, and participant observation of clinical tutors and students in one case of a Master's course in manipulative physiotherapy in the UK. In this study, the findings showed that the most important behaviour associated with the construct for manipulative physiotherapy was a high level of clinical reasoning.

These studies provide valuable information to the present study on several levels. Firstly, they provide a valid construct against which to measure performance and behaviour changes in clinicians who receive educational interventions. Secondly, they provide data and information on a method of education (clinical placement) and consensus for the emphasis of that education (clinical reasoning skills being seen as most important). Thirdly, the identified behaviours seem to address the examples given by other authors, (Jensen et al., 2007a, Petty and Morley, 2009) that are unlikely to be covered by other, "usual" forms of CPD (Petty et al., 2011a).

This understanding is enhanced by a recent study (Petty et al., 2011b) which aimed to explain the learning process experienced by physiotherapists on completion of a Master's course in Manipulative Therapy. Analysing semi-structured interviews of 11 alumni from one 'case' course, the authors developed a substantive theory of the learning transition, which they describe as a journey from a position of routine clinical practice which is therapist-centred and associated with uncritical practice knowledge to one of flexible and tailored patient-centred practice, enabled by critical understanding of practice knowledge and the capability to learn in, and from, practice. This study provides interesting and helpful detail on clinicians' expectations going into the learning experience, reactions to the learning experience, and factors which moderate the whole process. However, the most interesting conclusion from the perspective of the proposed research project is the finding that mentorship in clinical practice was the primary means of facilitating critical evaluation of practice knowledge and therefore development towards clinical expertise.

However, while the use of mentorship in the context of clinical placement is established practice in Master's programmes seeking to develop expert practice and has strong support from qualitative literature, the development of clinical expertise by this method has not been tested prospectively and empirically – that by mentoring physiotherapy clinicians, the key features of expertise are actually nurtured, developed and taught, resulting in an improvement in clinical outcomes for their patients as they journey toward expertise.

Clinical Reasoning Literature:

The importance of context is also underlined in the clinical reasoning literature. Clinical reasoning has been defined as a context-dependent way of thinking and decision making in professional practice to guide practice actions (Higgs and Jones, 2008) and the research into expertise in physiotherapy consistently identifies clinical reasoning as a critical component (Edwards et al., 2004, Jones et al., 2008, Jones and Rivett, 2004, Higgs and Jones, 2008, Jensen et al., 2007c, Jensen et al., 2007b, Resnik, 2007). In addition, the importance of clinical reasoning has been highlighted (as discussed above) in work defining Masters level clinical practice in healthcare in the United Kingdom, where the sample of tutors of all Masters courses for healthcare professionals in the UK prioritised a high level of clinical reasoning skills as the most important behaviour to the construct of Masters level clinical practice (Rushton and Lindsay, 2008). A high level of clinical reasoning skills as the most important behaviour was also identified in the subsequent study of the construct validity of Masters level manipulative physiotherapy (Rushton and Lindsay, 2010). Furthermore, a recent study into the research priorities for postgraduate theses in musculoskeletal physiotherapy internationally (Rushton and Moore, 2009), identified - from a sample of course tutors and expert clinicians nominated by member organizations of the International Federation of Orthopaedic Manipulative Physical Therapists (IFOMPT) – research questions on clinical reasoning processes and skills amongst the most important priorities.

Authors in both the expertise and the clinical reasoning literature, warn against studies of clinical reasoning that "take place in a vacuum" on the basis that decontextualised clinical cases do not reflect clinical reasoning processes and behaviours in natural settings, and this emphasis of reasoning and decision-making in context is echoed by a multitude of authors on the topic of clinical reasoning (Downing and Hunter, 2003,

Higgs et al., 2001, Higgs and Loftus, 2008, Loftus and Smith, 2008, Christensen et al., 2008, Edwards and Jones, 2007, Fish and Higgs, 2008, Boshuizen and Schmidt, 2008, Crespo et al., 2004, Jensen et al., 2008, Schön, 1983, Whiteford and Wright St Clair, 2005). Therefore, if the key behaviours of expertise can be nurtured and taught, and clinical reasoning is one of those key behaviours, then any educational strategies to develop clinical reasoning skills should be applied and studied within in the clinical context. Such a contextual strategy is indeed applied by Masters level courses in manipulative physiotherapy, where a clinical placement is implemented as an essential aspect of the educational programme. The rationale for this is outlined in the educational standards document of IFOMPT (IFOMPT, 2008), a non-government International Manipulative Therapy Federation representing international collaboration in Manipulative Therapy and a recognised sub group of the World Confederation for Physical Therapy (WCPT), which in turn is a part of the World Health Organization (WHO). A minimum of 150 hours of mentored clinical practice is recommended to students, where the Clinical Mentor is a member of the Member Organization of IFOMT (in the UK, the member organization is the Musculoskeletal Association of Chartered Physiotherapists, to which all the Masters programmes are affiliated). This is with the goal of achieving the competencies identified by the educational standards document, which specifies that "all programmes should be underpinned by sound clinical reasoning." The value of this approach has also been expressed and demonstrated in qualitative studies (Petty et al., 2011b, Rushton and Lindsay, 2010) as discussed in the previous section.

Limitations in the Literature.

To summarise by bringing these four threads of literature (medical education, expertise, Master's level practice and clinical reasoning) together, it is apparent that within the context of healthcare education research, that while much work has been performed on subject performance, there is a paucity of research into the link between education and patient outcome, despite numerous calls for such research.

The expertise literature within physiotherapy has linked patient outcome with therapist performance in using patient outcome as a means of defining therapist expertise. While this qualitative work has been excellent in using grounded theory in exploring traits of clinical expertise, the conviction expressed that these traits can be nurtured, taught and developed, particularly within the clinical context, does not call on any prospective study which has been undertaken to establish this.

Literature exploring Master's courses for expertise development, including manipulative physiotherapy, in the UK and worldwide, demonstrate the established use of mentorship within the clinical context to develop knowledge, skills and attributes, in the form of clinical placements. This concurs with the consensus from the clinical reasoning literature of the importance of context for any intervention directed at improving clinical reasoning. This literature also provides a recognized and widely practiced intervention for clinical reasoning and expertise development, where improvements in subject performance are measured against competencies and practice descriptors. However, to date no research articles demonstrating quantitative assessment of the impact of this intervention on patient outcome have been published.

The dearth of research into the link between healthcare education and patient outcome has led to the recognition by leading members of the medical education community that "too much of the medical education research has not been designed to address the most important research questions" (Whitcomb, 2002). Indeed, ensuring that the measurement of clinicians' competence and skills is based on the ability to translate competencies into effective patient outcomes is seen as "the ultimate goal" of healthcare education (Kalet, 2010). It is this specific deficiency that this study aims to address in order to achieve this goal, and so to provide a unique contribution to the scholarly literature.

Questions arising from the literature

Several questions arise from this literature. Firstly, the finding from the medical education literature (Chen et al., 2004, Gruppen, 2007, Kalet et al., 2010, Mourad and Redelmeier, 2006, Prystowsky and Bordage, 2001, Whitcomb, 2002a, Magraw et al., 1978) that there is a dearth of literature examining the link between professional education and patient outcome despite the fact that the call for research into this link has been persistent but largely unheeded over the last 30 years raises two obvious questions; can methodological barriers be overcome in order to undertake a study of the impact on patient outcomes of an educational intervention? And do findings of improved clinician performance correlate with improvements in patient outcomes? Secondly, the finding from the expertise literature (Purtilo, 2007, Jensen et al., 2007c, Jensen et al., 2000, Resnik, 2007, Resnik and Jensen, 2003) that every expert who was interviewed mentioned clinical mentors who had influenced their thinking and practice, and attributed much of their understanding to working with mentors who facilitated their clinical thinking and reasoning processes raises the question of whether clinical mentoring can identify, nurture and teach the key behaviours of expertise (including clinical reasoning) as is asserted by the authors? And finally, the finding from the clinical reasoning literature (Downing and Hunter, 2003, Higgs et al., 2001, Higgs and Loftus, 2008, Loftus and Smith, 2008, Christensen et al., 2008, Edwards and Jones, 2007, Fish and Higgs, 2008, Boshuizen and Schmidt, 2008, Crespo et al., 2004, Jensen et al., 2008, Schön, 1983, Whiteford and Wright St Clair, 2005, Rushton and Lindsay, 2010, Rushton and Lindsay, 2008, Rushton and Moore, 2009) of the importance of context in any studies of clinical reasoning, and the current international usage of clinical mentoring programmes in IFOMPT affiliated Masters level education courses raises the question of whether such clinical mentoring programmes would provide a context dependent way of facilitating therapist's reasoning to deliver not only improvements in therapist performance but also greater improvements in patient outcomes?

Audiences

This research has a broad potential scope of audiences for whom it has relevance. From the field of manipulative physiotherapy, through physiotherapy in general, to all fields of healthcare, the topics of education, expertise and clinical reasoning are all potentially impacted by this research. There is relevance for clinicians seeking to improve their clinical reasoning and level of expertise, for educators seeking evidence of impacts in patient outcome from educational interventions, and for health service budget-holders seeking cost-effective ways of staff development and improving patient outcomes.

Study Aims & Reasons

Purpose Statement:

The intent of this study is to assess the effect of work-based facilitation in clinical reasoning on outcomes in musculo-skeletal physiotherapy, to test the theory that clinical reasoning can be nurtured and taught, and that development of this feature of expertise will positively impact patient outcomes.

In the study, validated outcome measures of function, health-related quality of life, symptomology, patient satisfaction and self-efficacy will be used to measure the relationship between facilitation in clinical reasoning via a clinical mentoring programme and patient outcome, while exploring the relationships in the data with the factors of patient age, patient condition, patient prognostic barriers, therapist participation, usual therapist training and organisational context for static musculoskeletal physiotherapists working in a large hospital-based outpatient service.

At the same time, the process of clinical reasoning facilitation will be explored using qualitative interviews with the clinically mentored staff, qualitative interviews with their clinical mentors, and observations of the mentoring process.

Research Questions

The following research questions will be answered.

1. Does facilitation of clinical reasoning skills via a clinical mentoring programme relate to improved patient outcomes, in physiotherapists working in musculoskeletal outpatient departments?

Sub questions:

- How do the physiotherapists rate on clinical reasoning skills before and after a clinical mentoring programme to facilitate clinical reasoning?
- What are the physiotherapists' patient outcomes scores following a clinical mentoring programme to facilitate clinical reasoning?
- What are the physiotherapists' patient outcome scores with the receipt of "usual" training?
- What are the demographics and prognostic barriers of the patients of the physiotherapists whose outcomes are measured?

2. Does facilitation of clinical reasoning skills via a clinical mentoring programme relate to improved physiotherapist performance in clinical reasoning?

3. To what extent and in what ways do qualitative interviews with participating physiotherapists and clinical mentors serve to contribute to a more comprehensive and nuanced understanding of this relationship between clinical reasoning skills, and patient outcomes?

Sub questions:

- To what extent do physiotherapists value and engage with a clinical mentoring programme to improve clinical reasoning skills?
- How do participants perceive their professional development – what do they value, how do they think their clinical practice has developed, how do they feel their clinical reasoning has changed over time, and what events stimulated these changes?

Selection of the research design

Multiple factors inform and influence the decision of which research design to select in studying a research topic, such as the researcher's worldview assumptions, strategies or procedures of inquiry, and specific methods of data collection, analysis and interpretation. The nature of the research problem, the researchers' personal experiences, and the audiences for the study will also influence the selection process (Creswell, 2009). Qualitative and quantitative approaches should be viewed as different ends on a continuum rather than polar opposites or dichotomies (Newman and Benz, 1998), where qualitative research is a means for exploring and understanding the meaning individuals or groups ascribe to a social or human problem and quantitative research is a means for testing objective theories by examining the relationship among variables, and then measuring the variables and analysing the measurements (Creswell, 2009). Mixed methods research is more than simply collecting and analysing both kinds of data. Rather, it is an approach to inquiry involving philosophical assumptions, combining both qualitative and quantitative forms, using both approaches in tandem so that the overall strength of a study is greater than either qualitative or quantitative research in isolation (Creswell and Plano Clark, 2007).

Philosophical Worldview

Creswell (2009) recommends that researchers identify their epistemological stance or "worldview" in order to identify the reasons behind the choice of research design. From the four categories that Creswell specifies (Postpositivism, Constructivism, Advocacy/Participatory and Pragmatism) it would appear that the epistemological stance of this research is pragmatic, being concerned with applications (what works in obtaining improved patient outcomes) and solutions to problems (the lack of research linking education to patient outcomes) (Patton, 2002). The pragmatic worldview focuses attention on the research problem in social science research and uses multiple approaches to derive knowledge about the problem (Rossman and Wilson, 1985, Tashakkori and Teddlie, 1998, Morgan, 2007, Bergman, 2010, Patton, 1990, Cherryholmes, 1992). The intended consequences form the basis for the "what" and "how" to research for pragmatic researchers.

Strategy of Inquiry

The selection of a strategy of inquiry – or research methodology – is important as these qualitative, quantitative, and mixed methods designs or models provide procedures of the research design with specific direction (Creswell, 2007, Mertens, 2009). While this study involves the testing of a theory (that clinical reasoning can be nurtured, taught and developed) which would indicate the use of a quantitative strategy, it is recognised that all methods have weaknesses, and that a quantitative approach by itself would be inadequate to best understand this particular research problem (the impact of educational input on patient outcome) and that combining the strengths of both quantitative and qualitative research could provide the best understanding. Indeed, Kalet and colleagues (Kalet et al., 2010) make this assertion in discussing studies into this research problem of linking patient outcome to clinical education: "...measures of important patient outcomes is an important initial step that likely requires a mixed-methods approach because no single measurement strategy is adequate to the task. (p847)" The reason for combining both quantitative and qualitative data in this study is to better understand this research problem by converging both quantitative (broad numeric trends) and qualitative (detailed views) data by triangulating data from changes in therapist performance, mentor and mentee experiences, and changes in patient outcomes. Converging qualitative data (qualitative quotes from the physiotherapists' and mentors' perspectives) with quantitative data (statistical analysis of patient outcome and therapist performance data) would allow for comprehensive analysis of the research problem, as well as for more thorough exploration of the intervention itself and factors such as therapist participation.

Research Methods

Having established a purpose and rationale for why quantitative and qualitative data need to be mixed in the first place, the specific procedures require selection; whether sequential, concurrent or transformative mixed methods procedures (Creswell & Plano Clark, 2007). Concurrent mixed methods procedures appear to be the most appropriate for this research problem, due to the desire to converge data to give a comprehensive understanding. In this design, the quantitative and qualitative data collection occurs at the same time and then the information is integrated in the interpretation of the overall results. Also, in this design, one smaller form of data may be embedded within another larger data collection by the researcher in order to analyse different types of questions - the qualitative addresses the clinical mentoring process while the quantitative addresses the outcomes (Creswell, 2009). This fits well with the purpose of this research study, where the larger quantitative component of comprehensive patient outcome analysis is augmented by qualitative inquiry into the process of clinical reasoning facilitation. Specifically, the quantitative research method of an experimental design comparing facilitation in clinical reasoning to usual training on improvements in patient outcomes will be facilitated by the qualitative data obtained from in-depth interviews with participant physiotherapists and their mentors, and inferences will be made across both the quantitative and qualitative databases.

Personal and Professional Perspectives

The personal experiences and training of the researcher will also influence the choice of approach (Creswell, 2009). My own professional and educational journey to date has been strongly associated with technical, scientific writing, statistical analysis, and critical appraisal of articles within quantitative journals. Therefore my leaning would most naturally be to choose a quantitative design. The quantitative approach would also lend itself to audiences of manipulative physiotherapy journals, which are predominantly quantitative, where this study has immediate application. However, as already outlined, the complex nature of the research problem, and the pragmatic stance of this research (looking at what works) requires collection of diverse data in order to best provide a comprehensive analysis. Also, from an audience perspective, much of the work on expertise and clinical reasoning has been exploratory qualitative work, and qualitative data collection of characteristics identified in that literature would help to pull the different strands of research together.

Research Methods

Mixed Methods Definition:

Mixed methods research has been defined as an approach to inquiry that combines or associates both qualitative and quantitative forms, involving philosophical assumptions, the use of qualitative and quantitative approaches, and the mixing of both approaches in a study (Creswell, 2009).

Mixed methods strategies of inquiry fall into three main groups – sequential, concurrent and transformative (Creswell, 2009, Bazeley, 2009, Bergman, 2010, O'Cathain et al., 2010). In sequential mixed methods strategies, designs in which one type of data provides a basis for the collection of another type of data are used (Tashakkori and Teddlie, 1998). Other terms in the literature which refer to such approaches include "sequential study", "two-phase design", and "sequential QUAN-QUAL analysis". In concurrent mixed methods strategies, multi-strand designs in which both qualitative and quantitative data are collected and analysed to answer a research question which can be either qualitative or quantitative, and inferences are finalized on the basis of both data analysis results. The two types of data are collected independently at the same time or with a time lag (Tashakkori and Teddlie, 2010). Transformative mixed methods strategies have a transformative or advocacy purpose in both perspective and outcomes, seeking to promote change at levels ranging from the personal to the political (Creswell, 2009).

Specifically, this study will utilize a concurrent embedded mixed methods design where both forms of data will be collected at the same time and then merged to provide a comprehensive analysis of the research problem. The larger quantitative component is symbolised in upper case in figure 1, with the embedded qualitative component in lower case. This strategy will be employed as it is the most appropriate strategy with which to answer the research questions identified, with the collection of both types of data (qualitative observations of the intervention, and interviews with participants about the intervention, occurring concurrently with quantitative therapist and patient outcomes data following the intervention) allowing the different types of questions to be answered by the different forms of data analysis (broadly, the qualitative addressing the process while the quantitative the outcomes (Creswell, 2009)). This rationale will be developed further in the individual sections on the Quantitative and Qualitative components of the study, which will now be outlined in turn.

Figure 1: Concurrent Embedded Design (Creswell et al., 2003)

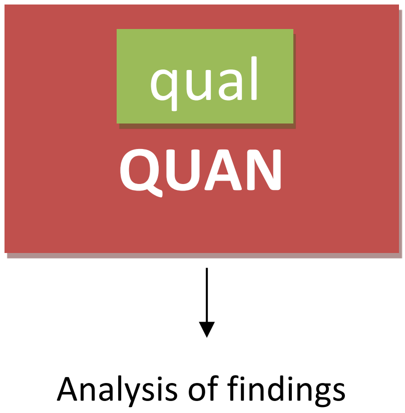

Quantitative Component

Type and Definition of Design Used:

The quantitative component of this study will take the form of an experimental design, namely a stepped-wedge design, a type of cluster randomised controlled trial (CRCT). For the sake of clarity, the Consort statement for CRCTs (Campbell, 2004, Campbell et al., 2005) will be used to structure this section.

Scientific background and rationale.

When seeking to establish a cause-effect relationship between an intervention (in this case, clinical reasoning facilitation with physiotherapists) and an outcome (patient outcomes for those physiotherapists) the most robust research design is a randomized controlled trial (RCT) (Mdege et al, 2011; Torgerson & Torgerson, 2008). The RCT is widely accepted as the best method of evaluating clinical effectiveness within Cochrane systematic reviews (Pope et al, 2007) and has been increasingly used to evaluate non-clinical interventions (Brown and Lilford, 2006). RCT designs can be classified according to their study design: parallel group trials, where each participant is randomized to one of the intervention arms; crossover trials where each participant receives each intervention in a random sequence; cluster trials where clusters of individuals (e.g. doctor's surgeries) are randomly allocated to different study arms; factorial trials where participants are randomly assigned to individual interventions or a combination of interventions, and split body trials where separate body parts within each participant are randomized (Hopewell et al., 2010, Chan and Altman, 2005, Stolberg et al., 2004).

It has been stated that Cluster RCTs (CRCT) are widely recognized to be more appropriate than individually randomized parallel studies for evaluating family or community based interventions, and - interestingly for this study – interventions to change practitioner behaviour (Eldridge et al., 2008, Eldridge et al., 2004) and educational approaches targeting the health professional rather than the individual patient (Campbell et al., 2000). The reason that educational interventions lend themselves to CRCT evaluation is that the training of clinicians in order to impact their patients is a cluster-level intervention which is one of the scientific reasons for employing a CRCT (DiGuiseppi and Coupland, 2010, Gielen et al., 2001, Edwards et al., 1999). In this study the intervention is applied at cluster-level to groups of physiotherapists (by department) but the effect of this intervention on patient outcomes will require measurement on clusters of patients. There are further scientific reasons for clustering in this study. Firstly, to prevent contamination – in this case, even though the intervention is individualized to participating physiotherapists according to identified reasoning errors, an individual design would introduce the possibility of contamination by participating physiotherapists in different arms of the trial working together and discussing the intervention; by clustering physiotherapists by site and these clusters being the unit of randomization, this risk is minimized (Campbell, 2004, DiGuiseppi and Coupland, 2010, Edwards et al., 1999, Hemming et al., 2011). Secondly, as patient outcomes are the measurement criteria for evaluation, clusters of patients will be required to provide this data because it is not possible to individually randomize patients to different arms; the delivery of the training to physiotherapists will influence all patients under that physiotherapist's care from that time point on, and therefore these patients are more likely to respond in a similar manner (Campbell, 2004).

There are further reasons for using a CRCT which relate to the specific type of CRCT selected for use. CRCTs can use parallel, crossover or stepped-wedge designs (Hughes, 2007). A parallel design randomizes clusters to either intervention or control; a crossover design use two time points where half of the clusters receive the intervention at each point; a stepped-wedge design uses multiple time points in which the intervention is rolled-out sequentially to the trial participants (either as individuals or clusters of individuals) (Hussey and Hughes, 2007, Brown and Lilford, 2006). Effectively, the stepped wedge is a one-way crossover cluster trial

where all individuals or groups receive the intervention, but the order in which the different individuals or clusters receive the intervention is randomly allocated (Mdege et al., 2011). Data collection occurs at each point where a new group receives the intervention. This is illustrated in figure 2 below where 5 clusters cross over from control to intervention across 6 time points.

Figure 2: The Stepped-Wedge Design

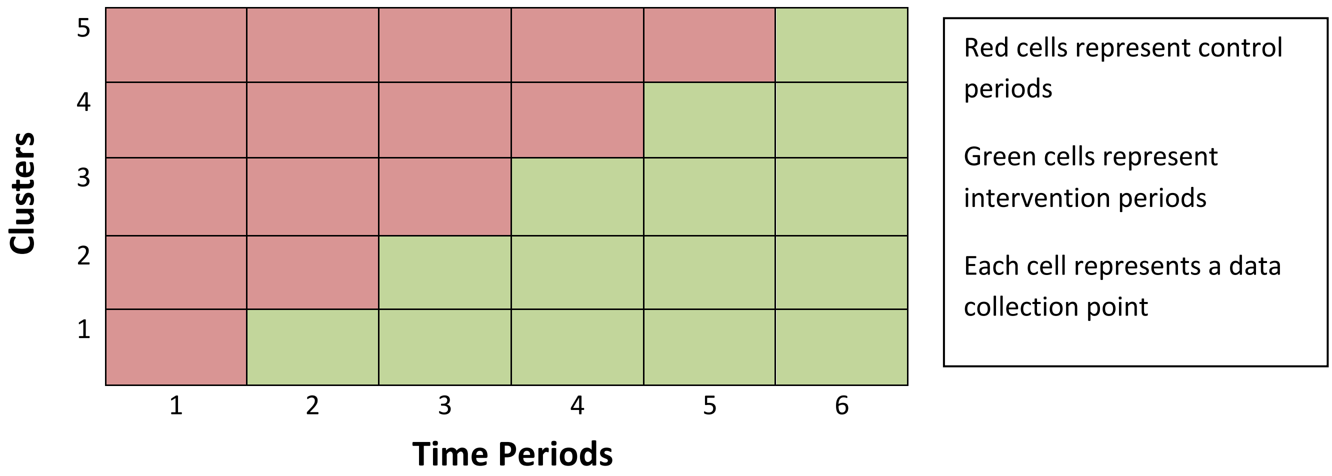

A parallel design for this study was deemed unsuitable for both scientific and logistical reasons. Scientifically, because of the possibility, even likelihood, that in a parallel design the different groups of physiotherapists would be found to be unbalanced on some covariates, whereas the crossover designs have balance, reducing the influence of confounding variables because each physiotherapist subject serves as his or her own control (Brown et al., 2008). This is particularly important for this study as it could be argued that individual therapist attributes could have the greatest impact on patient outcomes. Logistically, the statistical efficiency of crossover designs requires fewer subjects than parallel designs; in a study requiring the use of available qualified clinical staff, and highly qualified clinical mentors, this would appear to be a valid consideration for design selection (Jones and Kenward, 2003). The stepped-wedge design was selected for four main reasons:

1. There is not a belief of equipoise – that is, it is rational to believe that the intervention is likely to result in good rather than harm for both the participating physiotherapists, who will receive Master's level mentoring, and the clusters of patients "exposed" to the physiotherapists who have received training, making a parallel CRCT ethically less acceptable in that it would withhold the intervention from a larger proportion of physiotherapists and patients (Mdege et al, 2011; Brown & Lilford, 2006).
2. While a standard crossover CRCT would also satisfy this ethical concern, it would require the delivery of the intervention at the same time; this is logistically and practically difficult as the intervention (clinical reasoning facilitation via a mentoring programme) requires both specialist input (experienced MACP qualified mentors) and significant time input (150 hours of clinical mentoring), making the stepped-wedge design preferable as the intervention can be delivered in stages (Mdege et al, 2011; Hussey and Hughes, 2007; Brown and Lilford, 2006).
3. The stepped wedge design has been used for scientific reasons such as to allow detection of underlying trends or control for time effects (Brown and Lilford, 2006, Mdege et al., 2011); this is of interest in the current study, as the multiple time points of data collection will allow for investigation of time effects to answer relevant questions– for example, does the effect dissipate over time, or does the intervention require time to consolidate and therefore have impact on patient outcomes.
4. Another advantage of the stepped wedge design over a parallel CRCT is that it requires fewer clusters (Hussey & Hughes, 2007), and it maximises power as the intervention effect is estimated on both between-cluster and within-cluster comparisons (Mdege et al, 2011; Pearson et al, 2010).

Methods - Participants

There are some key questions to be clear in answering in the design of CRCTs: what is the unit of randomization, how and to whom is the intervention delivered and how and on whom is the outcome measured (Hussey and Hughes, 2007)? In this study, the physiotherapy sites (departments) are the unit of randomisation, the intervention is delivered to participating physiotherapists in these clusters, and the outcome is measured on clusters of consenting patients who are treated by these physiotherapists.

<u>Eligibility criteria for participant clusters</u>

As expertise is generally accepted to be context dependent (Alderson, 2010, Benner et al., 1996, Edwards and Jones, 2007, Eraut, 2006b, Jensen et al., 2007b, Resnik, 2007), all qualified physiotherapy staff from the Cardiff and Vale University Health Board whose majority of time (i.e. greater than 50%) is practised inside the musculoskeletal outpatient context will be eligible to participate in this study. There are two main exclusion criteria, namely members of staff who have already undertaken postgraduate placements as part of Master's education and so would have received similar clinical reasoning facilitation, and rotational staff due to the fact that they will not be present in the department for long enough for both interventions and long term follow up. Currently, the maximum potential number of participating therapists would be 18. It is envisaged that 12 physiotherapists would realistically be recruited and organized into 3 clusters of physiotherapy staff (clustering by site).

<u>Eligibility criteria for patient clusters</u>

Consecutive consenting patients attending the outpatient musculoskeletal physiotherapy service at the Cardiff and Vale University Health Board for treatment by the participating physiotherapists during data collection periods towards the end of each step will be eligible to participate. The main exclusion criteria would be patients under 18 years of age, and patients who are not English-literate due to the validity of the Outcome Measures being established only for the English language and for adults.

<u>Settings and locations where the data will be collected</u>

Patient outcome data will be collected at the first and last appointment of physiotherapy treatment at the six sites of outpatient musculoskeletal physiotherapy service delivery for the Cardiff and Vale University Health Board: The Barry Hospital, Cardiff Royal Infirmary, University Hospital Llandough, St. David's Hospital, University Hospital of Wales and Whitchurch Hospital. The 6 sites are organized into pairs for training purposes, and so to further reduce the risk of any potential contamination across sites, these pairs will form the 3 clusters which will be the unit of randomization as illustrated in figure 3. Long-term patient outcome data (12 months post-discharge) will be collected by participating physiotherapists by telephone contact.

Figure 3: The stepped-wedge design for the quantitative component of the study

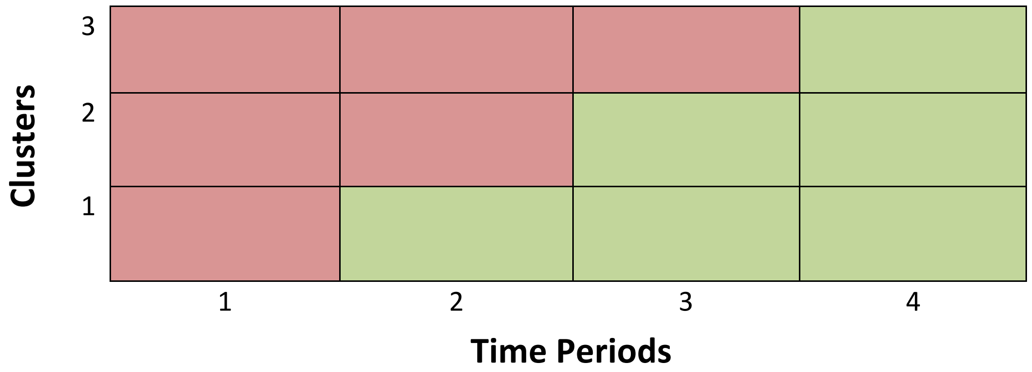

Methods - Interventions

Intervention

The first step (time point) corresponds to a baseline measurement where none of the clusters receive the intervention, which is usual practice for this design (Hussey and Hughes, 2007). At each subsequent step a cluster of participating physiotherapists (a department of the MSK outpatient physiotherapy service) will cross over from control to receive the intervention - a 150 hour clinical mentorship programme, aimed at facilitating clinical reasoning. This will be delivered to participating physiotherapists by mentors who are MACP members, having qualified at MSc / PgD level from a higher education establishment, and who have experience in delivering this form of mentorship at post-graduate level. The intervention will be delivered at the start of the time period, to allow for consolidation and application of the programme, before data collection occurs at the end of the time period. This intervention is selected on the basis that the use of mentorship in the context of clinical placement is established practice in Master's programmes seeking to develop expert practice and has strong support from qualitative literature as outlined earlier. The intervention will take place in the usual clinical context of the participating physiotherapists (as is current practice), consisting of the mentors observing the participating physiotherapists assessing and treating new and follow-up patients, discussing and facilitating clinical reasoning processes immediately after the patients have left. While this programme needs to be fluid enough to address identified learning needs and errors in clinical reasoning, a framework on which to hang discussion, feedback and reflection sessions will help to standardize the intervention to a point; the dialectical clinical reasoning model of Jones and Edwards, 2007 (see figure 4 below) will be implemented to this end. This model has been selected due to its flexibility for management and assessment paradigms, its adaptability to patient situations and presentations, and its underpinning research (Edwards et al., 2004, Edwards et al., 2006, Edwards and Jones, 2007). Failure of participants to receive the intervention will be reported, as well as the reasons for such failure.

Control

During the control steps of the study, participants will receive their usual training allocation. Usual training for staff in the health board involves monthly in-service training on current evidence applied to physiotherapy practice (4 hours per month), weekly technique sessions on the technical & practical skills of physiotherapy practice (30 minutes per week), as well as monthly mentoring sessions observing clinical reasoning (1½ hours per month). Should any of the physiotherapists not receive any or all of the control training, this will be reported, and the reasons why will be discussed.

This choice of control is consistent with other studies into educational interventions using crossover methodologies (Bye et al., 2009, Celebi et al., 2009, Cook et al., 2009, Cook et al., 2006, Murray et al., 1997a, Koles et al., 2005, Lam et al., 2004, Rawlins et al., 2009) where current or traditional practice is used to compare with new or proposed interventions on the basis that the comparison group needs to be plausible and fair (Murray et al., 1997b). Comparisons with "non- intervention" controls are strongly argued against (Cook et al., 2006) on the basis that such studies fail to inform the education community on the issue of selecting the most effective methods from multiple available options; the priority for study is not *whether* students and clinicians can learn using a designated method or combination of methods, but rather how *best* to facilitate learning.

Should any physiotherapists fail to complete the intervention or control elements of the study, this will be reported, as well as the reasons for such failure.

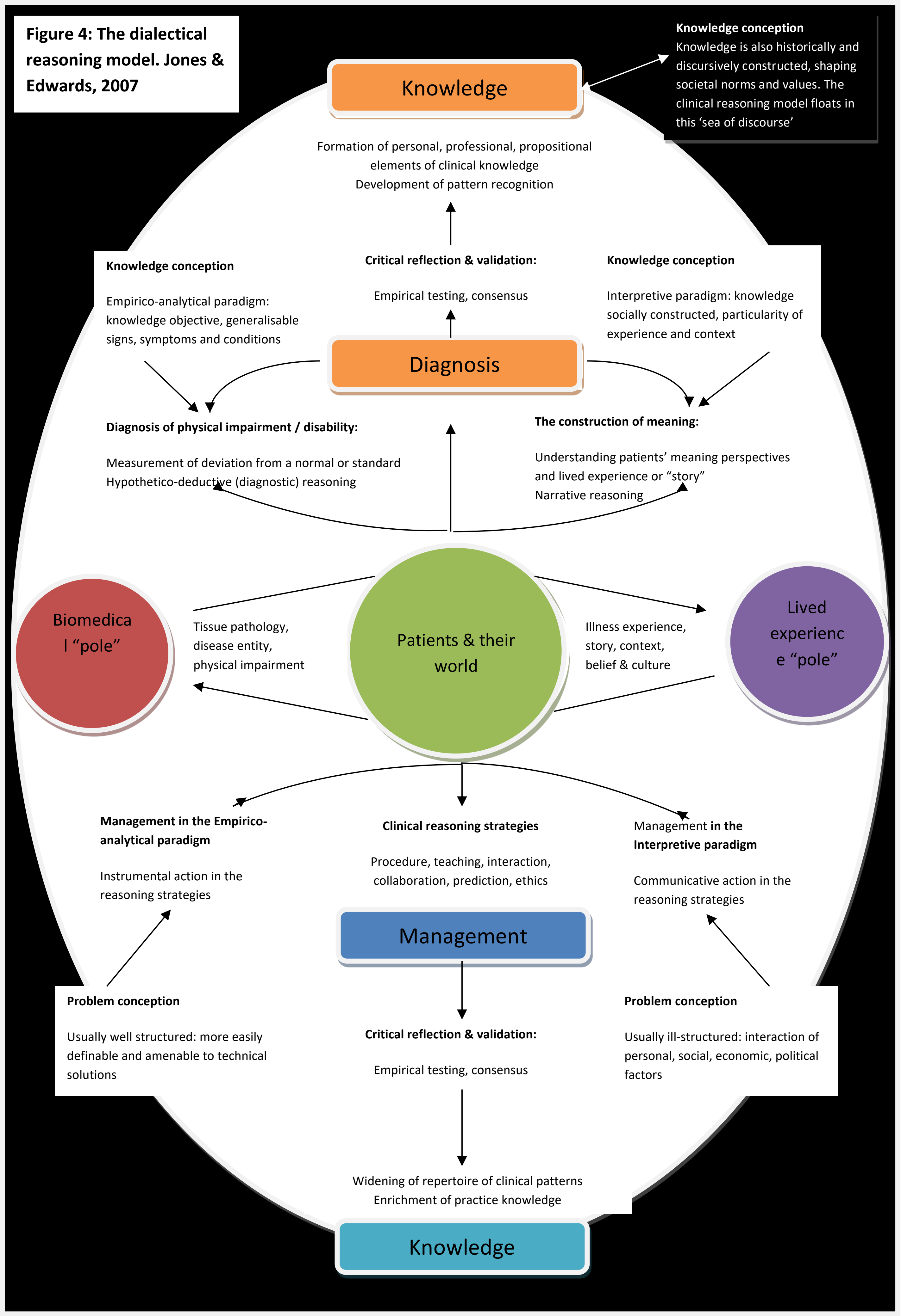
Figure 4: The dialectical reasoning model. Jones & Edwards, 2007
Knowledge
Knowledge conception
Knowledge is also historically and discursively constructed, shaping societal norms and values. The clinical reasoning model floats in this 'sea of discourse'
Formation of personal, professional, propositional elements of clinical knowledge
Development of pattern recognition
Knowledge conception
Empirico-analytical paradigm: knowledge objective, generalisable signs, symptoms and conditions
Critical reflection & validation:
Empirical testing, consensus
Knowledge conception
Interpretive paradigm: knowledge socially constructed, particularity of experience and context
Diagnosis
Diagnosis of physical impairment / disability:
Measurement of deviation from a normal or standard
Hypothetico-deductive (diagnostic) reasoning
The construction of meaning:
Understanding patients' meaning perspectives and lived experience or "story"
Narrative reasoning
Biomedical "pole"
Tissue pathology, disease entity, physical impairment
Patients & their world
Illness experience, story, context, belief & culture
Lived experience "pole"
Management in the Empirico-analytical paradigm
Instrumental action in the reasoning strategies
Clinical reasoning strategies
Procedure, teaching, interaction, collaboration, prediction, ethics
Management in the Interpretive paradigm
Communicative action in the reasoning strategies
Management
Problem conception
Usually well structured: more easily definable and amenable to technical solutions
Critical reflection & validation:
Empirical testing, consensus
Problem conception
Usually ill-structured: interaction of personal, social, economic, political factors
Widening of repertoire of clinical patterns
Enrichment of practice knowledge
Knowledge

Objectives

It is important to clarify the specific objectives and hypotheses for the quantitative component of the trial, and in particular - as this is a CRCT - whether these apply to the cluster level, to the individual level, or to both (Campbell, 2004). . Knowing the level of inference will subsequently aid interpretation of the statistical methods.

<u>Objectives</u>

It is the objective of the quantitative component of this study to

1. explore whether facilitation of clinical reasoning skills via a clinical mentoring programme delivered to clusters of physiotherapists (outpatient musculoskeletal physiotherapy departments) yields greater improvements in patient outcomes (measured by risk-adjusted patient outcomes measures from clusters of patients at each step) than those yielded with usual training
2. explore whether facilitation of clinical reasoning skills via a clinical mentoring programme relates to improved physiotherapist performance in clinical reasoning, and whether changes in performance correlate with improvements in patient outcomes

<u>Hypotheses</u>

H^1: There will be a significant difference in the risk-adjusted outcomes of patients whose physiotherapists have received training in clinical reasoning skills via a clinical mentoring programme when compared with those whose physiotherapists have received standard training.

H^0: There will be no difference in the risk-adjusted outcomes of patients whose physiotherapists have received training in clinical reasoning skills via a clinical mentoring programme and those whose physiotherapists have received standard training.

H^1: There will be a significant improvement in the performance of physiotherapists who have received training in clinical reasoning skills via a clinical mentoring programme when compared with their performance having received standard training.

H^0: There will be no improvement in the performance of physiotherapists who have received training in clinical reasoning skills via a clinical mentoring programme when compared with their performance having received standard training.

H^1: There will be a correlation between improvements in the performance of physiotherapists who have received training in clinical reasoning skills via a clinical mentoring programme and improvements in the clinical outcomes of their patients.

H^0: There will be no correlation between improvements in the performance of physiotherapists who have received training in clinical reasoning skills via a clinical mentoring programme and improvements in the clinical outcomes of their patients.

Outcomes

Clusters of patients will receive their usual physiotherapy treatment after the participating physiotherapists have received either intervention or control training. Outcome data from these clusters will be used to evaluate the effectiveness of the intervention and control training programmes. This data will be collected at the end of each time period to allow for the consolidation and application of the training (which will have been delivered at the start of the time period) to make an impact.

Patient Outcome Measures

In selecting appropriate outcome measures in physiotherapy outcomes research, Jette (Jette, 1993) suggests three important factors to consider - selection of dimensions to measure, psychometric properties and practicality. The selection of dimensions to measure are influenced by the World Health Organization (WHO)'s International Classification of Functioning, Disability and Health (ICF) framework (2001). The ICF is WHO's framework for measuring health and disability at both individual and population levels and was officially endorsed by all 191 WHO Member States in the Fifty-fourth World Health Assembly in 2001 for use in Member States as the international standard to describe and measure health and disability. The use of this framework has been strongly endorsed by multiple authors on the topic of outcome research in physiotherapy (Jette, 2010, Jette et al., 2008, Escorpizo et al., 2010, Mitchell, 2008, WCPT, 2011) on the basis of its common language in describing health states, its systematic and comprehensive classification, and the potential for outcomes measures built from the framework to allow comparison of health and health related states across patients, studies, countries, and clinical services. In particular, the ICF framework has been welcomed in its broadening of focus of outcomes away from the traditional biomedical research paradigm in physiotherapy, beyond mortality and disease, to focus using a more biopsychosocial paradigm on how people live with their conditions (Jette, 2006). In essence, the ICF identifies three levels of human functioning: at the level of body parts, the whole person, and the whole person in their complete environment. Each level, in turn, contains three domains of human function: body functions and structures, activities, and participation (Jette, 2009). The first of these domains - body functions and structures – are defined as such: "In the context of health experience, body functions are the physiological functions of body systems (including psychological functions). Body structures are anatomical parts of the body such as organs, limbs, and their components. Impairments are problems in body function or structure as a significant deviation or loss. Impairments within the ICF include deviations from generally accepted population standards in the biomedical status of the body and its function and can be temporary or permanent." (2001)

Historically, the measurement of impairments has been a key focus of physiotherapy treatment and research (Jette, 2005, Jette, 1995), but the limitations of impairment measurement have more recently been increasingly highlighted on the basis of lack of standardization of impairments and the poor reliability of common measures of impairments (Resnik and Dobrykowski, 2005). As a result, the activities and function domains have been met with enthusiasm within the physiotherapy research literature. The ICF defines an activity outcome as "the execution of a task or action by an individual." and a participation outcome as "involvement in life situations" (2001), the result of a complex relationship between a person, his or her health condition, and the person's environment (Jette et al., 2008). It is this emphasis on function that this study adopts and seeks to measure in its outcomes assessment, in order to address the needs already identified in the literature and research questions, as well as to address the research priorities identified in the following plea to physiotherapy researchers…

"There is a scarcity of available scientific evidence about the... health care needs of people with disability and about effective interventions for prevention, treatment, and rehabilitation. In addition to the lack of evidence about what works to improve activity and participation of those with disabilities, there also is little evidence about the costs incurred. We encourage scientists conducting research relevant to the nexus of physical therapy and disability to contribute to building this crucial knowledge base. What are the key physical therapy interventions that will improve levels of activity and participation for people with disabilities?" p. 324-5 (Jette and Latham, 2010).

Primary Outcome Measure

In seeking to select outcome measures that will capture data to appropriately answer the research questions, it is important to remember that while a single outcome measure is unlikely to encompass all that requires investigation, the use of multiple outcome measures increases the risk that a statistically significant result may be found by chance with each additional measure undertaken (Freemantle, 2001). This issue is simplified if researchers prospectively identify a primary outcome measure, the specification of which is a key feature of any randomised controlled trial (Freemantle, 2001). A broad range of patient outcomes will be used in order to ensure a comprehensive investigation of the impact of the interventions on patient outcome (please see table 1 for a summary of patient outcome data being collected), and the primary outcome measure will be indentified prospectively as suggested.

Health-related quality of life (HRQL) data have been recommended as valid, reliable and responsive outcomes measures for physiotherapists to assess treatment outcomes in patients with a wide range of health conditions (Jette, 1995, Jette, 1993, Resnik and Dobrykowski, 2005, Resnik and Dobrzykowski, 2003). HRQL measures can be generic, condition-specific, or patient-specific instruments, or a combination of instruments (Resnik and Dobrykowski, 2005). This research will use both generic and patient-specific HRQL instruments; condition specific measures will not be utilized due to the inability of different condition specific outcomes instruments to 'talk to each other' (Jette and Haley, 2005).

It is a patient-specific HRQL measure that will be selected as the primary outcome measure for several reasons. Firstly responsiveness - some studies suggest that patient specific instruments may be the most responsive of the HRQL tools, as they specifically addresses problems experienced by the individual patient (Resnik and Jensen, 2003, Stratford et al., 1995). The Patient Specific Functional Scale (PSFS) has shown itself to be more responsive than other established condition-specific measures (Pengel et al., 2004, Cleland et al., 2006, Young et al., 2010). It is the PSFS which will be used for the patients' most disabling functional problems on the basis of this responsiveness, and the three considerations of outcomes measures selection suggested by Jette (Jette, 1993) – psychometric properties, practicality and dimensions selected for measurement. The PSFS possesses established validity across body regions to give numerical rating scores that will be comparable (Brentnall and Sterling, 2007, Chatman et al., 1997, Cleland et al., 2006, Hefford et al., 2009, Hefford et al., 2008, Pietrobon et al., 2002, Sterling and Brentnall, 2007, Stewart et al., 2007, Westaway et al., 1998, Stratford et al., 1995) and the validity studies provide clear data for research purposes on the minimal detectable change (MDC) and the minimal clinically important difference (MCID)which are both reported to be three points for each identified activity and 2 points for aggregate activities (Stratford et al., 1995, Sterling and Brentnall, 2007). From a practical perspective, the PSFS is quick to apply in both the initial and subsequent assessments, is simple to administer and does not require any ranking procedures or software analysis, unlike many disability questionnaires (Brentnall and Sterling, 2007). Finally, the dimensions being measured are activity and participation; the PSFS has been deemed useful in redirecting questioning towards function and ability rather than pain and disability (Brentnall and Sterling, 2007), and in a study which explored how well the PSFS reflected the ICF (Fairbairn et al., 2010), 100% of the analysed items were mapped to the activity, participation, and impairment components of the ICF or overlapped between these components.

<u>Analysis</u>

The PSFS scores for every consecutive consenting patient of each participating physiotherapist will be analyzed for significant change:

- Total score = sum of the activity scores/number of activities.
- Minimum detectable change (90%CI) for average score = 2 points.
- Minimum detectable change (90%CI) for single activity score = 3 points.

Secondary Outcome Measures

1. Generic HRQL

Generic HRQL instruments are designed for broad use in a variety of patient populations, which is fitting for the broad variety of patients with different musculoskeletal conditions used in this study. The generic instrument selected to measure HRQL in this study is the Euroqol (EQ-5D) on the basis of its broad application to a wide range of health conditions and treatments, and its provision of a simple descriptive profile and single index value for health status (1990, Dolan, 1997, Fransen and Edmonds, 1999, Jenkinson et al., 1997, Polsky et al., 2001, Rabin and de Charro, 2001).

Analysis

The EQ-5D will be scored as per the standard UK algorithm.

2. Self Efficacy

Successful rehabilitation and expert practice may have additional aspects which are not fully captured by discharge functional or health related quality of life measures (Resnik, 2007). Changes in patient self-efficacy have been hypothesised as another valid outcome measure to use in identifying developing traits of expertise in physiotherapists (Resnik 2007) on the basis that one of the primary goals of rehabilitation that is widely recognized is the enhancement of patient's ability and confidence to manage their own health, particularly in chronic conditions where patients require problem-solving skills and management strategies to handle minor setbacks. Research suggests that activation is developmental and that individuals - on their journey to becoming effective self-managers - pass through different levels of activation (Hibbard et al., 2009). The Patient Activation Measure (PAM) will be used for this on the basis that it has undergone multiphase psychometric testing to confirm its validity and reliability (Agapova et al., 2010, Fowles et al., 2009, Green et al., 2010, Hibbard et al., 2009, Hibbard and Mahoney, 2010, Hibbard et al., 2007, Hibbard et al., 2005, Hibbard et al., 2004, Hibbard and Tusler, 2007) and on the basis that it maintains precision across different demographic and health status groups. The authors identify its use at the aggregate level to evaluate and compare the efficacy of interventions.

Analysis

The PAM score will be calculated using the standard scoring table, and patients will be classified into one of the four levels of activation outlined in the validity studies for the tool.

3. Patient Satisfaction

One of the key features of development in expertise in clinical reasoning is collaborative reasoning in both diagnosis and management (Edwards et al., 2004, Edwards and Jones, 2007, Jensen et al., 2008) where patient centred care is seen as integral to practice. Indeed, a move toward patient centered care has been recommended by the Department of Health (2007), and has seen the growth of interest in measurement of patient satisfaction as an important outcome measure in healthcare research (Casserley-Feeney et al., 2008). Levels of patient satisfaction would therefore be of interest and the MedRisk Instrument for Measuring Patient Satisfaction With Physical Therapy Care (MRPS) will be used on the basis of its strong psychometric properties assessed on a large, diverse population, its validation for use in an outpatient physiotherapy environment and its user-friendliness, being relatively short (12 items) (Beattie et al., 2005, Beattie et al., 2011, Beattie et al., 2007, Beattie et al., 2002). The availability of the psychometric data, plus the use of negative phrasing, multi-dimensional measurement and Likert scale, and the fact that it is self-completed by

the patient, satisfies the criteria raised by several authors for a suitable patient satisfaction questionnaire (Casserley-Feeney et al., 2008, Hudak and Wright, 2000, Sim and Wright, 2000, Sitzia, 1999).

Analysis

The MRPS will be scored using the database kindly provided by the MedRisk authors to analyze both internal and external factors affecting patient satisfaction.

Procedures

It is the *differences* in patient outcomes data that is being measured. Outcomes data will be collected from a cluster of patients being treated by each participating therapist at each stage of the stepped wedge design, giving 4 clusters of patient data per physiotherapist cluster for within group and between group comparison.

For each patient, outcome data will be collected at first and last appointment and at 12 months post discharge. The long term follow up is important in that – as Resnik (Resnik, 2007) points out - it is possible that aspects of physiotherapy intervention (for example, patient education) have health effects which are lifelong and therefore cannot be measured purely with discharge measurements of the patient's status. All patients treated sequentially by the therapist will complete the outcomes until a full data set is obtained.

Patient data loss from failure to complete treatment / DNA will be minimized by telephone contact, having consented patients to being contacted by the researchers by telephone as part of the study follow up. Patients refusing to complete data or withdrawing consent will be reported as will the reasons for failure to complete treatment and / or outcomes.

Strategies to Enhance Quality of Outcomes

Risk Adjustment

To enhance the quality of these outcomes, control of variables that could affect the dependent variables (patient outcomes) is essential before comparing patient outcomes are compared between clusters and within clusters. While no consensus exists for the optimal risk-adjustment method, an approach to risk adjustment has been previously used for outcomes research in physiotherapy (Jette and Jette, 1996; Resnik and Hart, 2003). First, using data from step 1 (before any therapists have commenced intervention) univariate analyses will be used to identify possible confounding variables among patient characteristics (from previous literature, age, severity, sex, onset of condition, number of surgeries for condition, reimbursement, exercise history and employment status).

Next, a general linear model (GLM) will be developed for the HRQL outcome measures—the PSFS and EQ-5D. Each patient characteristic found to be significant in the univariate analyses will be included in the models using a backward-deletion process. General linear models allow for simultaneous control of continuous and categorical variables in the risk-adjustment process. It is the risk-adjusted HRQL data that will be used for all data analysis to analyse the effect of the intervention.

Psychosocial Screening

In addition to risk adjustment, a formal assessment of levels of psychological distress and / or depression of patients will be made due to the fact that it is now widely accepted that such factors are strong predictors of outcome in musculoskeletal conditions (Main et al., 2007) and that pain is best viewed as a multi-dimensional phenomenon, comprising biological, psychological, social and existential elements (Westman et al., 2008). For the individual patient, persistent musculoskeletal pain has been shown to significantly affect the patient's quality of life with symptoms usually being continuous and disrupting a variety of functions (Costa Lda et al., 2011, Heymans et al., 2010, Lin et al., 2011). However, it must be remembered that not all

patients develop chronic problems – for example it has been shown that after an acute bout of back pain, only a small percentage (3–10%) of patients developed long-term work absence but this small number consumed approximately 75–85% of the available resources (Reid et al., 1997, Nachemson et al., 2000, Friedly et al., 2010).The Örebro Musculoskeletal Pain Questionnaire (ÖMPQ) will be used for this formal assessment on the basis of its predictive ability to identify patients who will have a poor outcome across a range of musculo-skeletal conditions – acute, subacute or chronic, and different body regions; any improvements (or lack of improvements) in the other outcome measures need to be cross-referenced with these profiles. (Brown, 2008, Dunstan et al., 2005, Gabel et al., 2010, Hockings et al., 2008, Johnston, 2009, Linton and Boersma, 2003, Maher and Grotle, 2009, Margison and French, 2007, Westman et al., 2008).

Analysis

The ÖMPQ will be scored and interpreted at the start of treatment as per the scoring instructions:

- For question 1, count the number of pain sites and multiply by two – this is the score (maximum score allowable is 10).
- For questions 2 and 3 the score is the number bracketed after the ticked box.
- For questions 4, 5, 6, 7, 9, 10, 11, 14, 15 and 16 the score is the number that has been circled.
- For questions 8, 12, 13, 17, 18, 19, 20 and 21 the score is 10 minus the number that has been circled.

A total score of 105 or above indicates persons who are at risk of disability or failure to return to work.

Therapist Outcome Measures

Therapist performance will be assessed by an independent assessor, using the criteria currently used by tutors at the University of Birmingham to assess improvements in performance of physiotherapists as part of the summative assessment of the Evidence Based Clinical Practice module of their MSc in Advancing Practice and Advanced Manipulative Physiotherapy programmes (see Appendix D). This will take place at all 3 data collection points (baseline and post each intervention). As these courses - and this module - has been approved by the MACP and its international umbrella organization IFOMPT, the use of an agreed set of marking criteria that distinguishes advanced practice in manipulative physiotherapy for the outcome of the practice based experience, is required for continued accreditation by the MACP. The module's marking criteria, therefore, contain these agreed criteria. On a practical level, accreditation specifies that the summative assessment encompasses the evaluation of a minimum of one new and one follow-up patient (IFOMPT, 2008). This study will utilize the same basis for assessment of therapist performance, where the therapist will be observed by an independent (non-health board employee), experienced (familiar with - and experienced in - using the University of Birmingham criteria) assessor who will observe the therapist with a new patient and follow up patient.

In addition to being informed by nationally applied international standards of educational practice, the University of Birmingham criteria (in preference to other University course criteria) are supported by empirical data (Rushton and Lindsay, 2010, Rushton and Lindsay, 2008). One study (Rushton and Lindsay, 2008) explored Masters courses in the United Kingdom assessing clinical practice (nursing/midwifery, physiotherapy, radiography, and inter-professional) using a modified Delphi-technique to define the behaviours indicative of the construct of masters level clinical practice. There was a very good response rate by the course tutors for the Delphi study (79.1%) and the level of consensus for the behaviours identified for the first round was good (with the three most commonly occurring behaviours being provided by more than 60% of the participants, and a further five behaviours provided by more than 50%). The second round required ranking of the importance of each behaviour identified from themes from first round data by the

participants; again there were high levels of agreement from participants for the importance of most behaviours (indicated by high mean scores and low standard deviations and values of the coefficient of variation). The third round required participants to rank the importance of the different behaviours to Masters level clinical practice. Incidentally, but significantly for this research, consensus across all participants identified a high level of clinical reasoning as having the greatest importance to the construct. There was statistically significant agreement across all participants and within both the physiotherapy group and the radiography group, but rankings differed between professions and participants reported that they would rank differently according to speciality within a profession. The lowest ranked behaviour was removed due to the low level of consensus. The resulting 20 behaviours demonstrated good measurement validity and external validity. It is these behaviours from this study that have produced the construct for the marking criteria in use for the University of Birmingham's Masters Courses seeking to develop clinical expertise.

The same authors (Rushton and Lindsay, 2010) also explored the construct of masters level manipulative physiotherapy clinical practice in its real life context, using an exploratory case study of the University of Birmingham Masters course in Manipulative Therapy. Semi-structured interviews with thirteen students and eleven clinical tutors formed part of the research, and implicit use of the construct were explored by participant observation of seven clinical examinations. Triangulation of data across methods, and convergence with the Delphi study discussed previously (Rushton and Lindsay, 2008)informed the conclusions. Again, the most important behaviour associated with the construct for manipulative physiotherapy was identified as a high level of clinical reasoning and validation of the construct was supported by convergence of data with the Delphi study.

In other studies exploring the effect of educational interventions using crossover methodology, (Cook et al., 2007, Murray et al., 1997a) the point is made that assessment needs to be appropriate – specifically comparisons of students using pre and post intervention testing, a design which has been widely accepted and favoured as a method of assessing the efficacy of skills teaching. While the primary outcome measure for this study - patient outcome measurement - appears to be a new venture for educational interventions, the undertaking of pre-and post-exposure assessment using a widely accepted and nationally and internationally recognized method for assessment of clinical performance satisfies this demand.

All of this serves to underpin the assessment of physiotherapist performance in this study by utilising the University of Birmingham construct for the assessment of improvements in performance of physiotherapists, by observing each therapist with one new patient and one returning patient at three data collection points Assessment of participants at step 1 will give baseline data on performance levels; assessment of participants during step 2 or 3 will ensure that the independent assessor will not know if therapists have received training or not; assessment during step 4 will give post-intervention measures for all participants.

Cost-Effectiveness Measures

To establish the cost-effectiveness of the mentoring package, the following will be assessed:

- Referral on for secondary care opinion or intervention, or for diagnostic imaging.
- Use of prescription medication.
- Costs of physiotherapy intervention (number and type of attendances)
- Return to work
- Costs associated with the training of physiotherapists

The rationale and description of this element of the study is developed and explored in the economic evaluation section along with details of analysis.

The quantitative data collection for this study is summarized below in table 1.

Table 1: Summary of Quantitative Data Collection

Dimension measured:	Rationale for dimension:	Measurement tools:	Rationale for tool:
Patient outcome:<br><br>•Function<br>•Health-related quality of life<br>•Patient satisfaction<br>•Patient self-efficacy	The lack of research examining the link between therapist training and patient outcomes, despite multiple calls for such research to be undertaken as a priority.	Patient Specific Functional Scale (PSFS)	1. The provision of numerical rating scores that will be comparable across body regions.<br>2. Focus on function in line with WHO ICF.<br>3. Responsiveness of patient-specific tools.
		EuroQuol (EQ-5D)	1. Broad application to a wide range of health conditions and treatments.<br>2. Provision of a simple descriptive profile and single index value for health status, allowing for comparison across clinical presentations, and cost utility analysis.
		MedRisk Instrument for Measuring Patient Satisfaction With Physical Therapy Care (MRPS)	1. Strong psychometric properties assessed on a large, diverse population.<br>2. Validation for use in an outpatient physiotherapy environment.<br>3. User-friendliness.<br>4. Satisfaction of criteria recommended by multiple authors for an appropriate patient satisfaction questionnaire (use of negative phrasing, multi-dimensional measurement and Likert scale, and self-completion by the patient).
		Patient Activation Measure (PAM)	1. Multiphase psychometric testing to confirm its validity and reliability.<br>2. Maintains precision across different demographic and health status groups.<br>3. Identified for use at the aggregate level to evaluate and compare the efficacy of interventions.
Patient distress and anxiety	To cross reference and enhance primary and secondary outcomes	Örebro Musculoskeletal Pain Questionnaire (ÖMPQ)	1. Predictive ability to identify patients who will have a poor outcome across acute, sub-acute or chronic musculo-skeletal conditions.<br>2. Predictive ability to identify patients who will have a poor outcome across different body regions.
Patient demographic and healthcare utilisation data	To enable adequate risk adjustment of primary and outcomes and cost effectiveness analysis	Questionnaire	Items previously shown in other studies to be useful for risk adjustment and cost effectiveness are included.
Therapist Performance	Previous educational research has measured performance; assessment will allow for triangulation of patient outcomes data with that of therapist performance.	University course M level descriptors for assessing therapist performance on clinical placement	1. Accepted and established form of assessing performance on M level courses where clinical placements are used.<br>2. Supporting research for the construct.

Sample size

The primary outcome measure, the PSFS, shows that for any individual patient, the minimal detectable change (90%CI) for an aggregate of 3 or more activities is 2 points; the minimum detectable change (90%CI) for a single activity is 3 points. In this study, we are looking for a mean difference of 1 point greater improvement in PSFS scores of patients whose physiotherapists who have received the intervention compared with the PSFS scores of patients whose physiotherapists have not yet received the training, as well as a greater proportion of patients achieving the level of minimal detectable difference. The following data values were used for the power calculation: mean changes on the PSFS from usual training to the new training of 1 point, and a Standard Deviation within each group of 2. The standard deviation was on the basis of PSFS data collected from an audit of 191 patients who had received treatment at the University Health

Board physiotherapy department, which is a slightly more conservative estimate than standard deviation values of PSFS outcomes from published studies (Hefford et al., 2009, Young et al., 2010, Costa et al., 2009).

A paper published on the design and analysis of stepped wedge designs (Hussey and Hughes, 2007) investigated the statistical characteristics of the stepped wedge design for CRCTs, and through the use of theoretical calculations and simulation the authors outline a procedure for computing power in stepped wedge trials. Using this procedure, a power calculation was carried out by Dr. Alan Watkins, Senior Lecturer in Statistics, Swansea University in discussion with the rest of the research team. The power calculation is summarised below:

Power calculation: summary

Following the notation of Hussey & Hughes (Hussey and Hughes, 2007), and defining

- I to be the number of physios (the maximum available would be 18; assuming recruitment of two-thirds of the maximum to be a realistic prospect, so we are primarily interested in I = 12)
- T to be the number of time points (4 time points are required to allow baseline measures and for 3 clusters to cross over from control to intervention, so we are primarily interested in T = 4)
- N to be the number of observations per physiotherapist per time point.

The model is

$$Y_{ijk} = \mu + \alpha_i + \beta_j + \theta X_{ij} + \varepsilon_{ijk}$$

where Y_{ijk} is the kth observation at time *j* for physiotherapist *i*, and we assume that there is a random physiotherapist effect, so

$$\alpha_i \sim N\ (0,\ \tau^2)$$

and some random variation across observations, so

$$\varepsilon_{ijk} \sim N\ (0,\ \sigma_\varepsilon^2)$$

with independence everywhere. The X_{ij} are indicator variates, with value 0 before the intervention, and the value 1 thereafter. The range of indices are *i* = 1, ... , *I*; *j* = 1, ... , *T* and *k* = 1, ... , *N*. We take β_T = 0 in order to avoid identifiably problems, and wish to consider ϑ, the difference in means in the control and intervention group. The variance of $\hat{\theta}$ is given in equation (8) in Hussey & Hughes (Hussey and Hughes, 2007), and its calculation requires the specification of σ_ε^2 and τ^2, with *N* appearing through

$$\sigma^2 = \frac{\sigma_\varepsilon^2}{N}$$

Omitting some algebra, statistical power, based on the usual 95% confidence level, is defined by

$$1 + \Phi\ (-1.96 - \Delta) - \Phi\ (1.96 - \Delta)$$

where

$$\Delta = \frac{\theta_A}{Var\ \hat{\theta}}$$

and Φ (•) is the usual cumulative distribution function for the standard Normal distribution, and is implemented in EXCEL as =NORMSDISTO.

Base case details

- Here, we take $\theta = 1$ (that is, assume that the average effect of the intervention is 1), and (based on calculations on previous data) set $\sigma_\varepsilon = 2$**.** The remaining calculations are laid out in the attached spreadsheet, where, for illustration, if we take $N = 9, \tau = 1$ then

$$Var\left(\hat{\theta}\right) = 0.12576 \ldots,$$

and

$$\Delta = 2.819574$$

so that the power is

$$1 + \Phi(-1.96 - 2.8196) - \Phi(-1.96 - 2.8196) \simeq 1 - \Phi(0.8596) \simeq 0.805$$

- This implies that, in order to achieve a power of 0.8, we need a total of 4 × 12 × 9 = 432 observations.

Other cases

It should be strongly emphasized that the power calculations are sensitive (in varying degrees) to changes in $\theta, \sigma_\varepsilon$ and τ. The spreadsheet in appendix E summarizes the power for other combinations of τ, N, and these are displayed in the graph in the spreadsheet as a function of N for the same $\theta, \sigma_\varepsilon$. The chosen values of τ may also be summarized via the intra-class correlation

$$p = \frac{\tau^2}{\sigma_\varepsilon^2 + \tau^2}$$

For most cases of τ yielding p in the range [0, 0. 5], the required power of 0.8 is achieved with between 6 and 10 observations per physiotherapist per time point.

Randomisation

The stepped wedge design is a one-way crossover design in which different clusters cross over (switch treatments) at different time points (Mdege et al., 2011). In addition, the clusters cross over in one direction only—typically, from control to intervention. The first time point usually corresponds to a baseline measurement where none of the clusters receive the intervention of interest. At subsequent time points, clusters initiate the intervention of interest and the response to the intervention is measured. More than one cluster may start the intervention at a time point, but the time at which a cluster begins the intervention is randomized (Brown and Lilford, 2006, Hussey and Hughes, 2007).

Enrollment: participating physiotherapists will be recruited by the lead researcher by invitation on the basis of inclusion criteria. They will be approached by the lead researcher, and issued with participant information sheet version 2.0 (see appendix C). The lead researcher will also obtain written consent with participant consent form version 2.0 (see appendix C). During data collection periods, patients will be recruited by their physiotherapist, and issued with patient information sheet version 1.0 (see appendix B). The physiotherapist will also obtain written consent with patient consent form version 1.0 (see appendix B).

Sequence generation: once consented by the lead researcher, participating therapists will be directed to the clinical mentors. The clinical mentors will then randomly allocate the clusters of physiotherapists (clustering by site) to the sequence of intervention (to receive the intervention in time period 2, 3 or 4), by computer programme (Randomization.com, 2008).

Allocation concealment: The sequence generation will be performed by the allocated mentor, in order to ensure that this sequence allocation will be adequately concealed from the lead researcher, who will remain blinded to the sequence until after the data collection and analysis period is completed. These measures for blinding, sequence generation and allocation concealment are implemented to reduce the risk of bias, as highlighted by the Cochrane risk of bias tool (Armijo-Olivo et al., 2010, Lundh and Gotzsche, 2008).

Blinding (Masking)

As with all other evaluations, masking patients, caregivers and observers, as well as those undertaking the statistical analysis is important in minimising information bias (Schulz et al., 2002, Schulz and Grimes, 2002). Due to the fact that in stepped wedge trials the timing of intervention rollout is the unit of randomization, the participating physiotherapists and intervention implementers (clinical mentors) are aware of which cluster is receiving the intervention (Brown et al., 2008, Mdege et al., 2011). However, it is possible to blind the outcome assessors, in this case the lead researcher (who will be unaware of the sequence allocation when analysing outcome data) and the independent observer of physiotherapist performance. This blinding of outcome assessors and use of sequence generation and allocation concealment is essential to enhance internal validity (Brown et al., 2008). Patients submitting their outcomes will also be unaware of whether or not their allocated physiotherapist has received his/her intervention.

Statistical methods

The analysis of stepped wedge trials has been clearly outlined in a published paper (Hussey and Hughes, 2007) and recommends processes for analysis of cluster-level means and individual level analysis in scenarios where cluster sizes are equal or unequal, and where variance is known or unknown. Processes for between cluster and within cluster analyses are outlined in order to avoid confounding the treatment effect with changes over time; these processes will form the basis for the statistical analysis for the study. If no temporal effects are found influencing the outcome, then a within-cluster analysis can be used to estimate the treatment effect. Statistical analysis will involve a classical ANOVA (so observations belong to one of two groups, each assumed to be independent of the others), supplemented by a random intercept which will reflect systematic differences across different clusters. Similar statistical methods have been used in other published stepped wedge trials (Schnelle et al., 1992, Bailey and Archer, 2004, Mdege et al., 2011).

Examples of use of this type of design

A systematic review of stepped wedge trial designs in 2006 (Brown and Lilford, 2006) yielded 12 published trials and protocols evaluating a wide range of interventions, across different diseases in different settings. It was noted that the stepped wedge design was used most frequently in developing countries, particularly for evaluating interventions concerned with HIV. A subsequent systematic review (Mdege et al., 2011) included 25 studies, and concluded that the stepped wedge trial design has been mainly been utilised to evaluate interventions during routine implementation. In particular where interventions have been shown to be effective in more controlled research settings, or where evidence of effectiveness is lacking but there is a strong belief that they will do more good than harm. This latter scenario is closest to the rationale for this research project in that there is a strong belief that this intervention of clinical mentoring which is established both nationally and internationally by Master's programmes to develop physiotherapist expertise, should benefit patients who receive treatment from physiotherapists who have received such mentoring. Of particular interest from this last review are three studies which have used stepped wedge trials to evaluate educational interventions (Ni Mhurchu et al., 2010, Bailet et al., 2009, Howlin et al., 2007) underlining its usefulness for this research project.

There are also examples of the usage of this type of design in researching educational interventions with parallel crossover designs. From educating gait patterns in patients with Parkinson's disease (Nieuwboer et al., 2007) to the education of lay people in CPR techniques (Rawlins et al., 2009) and – most relevant to this research - to clinical skill education; crossover study designs are utilized to research educational interventions in developing technical surgical skills (Cho et al., 2008, Fikkers et al., 2004) and knowledge and clinical reasoning in both undergraduate and postgraduate clinicians (Bye et al., 2009, Cook et al., 2008, Lam et al., 2004, Murray et al., 1997a, Koles et al., 2005, Celebi et al., 2009, Cook et al., 2007, Cook et al., 2009, Cook et al., 2006). These are of interest for the justifications given for the selection of this methodological choice by the authors of studies in medical education: the ability to ensure that all participants receive all training (Bye et al., 2009, Celebi et al., 2009, Rawlins et al., 2009, Murray et al., 1997a) and the ability to control for confounding variables (Cook et al., 2005, Cook et al., 2008, Rawlins et al., 2009, Koles et al., 2005, Murray et al., 1997b) are put forward to support the use of crossover study designs in medical education research.

Challenges in using this design and how they will be addressed

The main challenge associated with the design, conduct, analysis, and interpretation of CRCTs compared with individually randomized trials, is that two different units of measurement—the cluster and the patient—are used (Campbell, 2004). To address this, each will need to be reported carefully.

There are also several challenges raised by the stepped-wedge design. The first group of challenges are statistical. The unidirectional aspect of the crossover in the stepped-wedge design complicates the analysis because the treatment effect can no longer be estimated exclusively from within-cluster comparisons; within-cluster analysis has the potential for bias where the estimated treatment effect may be biased due to failure to model time effects (Hussey and Hughes, 2007). This will be addressed by performing both within-cluster and between-cluster analysis in order to estimate the treatment effect. This approach avoids confounding the treatment effect with changes over time; however, if there are no temporal effects on the outcome then within-cluster analyses can be used to estimate the treatment effect. In addition, time effects will be included in the model for analysis as suggested in the literature (Hughes, 2007, Hussey and Hughes, 2007, Brown et al., 2008).

Another challenge is the possibility that the full effect of the intervention may not be realized until several time intervals following implementation, for example, the intervention being 50% effective after the first time interval, 75% effective after the second time interval and 100% effective after the third time interval (Hussey and Hughes, 2007). Delayed effects from the intensive mentoring for clinical reasoning facilitation are possible, with physiotherapists requiring time to consolidate and make practice changes. The overall effect of these delayed effects would be to reduce power. Such delays can be modelled into the power calculations, and power can be recovered in part by adding additional measurement periods to the end of the trial. However, it is preferable to address this issue by making the time intervals sufficiently long to see the effects of the intervention realized in a single interval (Hughes, 2007, Hussey and Hughes, 2007). To address this, the clinicians who will act as clinical mentors in this study were asked to reflect on their own experience following mentoring programmes from their Masters courses, and estimate what time period they thought would be sufficient to consolidate knowledge and change practice. All responses estimated between 6-12 months. To this end, each time period will be 12 months long, with the intervention delivered at the beginning of the time period and the data collection at the end of the period to allow for the full effects of the intervention to make an impact.

One group of authors (Brown et al., 2008) highlight the amount of data collection required as the main disadvantage of the stepped wedge design, which could make the cost of using the design prohibitive, unless

the study can use routine, or other easily collected data. This study aims to collect patient outcome data at first and final appointments which is part of routine practice in the physiotherapy service at the Cardiff and Vale University Health Board. An additional collection of these outcomes will take place 12 months post discharge by telephone. Demographic and cost-effectiveness data will be collected at the same time in a single compiled questionnaire. This would certainly fit the description of routine, easily collected data.

Variables

For sake of clarity in the methodological design of this study, the variables require explicit definition. The independent variable will be defined as facilitation in clinical reasoning via a clinical mentoring programme. The dependent variables will be defined as patient outcomes and therapist performance. The control variables will be defined as and usual therapist training, patient age, patient condition, patient prognostic barriers, therapist participation, and organizational context.

Validity approaches in quantitative methods: Identification of threats to validity and responses

Creswell (2009) identifies common threats to both internal and external validity. Of particular relevance to this study, internal threats could be due to history, selection, compensatory processes, testing or instrumentation. History threats can be due to external events that can occur during the time that elapses in an experiment, which have an impact on the outcome besides the experimental treatment. To counter this, the research design uses participants from a single profession (physiotherapy), working in a similar environment (musculo-skeletal outpatient departments) in the same NHS health board (Cardiff and Vale); this means that professionally and organizationally the participants should experience the same external events. Selection threats can be due to participants being selected who have certain attributes that predispose them to have better outcomes (e.g., he or she is already more advanced in clinical reasoning, or is brighter or more likely to respond to this form of educational intervention), thus increasing the risk of bias as highlighted by the Cochrane risk of bias tool (Armijo-Olivo et al., 2010, Lundh and Gotzsche, 2008). To counter this, the study has utilised a stepped wedge design (using a one way crossover), so that each participant acts as his / her own control. This does raise the interesting question of different participants' learning styles having a hidden but profound effect on learning effectiveness, and this will be explored using a learning styles questionnaire (Honey and Mumford, 2000a, Honey and Mumford, 2000b). Compensatory threats occur when benefits provided in the experimental group leave participants in the control group resentful, demoralized or feeling devalued, skewing results. Again, the stepped wedge design of this trial counters this threat, with each participant acting as their own control, meaning benefits are provided to both groups, and the fact that during the control phase of this process usual training is being received. Testing threats occur when participants become familiar with the outcome measure being used and remember their responses for later testing. This is countered by the primary outcome measures being *patient* completed outcome measures, and the long-term follow up meaning that recall of previous responses is highly improbable. Instrument threats occur with changes in outcome measures between a pre-test and post-test; in this case, a broad range of outcomes are used to make any desire for changes in outcome measures unnecessary.

External threats to validity in this study could occur from the narrow characteristics of participants in the experiment, care must be taken not to generalize to individuals who do not have the characteristics of participants. Therefore, while methods and results may be of interest to - and have relevance for - other allied health professions, additional experiments would need to be performed to ascertain whether similar results are elicited. Likewise, due to the narrow characteristics of the setting of participants in this

experiment (musculo-skeletal outpatients), care must be taken not to generalize to individuals in other settings (inpatient settings) without further experimentation.

Other threats to validity could involve statistical conclusion threats which occur from inadequate statistical power or violation of statistical assumptions leading the researcher to draw inaccurate inferences or conclusions from the data. In addition, construct validity can be threatened when the researcher uses inadequate definitions and measures of variables. The measures of variables have already been outlined as appropriate, for the variables that have been accurately defined, and the research study has been appropriately powered.

Conclusions and applications

To conclude the discussion of all of these issues, the selection of the stepped-wedge design would appear to be the most appropriate to address the research problem and research questions. The alternatives of parallel designs (crossover or non-crossover) would be limited by issues relating to balancing covariates, sample size, logistics of intervention delivery and potential contamination. The arguments presented for the advantages of crossover methodology used elsewhere in healthcare education are compelling, and the use of stepped-wedge designs in evaluating educational interventions reinforce these arguments. By employing a longitudinal study design, improvements are expected to be made over time as skills are learned, applied and refined; the stepped-wedge design allows for the investigation of time as a variable in the final analysis. The timings of data collection are important; early implementation of the intervention within the time period, data collection near the end of the time period, and sufficiently long time periods to allow for delays in effects from the intensive mentoring for clinical reasoning facilitation, due to time required to consolidate and make practice changes. The use of usual training as control is also appropriate; a non intervention control in this instance would be ethically unacceptable – withdrawing training for staff responsible for patient care - as well as unhelpful for cost-effectiveness analysis where comparison should be made as closely as possible with usual practice (Gray et al., 2010). The assessment issue of recognized assessment tools pre- and post-exposure raised by Murray and colleagues (Murray et al., 1997a) is also addressed.

The overall structure of the quantitative component of the project is outlined and summarized in the consort diagram in figure 5.

Figure 5: CONSORT 2010 Flow Diagram of Quantitative Component of Trial

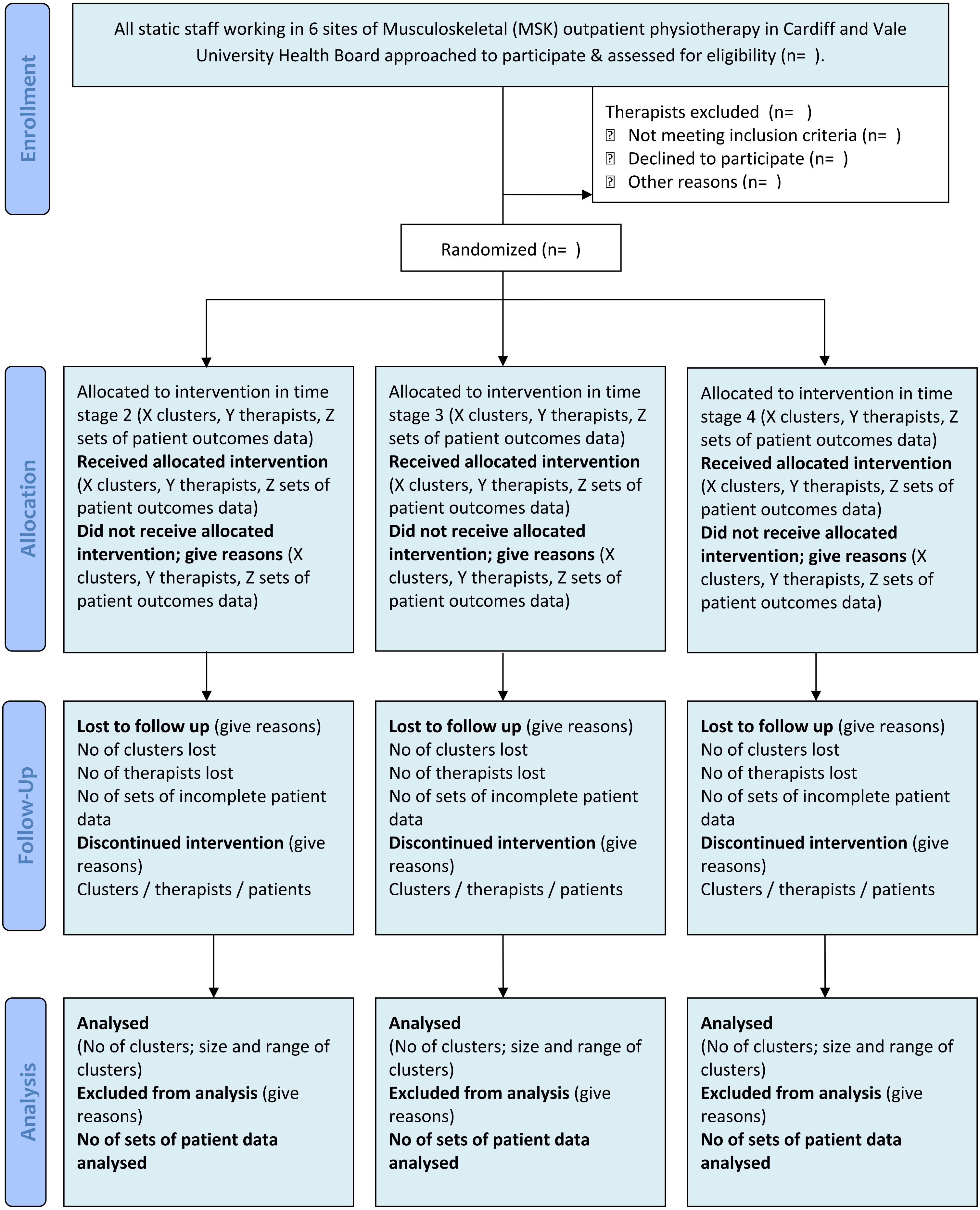

Qualitative Component

Cresswell (2009) outlines characteristics of qualitative research, many of which lend themselves to the clinical context of this study. Qualitative research tends to involve data collection in the natural setting – that is, in the field, at the site where participants experience the issue or problem under study; in this case, in the clinical context of the participants, with the researcher as key instrument collecting multiple sources of data. This allows for collaboration with the participants interactively during the data analysis, so that the participants have a chance to shape the themes or abstractions that emerge from the process, thus allowing the researcher to keep a focus on learning the meaning that the participants hold about the problem or issue, not the meaning that the researcher brings to the research. Qualitative data from mentors and participating therapists will allow for a more holistic approach to what has been identified as a complex issue under study (Kalet et al., 2010). In reporting multiple perspectives, it is hoped that the many factors involved in the process of facilitating clinical reasoning in order to achieve improved clinical outcomes will be identified, allowing for a broader discussion on the picture that emerges.

Type and definition of design used

Creswell (2009) outlines four main categories of qualitative data collection procedures: collecting information through unstructured or semi structured observations and interviews, documents, and audio-visual materials. Semi-structured interviews and observations will be used for this study, which will be defined and reasons for their selection will be outlined below.

Semi-structured interviews

Murphy et al (Murphy et al., 1998) identify the use of interviews in scenarios where researchers seek to discover what people think about the world they live in, how they evaluate their experiences within it and why they behave as they do, interpreting the choice to use interviews to collect data as, “If you want to understand what people do, believe and think, ask them” (p112 Murphy et al., 1998). Qualitative interviews allow relatively rapid data collection based on what interviewees say about their beliefs and attitudes, action and behaviour (Pope and Mays, 2006). Miles and Huberman (1984), Noaks and Wincup (2004) and Silverman (2010) strongly suggest that researchers do not present their main research question directly to the respondents themselves on the basis that this can affect their responses, and can lead to simply reporting back what respondents have told you instead of undertaking and reporting careful data analysis. Bearing this in mind, the general aim of the interviews in this study is to encourage the participants to speak personally and at length about their professional development as physiotherapists, while at the same time covering the issues which are of interest in answering the research questions – namely, to explore the participants’ and mentors’ experiences about the interventions in the study - mentoring and training, and their evaluation of both. As a result, there will need to be a balance maintained between what is interesting to the researcher and what is interesting to the participant (Silverman, 2006). To meet this aim, face-to-face interviews with participants and mentors will be conducted at the end of the intervention period. The closeness of the lead researcher to the clinic sites will allow for this rather than needing to interview participants by telephone. The subsequent questions that arise are what type of interview to employ and whether to perform interviews individually or to utilize focus groups to gain these reports from participants?

Several authors have sought to discuss the distinction and differences between structured and unstructured interviews (Burgess, 1982b; Habermann-Little, 1991; Fontana and Frey, 1994). However, some authors (Murphy et al., 1998, Britten, 2006) challenge the term ‘unstructured’ as misleading due to the impossibility of conceiving an interview which is totally without structure, and acknowledging that without structure

there would be no guarantee that data collection would be relevant to the research question. Instead they concur with Richardson and colleagues (Richardson et al., 1965) to suggest that it is more accurate to consider interviews as ranged across continuum of standardization. Britten (Britten, 2006) divides interviews into structured, semi-structured and depth. This division describes structured interviews as being performed by interviewers administering questionnaires andasking questions in a standardized manner; semi-structured interviews using open-ended questions based on a loose structure which defines the area to be explored, but which allows deviation to further explore responses and ideas; depth interviews are less structured again, with questions based on what the interviewee says, and only one or two issues explored in greater depth. This study will use semi-structured interviews, with structured interviews being too rigid and imposing to meet the aims of the exploratory nature of this element of the research in discovering the interviewees' own perspectives and frameworks of meaning (Britten, 2006) and depth interviews lacking the necessary breadth to explore the issues identified from previous qualitative research (Jensen et al., 2007b, Resnik, 2007).

Kitzinger (Kitzinger, 1995, Kitzinger, 2006) suggests the use of focus groups for studying cultural values such as work place cultures – such as the training culture in the health board under study in this research. She suggests this on the basis that focus group interviews tap into interpersonal communication to highlight cultural values or group norms. While Morgan (Morgan, 2010) also acknowledges such strengths, he also highlights the difficulties and challenges of such group dynamics; for example the voicing of group norms may silence individual objections and voices of dissent, and also the fact that other research participants are present means that the confidentiality of the research is compromised. This is of particular concern in the context of this research where great care is being taken to ensure that individual therapists' confidentiality is safeguarded both for their outcomes and their interview data. For example, therapists will be encouraged to discuss their relationship with their mentor; this is potentially sensitive data requiring absolute confidentiality.

There is a pragmatic argument for using focus group interviews over individual interviews. Citing one of the few studies that compare output of individual interviews and focus group interviews (in the generation of ideas) by Fern (Fern, 1982), - which showed that focus groups produced fewer, lower quality ideas when compared with individual interviews- Morgan (Morgan, 1996) concedes that it does not support the notion that focus groups have a synergy leading to greater depth and breadth of data, but suggests that focus groups could be selected due to their efficiency of output – the same number of ideas from Fern's results being produced from two focus groups as in 10 individual interviews. While - in this study - focus group interviews would allow for collection of this data at fewer visits, the staggered start and finish times of participants in the process as dictated by the crossover design would mean long time delays for some participants before interview, making recall more challenging.

For these methodological and pragmatic reasons, individual interviews rather than focus groups will be used. The interviews will aim to elicit the views and opinions from the participants and their mentors on the research problem, the intervention, and their own reflections on the whole process.

Observations

In this particular study, the intervention is primarily educational – training and mentoring in the workplace. Murphy (Murphy et al., 1998) highlights that in educational research, there has been a move towards observational research due to a dissatisfaction with previous research approaches which largely ignored what took place inside the school or classroom. The authors also suggest a parallel situation in healthcare research where typically, there is an emphasis on the study of input and output variables rather than the

study of practices of health professionals or the interactions between clinicians and their patients. As in educational research, observational studies in healthcare settings are seen as a possible solution to this issue, having the potential to study what takes place within the ‘black box’ (Murphy et al., 1998). A similar analysis is offered by Agar (1986) who states that when the social researcher assumes a learning role, research questions and therefore research methods are framed by the need to learn about a world by encountering it firsthand and making some sense out of it, yielding the researcher an understanding of that world. He summarizes this as attempting to answer the question “What is going on here?”

As with qualitative interviews, there are differing approaches to observations which will influence the way that the researcher collects, views and interprets data. The naturalistic approach seeks to faithfully represent subjects' worlds by minimizing the researcher's presuppositions, while the ethnomethodological approach seeks to “describe methods persons use in doing social life” (Sachs, 1984 p21). Miles and Huberman (1984) helpfully point out that the researcher will always enter the field with a certain perspective or focus, and while this is legitimate, it is important to be explicit about this. Silverman (2010) also helpfully distinguishes what the final research report is - a representation of the study setting rather than a reproduction of it. In this case, qualitative observation is being used for one of its strengths – its ability to study process rather than merely to record outcome (Murphy, 1998).

Another reason to include participant observations in this study is the complementarity with interviews. While interviews will provide information, there can be no assurance that what participants say they do is what they actually do and observation goes some way to addressing this problem (Pope and Mays, 2006, Silverman, 2006). It is now commonplace in health research to use observations to corroborate and nuance interview findings (Keen, 2006).

Qualitative observations of the mentoring process will allow the lead researcher to position himself in the process of the mentoring and take field notes on the behaviour and activities of participants and their mentors during the delivery of clinical reasoning facilitation. The lead researcher will be able to record (in the field notes and with audio recording) the dynamic between mentor and participant, as well as to directly witness the intervention in progress. The data obtained from the observations will be used in conjunction with that from interviews to develop themes which in turn will be triangulated with that of the quantitative methods (see mixed methods data analysis procedures section).

The lead researcher’s presence may be perceived as intrusive by the participant and / or the mentor; one half day observation per mentor/participant should be sufficient to provide the qualitative data required, without disrupting the majority of the delivery of the clinical reasoning facilitation.

Other methods considered

Other qualitative data collection methods that could have been used in this study are the use of documents, and audio-visual materials. The use of patient notes could have been utilized to explore participants’ clinical reasoning; this would afford the researcher the participants’ perspective from an unobtrusive source at a time convenient to him, and save the time of transcribing interview material. However, the huge variety in what clinicians articulate in their notes, from basic covering of the “medico-legal bases”, to fuller descriptions, may mean that information on clinical reasoning may be limited, and questions may arise as to how authentic and accurate the notes are. Audio-visual materials – in this case video recording the mentoring process – can be seen as a less obtrusive method of data collection than direct observation; however, the presence of a camera may still be disruptive and affect responses, so for these reasons, direct observation and interviews will be preferred, where audio recordings will be used to capture data.

Challenges in using this type of design and how they will be addressed

Some of the disadvantages of interviews are that the information obtained is indirect, and filtered through the views of interviewees, and collected away from natural field setting; however, by combining the interview information with that of the direct observations from the field setting, a broader picture of the research problem and intervention will be obtained – the data from interviews being far more detailed, and specifying the meaning participants hold about the problem or issue, not the meaning that the researcher brings to the research. Other issues that the lead researcher needs to be mindful of during the interview process are that his presence may bias responses by giving his perspective or by giving advice (teaching) or summarizing too early (counseling), and there may be variability in individuals' abilities to be articulate and perceptive. This will be explored by piloting the interview questions on staff who have completed Masters courses so are ineligible for recruitment, and feedback given using recordings of the interviews.

Observations also present challenges. Unguided research is warned against by multiple authors (Delamont, 2004; Miles and Huberman, 1984; Mason, 1996) because it leads to sloppy, unfocused research, and is based on a false premise that the researcher can just describe or explore the social world. Wolcott (1990) suggests the answer to this is to narrow down the focus of the research, aiming to do less but more thoroughly. Silverman (2010) also proposes similar strategies, stating that the "facts never speak for themselves" and spelling out the need to be clear about the aims of the observations, strictly defining the research problem, using concepts drawn from a particular model, deciding which data to use according to what is most appropriate to the research problem and limiting the amount of data gathered to what can be analyzed readily.

In this study, what needs to be asked is what is of primary interest - what people are thinking or feeling or what people are doing? The reality is that the observations are aimed at the "black box" of the mentoring process, focusing on what mentors and mentees actually do, leaving what they say they 'think' and 'feel' to the interviews. This approach would be supported by Siverman (2010) responding to one of the aims of observational research put forward by Bryman (1998) being to see things from the perspective of the participant being studied; Silverman points out a danger of this aim can be that research can soon degenerate into perspectives about "common sense" or psychology.

The actual data collection process can also present challenges. The presence of the lead researcher may be perceived as intrusive by the participant and / or the mentor; this can lead to a so-called Hawthorne effect, that is modifications to participants' behaviour stimulated by the presence of the observer (Holden and Bower, 1998, Pope and Mays, 2006). This can be minimised as the clinics where data collection will take place are semi-public places, and the observer's presence can be less obtrusive; also the observations will only be a small proportion of the total delivery of the clinical reasoning facilitation.

Challenges can also lie in the analysis and reporting of observational data. Silverman (2010) warns that a preference for an unstructured research design can sometimes lead to a study which merely tells anecdotes about a few choice examples, and elsewhere (Silverman, 2006) highlights that preoccupation with uncovering participants' meanings can result in research that merely reproduces participants' accounts, and neglects meaningful analysis; or by emphasising participants' meanings the study of participants' practices is often lacking. Analysis will be clearly conducted (see section on qualitative and mixed methods analysis) to cover both meaning and practice of participants. There is also the challenge of attempting to study the meaning of actions which are observed, due to the fact that human action is often ambiguous (Murphy et al., 1998). The triangulation of research data – interview and observation, along with quantitative data – will attempt to overcome this: for example, comparing what is said in interview with what is done in observed practice.

Examples of use of this type of design

There are myriads of examples of how semi-structured interviews and observations have been used in health care research. Of particular relevance to this study are the observations and semi-structured interviews used by Jensen (Jensen et al., 2007a, Jensen et al., 2000) and Resnik (Resnik, 2007, Resnik and Jensen, 2003) in the study of expertise in physiotherapy, those of Edwards (Edwards et al., 2004, Edwards and Jones, 2007) in the study of clinical reasoning and those of Rushton (Rushton and Lindsay, 2010) in the study of masters level manipulative physiotherapy education. These examples have identified themes and subsequently influenced the areas of physiotherapy practice and experience the observations and interviews in this study wish to explore.

Recruitment, enrolment and data collection

In order to obtain diverse perspectives, purposive sampling from the group of participating physiotherapists and their mentors as outlined above in the quantitative section will provide the interviewees. This gives a probable starting point of around 10 or 12 participants and 4 to 6 mentors, but data analysis will be an iterative process, with codes and themes being developed (see data analysis section), and this process will be ongoing, continually comparing new data with that previously collected, until no new themes or codes emerge and saturation is reached, and so a smaller sample may result.

Qualitative data collection and analysis

Data Recording Procedures

Qualitative observational research relies on the researcher as instrument documenting the world being observed (Pope and Mays, 2006). A very simple observational protocol will be used for recording data from observations of clinical reasoning facilitation sessions between participants and their mentors (see table 2, (Creswell, 2007)) and will be recorded in the setting. Descriptive notes could include a reconstruction of dialogue, a description of the physical setting, and accounts of activities. Reflective notes could include my thoughts, feelings, and ideas as researcher. The initial notes and observations will be used as a prompt to write up full field notes as soon as possible after the observations. NVIVO 9 software will be utilised to assist in the data management of both forms of qualitative data.

Table 2: Observational Protocol

Observational Protocol	
Demographic Information	
Time & Date:	Participant:
Location:	Mentor:
Descriptive Notes:	***Reflective Notes:***

Qualitative interviews can be recorded by taking notes at the time, notes taken afterwards and by audio recording. Silverman (2010) states that qualitative researchers should always attempt to audio record their interviews. Digital audio recording will be utilised in this research, to capture as much data as possible, and to avoid too much interference of note writing with the interview process (Britten, 2006), although some notes will be taken as recommended by Creswell (Creswell, 2009) using an interview protocol which will be used for asking questions and recording answers during the qualitative interviews (tables 3 and 4). This gives back up data in the event of failure of audio recording devices, which will be tested extensively beforehand to ensure I am familiar with the equipment (Britten, 2006). To guide the interview, a copy of the participant's curriculum vitae will be obtained, and information organized into categories (e.g. education, clinical experience, involvement in professional activities). The information will be placed on note cards, and each participant will be asked to self-assess the important events in his or her professional development by sorting the cards into 3 categories – the events considered (1) most important, (2) somewhat important, and (3) least important. This is a technique used by Jensen (Jensen et al., 2007a, Jensen et al., 2000) in qualitative interviews exploring expertise in physiotherapy. The professional development interview used by Jensen in these studies will be employed –being almost identical apart from some slight alterations for United Kingdom English, and the sample being comprised of musculoskeletal physiotherapists. The rationale for using this interview protocol is that it is a tool that has been developed and used previously in the musculoskeletal physiotherapy population and will thus allow comparisons of research findings with those found in the literature (Jensen et al., 2007a, Jensen et al., 2000, Resnik, 2007, Resnik and Jensen, 2003).

Table 3: Interview Protocol for Participants

**Time & Date:**	**Interviewee:**
**Location:**	**Interviewer:**

Instructions: This interview is to provide me with insight into your perceptions of your work – what you value, how your clinical practice has developed, how your clinical reasoning has changed over time, and what events stimulated these changes. To help guide us in our discussion, I have some cards here with information from your curriculum vitae which you submitted to us; could you place each of these cards into one of three categories to show me the importance of each of these in your professional development: the first category should include the events you consider to be most important, the second category should include what you consider to be somewhat important, and the third category should include what you consider to be least important.

Section A Questions: Mentee Development (adapted from Professional Development Interview, Jensen et al, 2007*)

1. Talk about experiences that have affected how you think about physiotherapy and how you practice.
2. After curriculum vitae categories have been sorted (most important / somewhat important / least important in affecting growth to expertise) discuss each of the categories you have grouped. Why have you grouped these together? What is meaningful about this course? (or person, experience, etc.) [this is done for each of the categories]
3. How has your knowledge of physiotherapy changed over time? How has your knowledge of musculoskeletal outpatients changed over time? Describe an example. To what do you attribute these changes?
4. What aspects of your clinical knowledge have changed most over time? What are the sources for your clinical knowledge?
5. Discuss your typical clinical reasoning pattern or any models you use. How did you acquire your present decision-making style? How has this style changed over the years? Describe an example. What do you believe accounts for these changes?
6. What advice would you give to new graduates wanting to become expert musculo-skeletal physiotherapists?
7. What do you consider to be the milestones in your learning that have led to your becoming the clinician you are today?

Section B Questions: Barriers and Supports to Learning

I'd like us to focus now on the factors that might have influenced how much you have gained from this learning experience. In particular I'd like us to think about you, the mentor, and the environment.

1. Thinking about you, what do you think might be the barriers that hinder your learning? And what factors do you think support your learning? (E.g. past experiences, personal attributes, knowledge, skills?)
2. Thinking about your mentor, what do you think they provided to support your learning? And do you think there is anything he/she brings as mentor that hinders your learning?
3. Thinking about the mentoring process and the environment here, what barriers did they provide to your learning? What supports did they provide to your learning?

Thank-you statement: I'd just like to say thank you very much for taking the time to be interviewed today and for discussing your professional development experiences with me.

Table 4: Interview Protocol for Mentors:

Information from interviews will be recorded digitally. As the researcher, I will also take notes, in the event that recording equipment fails. Transcription of the interviews will be performed with the use of Dragon NaturallySpeaking software, and validated by interviewees.

<table>
<tr><td>Time & Date: Interviewee:

Location: Interviewer:</td></tr>
<tr><td>Instructions: This interview is to provide me with your insight into the mentoring process and its usefulness in facilitating the participants’ clinical reasoning. To help guide us in our discussion, I have grouped questions into 3 broad areas – your perspective on mentoring, your mentee’s performance, and any barriers or supports to learning that your mentee displayed or experienced.</td></tr>
<tr><td>Section A Questions – mentor reflection: (Kelly, 2007)
I’d like to explore the mentoring process from your perspective, first of all.

1. Tell me how you approach working with a mentee?
2. How much of your approach varies mentee to mentee and how much stays the same? What components vary and what stays the same?
3. How much of the learning experience do you view as your responsibility and how much is the mentee’s responsibility?
4. How would you describe your ideal mentee?
5. What type of relationship do you try to develop with mentees?
6. Do you feel valued in your role as a mentor? If so, by whom?</td></tr>
<tr><td>Section B Questions – formative assessment of mentee performance: (Kelly, 2007)
Now I’d like to explore your thoughts on the mentee’s clinical performance.

1. As we page through this Clinical Performance Instrument, tell me which performance criteria you feel are most important? Are critical to address with the mentee? That you spend the most time on?
2. Which criteria best fit your mentee at the end of this mentoring process? Which would have fit best at the start of the mentoring? Can you give any examples to illustrate your choices?</td></tr>
<tr><td>Section C Questions – barriers and supports to mentee’s learning: (Plack, 2008)
Finally, I’d like us to focus now on the factors that might have influenced the mentee’s engagement with this learning experience. In particular I’d like us to think about the mentee, you as the mentor, and the environment.

1. Thinking about your mentee himself/herself, what do you think might be the barriers that hinder learning? And what factors do you think support this mentee’s learning? (E.g. past experiences, personal attributes, knowledge, skills.)
2. Thinking about you as mentor, what do you think you provide to support this mentee’s learning? And do you think there is anything you might bring as mentor that hinders his/her learning?
3. Thinking about the mentoring process and the environment here, what barriers might they provide to this mentee’s learning? What supports might they provide to the mentee’s learning?</td></tr>
<tr><td>Thank-you statement: I’d just like to say thank you very much for taking the time to be interviewed today and for sharing your insight and perspective with me.</td></tr>
</table>

Qualitative Data Analysis & Interpretation:

Pre-analysis:

Before analysing the data, the interviews will need to be transcribed and the field notes from the observations will need to be typed up. Transcription of interviews can be extremely time consuming (Britten, 2006), and so to help with this, Dragon NaturallySpeaking software will be used, and transcripts will be validated by interviewees. Following this all of the data will be read through and notes on general thoughts will be written into the margins in order to obtain a general impression of the information and its meaning reflected upon. Creswell (2009) suggests asking these preliminary questions: "What general ideas are participants saying? What is the tone of the ideas? What is the impression of the overall depth, credibility, and use of the information?"

Analysis:

It is emphasised by many authors that field notes from observations and transcripts of interviews are only the raw material, and do not in and of themselves provide research explanations (Pope and Mays, 2006, Britten, 2006, Silverman, 2006). It is also helpfully highlighted by Silverman (Silverman, 2010) that in the very act of making fieldnotes, the researcher is not simply recording data but also analyzing them, in that the categories used will inevitably be theoretically saturated, either consciously or subconsciously.

Detailed analysis will begin with organizing the interview and observational material from data collection into sections of text data, and categorizing them before labelling the categories with a term. This coding process brings meaning to information (Rossman and Rallis, 2003). Creswell (2009) recommends researchers consider codes on topics that audiences would expect to find, based on clinical experience and previous literature, surprising or unanticipated codes, and codes that are unusual, and that are interesting as concepts in and of themselves to audiences. Silverman (Silverman, 2010) cautions against coding data too rapidly on the basis that it can mean the researcher takes for granted what is being "seen". The coding will then be used to identify and generate themes or categories, on the basis of what appear to be major findings in the qualitative aspect of the study and can be utilised to create headings in the findings sections of the research report, displaying multiple perspectives from the different participants and evidenced by specific examples and diverse quotations. More complex analysis can then be built by interconnecting the themes identified, and discussing these themes in detail in the qualitative narrative section of the research write-up. Computer software can be a useful aid in filing and indexing (Silverman, 2006) and NVivo software will be utilized to this effect in this study. Finally, interpretations will be made to bring meaning to the data. Lincoln & Guba (Lincoln and Guba, 1985) state that asking "What were the lessons learned?" captures the essence of this idea; the lessons learned may well involve my own personal interpretation as researcher. Obviously, these interpretations will be embedded in my understanding, background and experience, which need to be borne in mind throughout the analysis, and identified in the discussion. Meaning will also be derived from a comparison of the findings with information already derived and discussed from the literature. This will allow me to conclude whether the findings support previous theories and information or diverge from them, as well as highlighting new questions that need to be asked, and future directions for research.

Mixed methods data analysis procedures

Integration of both the qualitative and the quantitative components of this research project is an important - if not essential - aspect of mixed methods research (Creswell et al., 2004, Lingard et al., 2008). However, the goal of integration - achieving a knowledge yield that is as a whole greater than the sum of the parts (Barbour, 1999, Creswell, 2009) is often unachieved in healthcare due to the lack of integration leading to

outputs that are equivalent to that from a qualitative study and a quantitative study undertaken independently (Lewin et al., 2009, O'Cathain et al., 2008). In response to this, O'Cathain et al (O'Cathain et al., 2010) recommend three approaches to integration when a pragmatic stance or worldview is adopted by mixed methods researchers: triangulation, following a thread, and use of a mixed methods matrix. The authors state that all three approaches are more suitable for single researchers – as is the case for this research project - than for large research teams, and in particular when there is the availability of both qualitative and quantitative data on the same cases – as there will be in this study – then the use of a mixed methods matrix is desirable as the data can be examined in detail for each case—for example, comparing participants' outcomes with their interview transcript. Data on each case will be summarised and displayed within a mixed methods matrix, where the rows represent the cases for which there is both qualitative and quantitative data, and the columns display the different data collected on each case (Creswell and Plano Clark, 2007, Miles and Huberman, 1994, Wendler, 2001). In this case, the matrix would look as in table 5:

Table 5: Mixed Methods Matrix

Outcomes data from pre-intervention   Outcomes data from post intervention

Cluster	Subject	Quantitative Data										Qualitative Data			
		PSFS differences		EQ-5D differences		MedRisk differences		PAM differences		Therapist Performance differences		Theme 1	Theme 2	Theme3	Theme 4
Cluster 1	PTA														
	PTB														
	PTC														
	PTD														
Cluster 2	PTE														
	PTF														
	PTG														
	PTH														
Cluster 3	PTI														
	PTJ														
	PTK														
	PTL														

This approach will allow me as researcher to observe correlations and paradoxes between types of data on a case by case basis as well as for patterns across all cases. Specifically, during the analysis the researcher looks for convergence - where findings from each method agree, for complementarity - where findings from each method offers complementary information on the same issue, and for discrepancy – where findings from each method appear to contradict each other(Erzberger and Prein, 1997, Farmer et al., 2006, Foster, 1997). As will be discussed shortly in validity approaches for qualitative data analysis, explicitly examining for – and reporting of - discrepancies between findings is important for the integrity and validity of a study. In addition, exploring apparent discrepancies may lead to a better understanding of the research question (Creswell, 2009; (Creswell, 2009, Fielding and Fielding, 1986, O'Cathain et al., 2010).

Validity, Credibility & Trustworthiness in both Quantitative & Qualitative Methods

Quantitative Methods: Identification of threats to validity and responses

Creswell (2009) identifies common threats to both internal and external validity. Of particular relevance to this study, internal threats could be due to history, selection, compensatory processes, testing or instrumentation. History threats can be due to external events that can occur during the time that elapses in an experiment, which have an impact on the outcome besides the experimental treatment. To counter this, the research design uses participants from a single profession (physiotherapy), working in a similar environment (musculo-skeletal outpatient departments) in the same NHS health board (University Health

Board); this means that professionally and organisationally the participants should experience the same external events. Selection threats can be due to participants being selected who have certain attributes that predispose them to have better outcomes (e.g., he or she is already more advanced in clinical reasoning, or is brighter or more likely to respond to this form of educational intervention), thus increasing the risk of bias as highlighted by the Cochrane risk of bias tool (Armijo-Olivo et al., 2010, Lundh and Gotzsche, 2008). To counter this, the study has utilised a crossover design, so that each participant acts as his / her own control. This does raise the interesting question of different participants' learning styles having a hidden but profound effect on learning effectiveness, and this will be explored using a learning styles questionnaire (Honey and Mumford, 2000a, Honey and Mumford, 2000b). Compensatory threats occur when benefits provided in the experimental group leave participants in the control group resentful, demoralized or feeling devalued, skewing results. Again, the crossover design of this trial counters this threat, with each participant acting as their own control, meaning benefits are provided to both groups, and the fact that during the "control" phase of this process usual training is being received. Testing threats occur when participants become familiar with the outcome measure being used and remember their responses for later testing. This is countered by the primary outcome measures being *patient* completed outcome measures, and the long-term follow up meaning that recall of previous responses is highly improbable. Instrument threats occur with changes in outcome measures between a pre-test and post-test; in this case, a broad range of outcomes are used to make any desire for changes in outcome measures unnecessary.

External threats to validity in this study could occur from the narrow characteristics of participants in the experiment, care must be taken not to generalize to individuals who do not have the characteristics of participants. Therefore, while methods and results may be of interest to - and have relevance for - other allied health professions, additional experiments would need to be performed to ascertain whether similar results are elicited. Likewise, due to the narrow characteristics of the setting of participants in this experiment (musculo-skeletal outpatients), care must be taken not to generalize to individuals in other settings (inpatient settings) without further experimentation.

Other threats to validity include statistical conclusion threats which occur from inadequate statistical power or violation of statistical assumptions leading researchers to draw inaccurate inferences or conclusions from the data. In addition, construct validity can be threatened when researchers use inadequate definitions and measures of variables. The measures of variables have already been outlined as appropriate measures for the variables that have been accurately defined, and the research study has been appropriately powered.

Qualitative Methods: Credibility & Trustworthiness

Creswell (2009) states that themes that are established by converging multiple perspectives from participants can claim to possess added validity. And in addition, as has already been highlighted as part of the mixed methods approach in this study, the qualitative data will be triangulated with the quantitative data. The approach of "validating" qualitative data largely depends on the approach of the researcher; Silverman (2006) states that those who adopt a "realist" approach to qualitative data, come from a positivist or emotionalist standpoint, and treat respondents' answers as describing some external reality (such as facts or events) or internal experience (such as feelings or meanings). If a researcher follows this approach, it is appropriate to incorporate various strategies into the research design to ensure the accuracy of the researcher's interpretation, although he stops short of using the term "validity". Silverman (2006, 2010) suggests such measures might include checking the accuracy of what respondents say in interview by other observations (i.e. does what they say match with what they do), or using measures such as inter-coder agreement and computer-assisted qualitative data programs as a means of securing a "fit" between your interpretations and some external reality.

An alternative approach is to treat interview data as actively constructed `narratives' involving activities which themselves demand analysis (Silverman, 2010) or accessing various stories or narratives through which people describe their world (Riessman, 2008; Gubrium and Holstein, 2009). This approach is from a constructionist standpoint, where interviewers and interviewees are always actively engaged in constructing meaning (Silverman, 2006). Rather than be concerned with accurate depictions of `facts' or 'experiences', the researcher takes as their topic how meaning is mutually constructed (Atkinson and Coffey, 2002).

So which of these two strikingly different approaches to conceptualizing this research problem – and, in turn, leading to contrasting ways of looking at interview data – is preferable? Silverman (2006) points out that neither approach is without its problems: realist approaches may de-emphasize the multiple meanings that people attach to what they do, while the constructionist approach to defining a research topic can overcome that problem but may be criticized for losing sight of important substantive issues. The research questions under scrutiny here wish to explore participants' thoughts and feelings regarding their own professional development in general, and their mentoring experience in particular, placing it in an "emotionalist" category; but can the criticism of missing multiple meanings as people speak with "multiple voices" be addressed? Silverman suggests technical solutions to this problem can be asking the same question in several different ways or comparing what respondents say with what they do.

In view of the above, it is my intention to triangulate the different data sources of information (by examining evidence from interviews with participants, interviews with mentors, and observations of the mentoring process) in order to justify themes in a coherent and appropriate manner, to enhance the accuracy of what respondents say in interview by making observations of them during the mentoring process, and to address the fact that participants may attach multiple meanings to what they do. The interview questions will also ask questions about the mentoring process in different ways, to address this latter concern.

Other measures employed to combine insight with rigour in qualitative accounts include an open and honest narrative that clarifies the bias the researcher brings to the study; self-reflection about how the interpretation of the findings is shaped by the researcher's background is a feature of good qualitative research and resonates well with audiences (Creswell, 2009). Openness and honesty should also include the presentation and discussion of information that is contrary to the themes that have been developed; this adds to the realism, credibility, and therefore the validity of an account because clinical experience involves different perspectives that do not always harmonize neatly. While most evidence will build a consensus for a theme, information that contradicts the general perspective of the theme will also be included and discussed. Finally, as well as my background introducing potential bias, it also adds validity. Creswell (2009) states that the greater the experience that a researcher has with participants in their actual setting, the greater the validity of the findings. As a physiotherapist who has spent prolonged time in the field of musculo-skeletal outpatient physiotherapy, and worked in the University Health Board, I am able to provide detail about the people and the clinical context. Also, having undertaken and delivered Masters level mentoring programmes to facilitate clinical reasoning I have developed an in-depth understanding of the phenomenon under study. In short, this experience should lend credibility to the narrative account.

Finally, measures to improve consistency and reliability will also be employed (Gibbs, 2007). In the case of this study, transcripts of interviews will be checked to ensure that errors are not made during transcription. Also, as has already been discussed, a codebook will be used to ensure that definitions and meanings of codes do not drift during the coding process. Also, as a single researcher, a second person will be enlisted to cross-check my codes in order to establish whether similar codes would be attributed to sections of the interview text. Miles and Huberman (1994) recommend a minimum of 80% inter-coder agreement for good qualitative reliability; this can be established using the nVIVO qualitative computer software package.

Economic Evaluation

Rationale for Economic Evaluation

The intervention in this situation is educational, and one of the primary issues with any educational input is cost (McMeeken 2008). Indeed, the implications of training costs has been considered at government level, and was one of the key factors in the rationale for the set up of the National Health Service University (NHSU), in order to provide its own training and so reduce expenditure to higher education institutions used by the NHS to train and develop its staff (Flood, 2003). Ironically, the NHSU failed to get off the ground and the nascent institution was abolished in 2005 at an alleged loss of at least £72 million (Flood, 2005, Harding, 2005, Scott, 2004, Taylor et al., 2007). The consideration of cost of training is evident throughout the healthcare literature (Currie et al, 1997; Hlusko et al, 1998, Flood, 2003, Carlisle 2009, Kalet, 2010) and studies examining the efficacy of training programmes have begun to include economic evaluation components (Akkers et al, 2006; Goodwin et al, 2001; Lundberg et al, 2010; Wingard et al, 2004). Any improvements in other outcomes need to be measured against costs of intervention (Jette and Latham 2010).

Aim of Economic Evaluation

The aim of the economic evaluation is to assess the costs associated with the training of physiotherapy staff from the perspective of the UK NHS and to utilise measures of effect alongside the costs to determine the extent to which it can be regarded as an efficient use of NHS funds.

Design of Economic Evaluation

The economic evaluation will provide a comparative analysis of the alternative training programmes available to participants (usual training only verses the clinical reasoning intervention plus access to usual service provision). A cost-consequences analysis will provide a clear descriptive summary of the costs (cost of the intervention programme / cost of the alternative programme – no clinical reasoning intervention) and the consequences (outcomes for the participants / service utilization/utility gains). Costs and consequences will be reported separately enabling a full evaluation of the different outcome components (changes in function/HRQL/activation/resource utilization/utility gains) and related to the costs for each of the alternative courses of action. This information is useful to policy makers in determining whether the intervention is 'worth it' in relation to its generalisability. Further, distinction will be made between the costs incurred and outcomes generated in each programme area, so as to assess variation and potential for efficiency gains. Further, a series of sensitivity analyses will be undertaken to assess implications of parameter variation and a threshold analysis used to determine extent to which costs and outcomes need to vary to alter conclusions relating to relative cost-effectiveness of programme.

Economic Evaluation Methods

Costs will be categorised according to whether they are research or programme specific and care will be taken with regard to the attribution factors used to determine costs of intervention programme.

Further, the agencies that incur costs will be clearly specified, as will the agencies that benefit from reductions in resource utilisation, so as to enable inter-sectoral comparisons to be undertaken. Programme outcomes will be utilised within the economic evaluation in conjunction with the costs to inform the cost consequences analysis and to determine the extent to which the programme can be regarded as representing value for money.

All inputs and services provided – from all agencies and families participating - will be documented in descriptive terms and wherever possible translated into monetary terms using appropriate published unit cost data (e.g. BNF, PSSRU, NHS Reference Costs).

A series of cost modules will be developed, to establish a profile of costs and establish the agency that incurred the costs, as outlined in table 6 below.

Table 6: Cost modules

Type:	Aspects:	Specifics:	Collected By:
Research & Evaluation Specific	Questionnaire Design and Production<br><br>Data Collection, Processing & Management	Printing Costs<br><br>Down time for mentors / participants from patient care for interviews, assessment of therapist performance	Lead Researcher
Training Programme Specific	Direct Programme Costs (i.e. costs associated with the training of physiotherapists)	Down time from patient contact by mentor / trainer to deliver training<br><br>Down time from patient contact by participant to receive training<br><br>Equipment required to deliver training	Monthly Training Requirements Form (Appendix E)
	Indirect Programme Costs	Costs of participation, transport for participants or mentors (if / where applicable)	Monthly Training Requirements Form (Appendix E)
Service Utilisation Costs	Costs of physiotherapy intervention	Number, length and type (individual /group) of attendances	Physiotherapy records / D&T system
	Costs associated with the uptake of traditional health/social care services	e.g. GP, hospital visits; referral to secondary care for opinion or intervention, or for diagnostic imaging; use of prescription medication	Baseline, Discharge and 12 month follow up questionnaires (Appendix A)<br><br>Finance Department, University Health Board

<u>Costs of usual training and clinical reasoning programmes</u>

Overall costs of providing the training programmes will be computed and include the costs of implementing and delivering the programmes and any costs incurred by participants (e.g. Transport to another site to receive training). The overall cost per participant will be computed, for the overall study and for each site. However, a clear demarcation will be made between set up costs, implementation costs and those that would occur if the programmes were to become part of subsequent mainstream provision.

The costs of implementing and delivering the programmes will include staff time, venue and equipment costs, provision of support facilities, materials utilised etc.

As mentors and trainers are supplied from within the physiotherapy service, the staffing costs associated will be readily available. Therefore the actual salary costs of mentors will be obtained and then multiplied by the hours of non-patient contact time as part of the mentoring to derive the average staffing costs for facilitators. The non-patient contact time of mentees will also be calculated in the same way. The Monthly Training Requirements Form (Appendix F) will be entered into an Excel database, which will be forwarded to the lead researcher for analysis with assistance from the research supervisor. This would generate the information necessary for computing the cost associated with providing each mentoring programme.

Service utilisation

Participation by physiotherapists in the training programmes is likely to result in changes in the outcomes of patients being treated by those physiotherapists, including their utilisation of physiotherapy services, and utilisation of other services provided by a range of agencies. These need to be captured within the trial so as to identify the extent of costs offset as a result of the training programmes, and which represent one aspect of the outcomes generated by the programmes. This will be achieved via section B of data collection questionnaires during the study period, to establish service utilisation of patients at baseline and changes on discharge from treatment and over time (long term follow up at 12 months).

The combination of changes in service utilisation over time and costs of programme delivery will result in a net cost per participating physiotherapist, which will be used in the assessment of the relative cost-effectiveness of the programmes. The extent to which these changes in service utilisation are sustained over time inform mentors, staff and key stakeholders in order to assess the longer term efficiency of the programmes.

Analysis will be conducted within the context of representing the data at 'that point in time'. No variables from the main database will be changed, but variables representing costs will be added for the specific purpose of the economic evaluation only. Therefore as the project continues, a second analysis of the service utilisation data will be conducted using the baseline and discharge follow up data as it stands at 'that point in time', capturing any changes made to the data, and a second interim report with accompanying SPSS/Excel file will be produced. A third and final analysis at 12 months will use the data representing the baseline, discharge and the 12 month follow up data points as they stand at 'that point in time'- the final report with accompanying SPSS/Excel file will form part of the PhD thesis. Cost data may also change over the time periods and will be updated when necessary. For the final analysis changes in resources utilised over time will be calculated and used in conjunction with the costs of delivering the programmes to generate the overall cost of programme delivery.

Outcomes

The changes in primary and secondary outcomes will represent the consequences of the delivery of training programmes to be used in conducting the cost consequences analysis. As part of this process the responses to EQ5D at various time-points will be used to generate utilities to derive QALYs gained as a result of the programmes and compute the cost/QALY ratio.

Data analysis

The cost consequences analysis will be conducted from the perspectives of the UK NHS to determine the extent to which the programme can be considered to represent value for money. The cost consequences will

be assessed at the end of the trial and post-trial modelling will be employed to assess the cost consequences for over longer time horizons than possible with the study period.

Changes in resources utilised (4.2) over time will be calculated and used in conjunction with the costs of delivering the programme (4.1) to generate the overall cost of programme delivery per participant and per patient, which will represent the incremental cost of providing clinical reasoning training relative to usual training provision

The changes in primary and secondary outcomes - (including changes in utility scores derived from the EQ5D responses at baseline and 1 year follow-up) will be used alongside the net cost of training programme delivery in the cost consequences analysis to generate a set of indicators of relative cost-effectiveness within the study period, based on incremental cost and incremental outcomes.

Missing data will be dealt with by employing an appropriate imputation-based method for effectiveness and quality of life data (Brazier et al., 2007) while the usual method for dealing with censored data relating to costs will be to employ the weighted cost method with known cost histories (Young, 2005).

Future costs and benefits will be discounted at the prevailing rate (currently 3.5%pa) to bring into present values. Costs and benefits will be discounted at 0% and 6% in the sensitivity analysis.

A series of one-way sensitivity analyses will be undertaken to assess the extent to which changes in the variables employed affect the baseline estimates. The variables will be adjusted in line with emerging distributions of data values and impact on baseline estimates computed. A threshold analysis will also be undertaken to determine the degree of parameter variation required to alter conclusions derived from baseline findings.

A series of scenarios will be developed and utilised within an economic model to assess the relative efficiency of clinical reasoning training over longer time horizons

Probabilistic sensitivity analysis will be undertaken to ascertain cost-effectiveness acceptability curves produced to assess the probability that clinical reasoning training represents value for money

Potential Ethical Issues

Clarification of the Participants' Experiences

Before considering the ethical issues that apply to this research study, it is important to clarify that there are two perspectives to view the ethical issues from – the participating physiotherapists, who are the main subjects of the study, and their patients whose outcome data will be used as the primary research variable for measurement. It is also worth clarifying the understanding of what differences to the usual delivery and receipt of physiotherapy these two groups will experience as a direct result of being part of this research study.

Deviation from normal experience – Therapist perspective:

Each participating physiotherapist needs to understand that the primary variable being measured is patient outcome. The intervention is a course of training in, and facilitation of, clinical reasoning. This training will be context-specific - i.e. based in the clinical context, by means of watched patient assessment and treatment sessions followed by immediate reflection, discussion and feedback sessions. All physiotherapy staff working in musculo-skeletal outpatient departments in Cardiff and Vale University Health Board currently undertake monthly mentoring sessions, and therefore this procedure is not deviating from normal practice for staff or patients. What will differ is the quantity and quality of these sessions - the main differences being related to the more intensive clinical placement model of implementation - and the fact that they will undergo assessments of their performance by an independent assessor.

Deviation from normal experience – Patient perspective:

Patients will receive their normal physiotherapy and as patients currently complete questionnaires and scoring scales as outcome measures for departmental purposes, there is no change to their normal management. Consent will be required due to the range of outcome measures collected and for telephone contact at 12 months, as it is the intention of this study to perform long term follow up. Patient contact details would need to be securely stored.

Sim and Wright (Sim and Wright, 2000) point out that almost all research involving human beings will either directly or indirectly give rise to ethical issues, and that these issues are often more pressing in healthcare on account of individuals being in a potentially vulnerable state due to their disability or illness. With respect to the design and conduct of a research project, the same authors suggest dividing the issues into the following five categories for consideration, in order to balance the methodological and moral aspects of the research: informed consent, privacy and confidentiality, deception, risk of harm, and exploitation.

Ethical Considerations

Informed Consent

Sim (1986 p584) defines informed consent as "the voluntary and revocable agreement of a competent individual to participate in a therapeutic or research procedure, based on an adequate understanding of its nature, purpose, and implications". Elsewhere (Sim, 2004) he expands the specific nature of this into different elements – information, requiring disclosure and comprehension, and consent, requiring voluntariness and competence. Each of these elements needs to be present for informed consent to be valid (Sim and Wright, 2000).

Disclosure concerns the adequacy of the account provided by the researcher to the participant which is judged by the content of this information – does it cover the issues that the participant feels are relevant in coming to a decision (Sim, 2004)? In this instance, the main participants are physiotherapists, but the study requires their patients to participate as the primary variable being measured is patient outcome. This requires information to be disclosed to both physiotherapist and patient, and information sheets (see appendices B and C) have been produced in accordance with National Research Ethics Service guidance (NRES, 2009) and submitted as part of the ethics application to the Research Ethics Committee for Wales.

Disclosure alone is insufficient, however, to obtain informed consent – comprehension on the part of the participant must also be present (Sim, 2004). While disclosure is concerned with the details imparted by the researcher, and the sufficiency of the information, comprehension is concerned with the message received by the patient and its intelligibility (Sim and Wright, 2000). While in this study, for the participating physiotherapists this should not be a major issue, there has been adaptation of professional jargon and technical terminology in the information given to patients (Sim, 2004) as seen in the contrast between the information sheets in appendices B and C.

The element of competence is concerned with the ability of the participant to reach a rational, autonomous decision (Sim and Wright, 2000). This is of significant concern when individuals with appreciable cognitive impairment are the subjects of the research or treatment, and are unlikely to be competent to consent. In this instance, the participating physiotherapists are all likely to be competent to consent, as are the vast majority of patients attending musculoskeletal outpatients departments, although this issue will be considered in each case should questions of competence arise.

Voluntariness is the extent to which the participant feels genuinely free to either grant or refuse consent, and in that it is marked by the absence of inappropriate pressure or influence, or coercion or inducement to agree (Sim, 2004, Sim and Wright, 2000). Participating physiotherapists must be reassured that refusal to consent to be part of this study will not influence their receipt of training or have any other impact on their professional development, and participating patients must be assured that refusal to consent to complete outcome data for this study will have no impact on their care. As lead researcher, I must take care that my words and actions do not result in a lack of voluntariness, and also physiotherapists – when consenting patients – must be aware of any unequal power relationship between them and their patients does not result in the same (Goodyear-Smith and Buetow, 2001). Both participating physiotherapists and patients will be given 24 hour cooling off periods after giving consent, and will be given clear written advice that they are free to withdraw from the trial at any time. In all of this, the principle of respect for autonomy must be upheld, and participants' rights to self-determine fully recognized, making every effort to discover the viewpoints and values of participants, and taking them seriously (Clapton and Kendall, 2002).

Privacy & confidentiality

While privacy is concerned with access to other people, or to information about them, the issue of confidentiality is more specific - relating to the way researchers treat the information gained about others (Sim, 1996). A person's privacy can be invaded by gaining illegitimate access to their personal affairs, but that person's confidentiality would only breached if this information is conveyed to others (Sim and Wright, 2000). This would have relevance in this study if information was to be obtained from a participating physiotherapist who had given their full consent, but that information was then disseminated against their wishes. Privacy would not have been breached, but confidentiality would have. In order to address this, it is important to consider a third, distinct element – anonymity. Anonymity is concerned with whether or not a person's personal information, is attributable to that person. Participants may happily concede a significant measure of their privacy, and be content for the information they share to be quoted, on condition that they

are not able to be identified as the source of the information or opinions expressed (Sim and Wright, 2000). Participating physiotherapists will be interviewed, and as has already been highlighted in the methodology section, focus groups have been rejected as a means of data collection due to the fact that anonymity must be maintained. Care must also be taken during the write up of the research report that any quotes will not be attributable to the physiotherapists involved. Physiotherapists would in all likelihood wish to receive feedback on their patient outcomes and measures of performance, but comparisons would be unhelpful if they were taken personally, therefore privacy must be respected and anonymity is essential. All such data will remain confidential, only the researcher and the participant knowing the results of that assessment process. Patients' contact details will be securely stored to ensure that their privacy is maintained.

Deception

Research may sometimes involve deception in a number of ways; an example of deception by omission would be the withholding of the true nature and objectives of a study from participants who are led to make incorrect assumptions about its purpose. An example of deception by commission would be the communication of facts known to be untrue to research participants. When deception is practiced, it is usually with the express intention of satisfying a methodological requirement of the study concerned (Sim, 2004). No such requirements exist for this study.

Risk of Harm

Participants in research studies may undergo harm in several ways (Seale and Barnard, 1999). Studies involved in testing of physical, pharmacological or behavioural treatment procedures may carry the risk of physical or psychological harm. Studies involved in exploring subjects attitudes and beliefs may carry the risk of psychological harm resulting from the uncovering of sensitive issues or feelings of guilt or inadequacy. Psychological harm may also be the result of the participants having false hopes raised in studies. Harm can also be social - if sensitive information about individuals became open to public scrutiny and participants were subsequently vilified, ostracised, and discriminated against. Individuals could also be subject to economic or legal harm if they were dismissed from their employment as a result of participating in the research, or if illegal behaviours were revealed during the research study (Sim, 2004, Sim and Wright, 2000). There are no apparent risks of harm for patients in this study as they will receive their normal physiotherapy and will complete questionnaires and scoring scales as outcome measures as is usual practice for departmental purposes. While participating physiotherapists will be watched and given feedback by their mentors, and their performance measured by an independent assessor, and this will involve a degree stress as they are subject to scrutiny, the mentoring relationship should always be constructive, and the independent assessment will remain confidential thus reducing excessive stress.

Exploitation

Participants can be exploited if they are used for research in ways that disregard their wishes, dignity or welfare. This area of exploitation shares significant features which overlap with those already outlined in the informed consent and risk of harm sections. The measures already identified will ensure that participating physiotherapists and patients will not be exploited in this study.

Underlying Ethical principles

Sim (Sim, 1997) outlines five fundamental ethical values or principles and suggests that researchers trace back any ethical issues related to their research to one or more of these principles as a means of exploring and analyzing the ethical implications of aspects of the research process. The processes that have been put

in place to gain informed consent from both participating physiotherapists and patients, without deceiving or exploiting them and respecting their confidentiality, privacy and anonymity can be traced back to the fundamental principle of respect for autonomy, being mindful as researcher that I need to protect the self determinance of others (Sim and Wright, 2000). The requirement of this research study to gain informed consent without exploiting participants can also be traced back to the underlying principle of respect for persons, as is the requirement to ensure that the research procedures do not undermine the participant's dignity or self respect (Sim and Wright, 2000). Therefore, as researcher, I should ensure that the people involved in this research study are treated with consideration of their individual human dignity and are not use them as a means to an end. This is seen by patients receiving their usual physiotherapy treatment whether they consent to be part of this study or not, and physiotherapists receiving their usual professional development irrespective of their involvement in this study; and for those who do participate, individual feedback will be given constructively to enhance and develop their level of expertise on an individual basis. The very act of carrying out this research is to answer the research questions outlined earlier, namely whether this educational intervention will not just benefit the development of physiotherapists, but also benefit the patients that they treat in tangible outcome improvements. The need to carry out research whose findings will benefit members of society, and the need to disseminate the findings of such research can be traced back to the ethical principle of beneficence, acting in a way that will bring benefit to people and promote their well-being (Sim, 2004). This principle is distinct from another fundamental ethical principle, non-maleficence, where the researcher refrains from acting in a way that will bring harm to other people. In this study, this is reflected by the measures taken to avoid causing harm or distress, taking care not to breach confidentiality or anonymity, and being mindful not to raise expectations that are unrealistic (Sim and Wright, 2000). The fact that no vulnerable individuals will be used in this research study, and the decision to use a crossover design which ensures that the benefits and burdens of the research study are distributed fairly and evenly is linked to the final principle of justice, dealing with others in a manner that is fair and consistent with individual merit (Sim, 2004).

Gaining Ethical approval

Ethical approval is required and will be sought from the Research Ethics Committee for Wales. Sim and Wright (Sim and Wright, 2000) helpfully emphasize that researchers should remember that approval from a research ethics committee does not mean that the ethical aspects of the study need no further attention from the investigator once approval is given; instead ethical considerations should be given continuing scrutiny as the study proceeds.

References & Appendices

(1990) *Health Policy (Amsterdam, Netherlands),* **16,** 199-208.
(2001) World Health Organization, Geneva, Switzerland.
(2007) (Ed, Health, D. o.) DH Publications, London.
Agapova, O. A., Person, E. and Harbour, J. W. (2010) *Clin Exp Metastasis,* **27,** 91-6.
Alderson, D. (2010) *Medical Teacher,* **32,** 830-836.
Armijo-Olivo, S., Stiles, C. R., Hagen, N. A., Biondo, P. D. and Cummings, G. G. (2010) *J Eval Clin Pract*.
Bailet, L. L., Repper, K. K., Piasta, S. B. and Murphy, S. P. (2009) *Journal of Learning Disabilities,* **42,** 336-355.
Bailey, I. W. and Archer, L. (2004) *Water Sci Technol,* **50,** 105-10.
Barbour, R. S. (1999) *J Health Serv Res Policy,* **4,** 39-43.
Bazeley, P. (2009) In *Mixed methods research for nursing and the health sciences*(Eds, Andrew, S. and Halcomb, E. J.) Wiley-Blackwell, Oxford, pp. 84-118.
Beattie, P., Turner, C., Dowda, M., Michener, L. and Nelson, R. (2005) *J Orthop Sports Phys Ther,* **35,** 24-32.
Beattie, P. F., Nelson, R. and Murphy, D. R. (2011) *J Manipulative Physiol Ther,* **34,** 23-9.
Beattie, P. F., Nelson, R. M. and Lis, A. (2007) *Phys Ther,* **87,** 793-800.
Beattie, P. F., Pinto, M. B., Nelson, M. K. and Nelson, R. (2002) *Physical Therapy,* **82,** 557-565.
Bekkering, G. E., van Tulder, M. W., Hendriks, E. J., Koopmanschap, M. A., Knol, D. L., Bouter, L. M. and Oostendorp, R. A. (2005) *Phys Ther,* **85,** 544-55.
Benner, P., Hooper-Kyriakidis, P. and Stanard, D. (1999) *Clinical wisdom and interventions in critical care.,* W B Saunders, Philadelphia.
Benner, P., Tanner, C. and Chesla, C. (1996) *Expertise in nursing practice.,* Springer, New York.
Benner, P., Tanner, C. A. and Chesla, C. A. (1997) *American Journal of Nursing,* **97,** 16BBB-16BBB.
Bergman, M. M. (2010) *Journal of Mixed Methods Research,* **4,** 171-175.
Boshuizen, H. P. A. and Schmidt, H. G. (2008) In *Clinical Reasoning in the Health Professions*, Vol. 1 (Eds, Higgs, J., Jones, M. A., Loftus, S. and Christensen, N.) Butterworth-Heinemann.
Brazier, J., Ratcliffe, J., Salomon, J. A. and Tsuchiya, A. (2007) *Measuring and valuing health benefits for economic evaluation.,* Oxford University Press, Oxford.
Brentnall, D. and Sterling, M. (2007) *Australian Journal of Physiotherapy,* **53,** 65-65.
Britten, N. (2006) *Qualitative Interviews,* Blackwell Publishing Ltd.
Brown, C., Hofer, T., Johal, A., Thomson, R., Nicholl, J., Franklin, B. D. and Lilford, R. J. (2008) *Quality & Safety in Health Care,* **17,** 163-169.
Brown, C. A. and Lilford, R. J. (2006) *BMC Med Res Methodol,* **6,** 54.
Brown, G. (2008) *Occup Med (Lond),* **58,** 447-8.
Bye, A. M., Connolly, A. M., Farrar, M., Lawson, J. A. and Lonergan, A. (2009) *J Paediatr Child Health,* **45,** 727-30.
Campbell, M. J. (2004) *BMJ,* **328,** 654-5.
Campbell, M. K., Elbourne, D. R. and Altman, D. G. (2005) *Med Clin (Barc),* **125 Suppl 1,** 28-31.
Campbell, M. K., Mollison, J., Steen, N., Grimshaw, J. M. and Eccles, M. (2000) *Fam Pract,* **17,** 192-6.
Casserley-Feeney, S. N., Phelan, M., Duffy, F., Roush, S., Cairns, M. C. and Hurley, D. A. (2008) *BMC Musculoskelet Disord,* **9,** 50.
Celebi, N., Weyrich, P., Riessen, R., Kirchhoff, K. and Lammerding-Koppel, M. (2009) *Med Educ,* **43,** 1010-8.
Chan, A. W. and Altman, D. G. (2005) *Lancet,* **365,** 1159-62.
Chatman, A. B., Hyams, S. P., Neel, J. M., Binkley, J. M., Stratford, P. W., Schomberg, A. and Stabler, M. (1997) *Phys Ther,* **77,** 820-9.
Chen, F. M., Bauchner, H. and Burstin, H. (2004) *Acad Med,* **79,** 955-60.
Cherryholmes, C. H. (1992) *Educational Researcher,* **21,** 13-17.
Cho, J., Kang, G. H., Kim, E. C., Oh, Y. M., Choi, H. J., Im, T. H., Yang, J. H., Cho, Y. S. and Chung, H. S. (2008) *Emerg Med J,* **25,** 732-4.
Christensen, N., Jones, M. A., Higgs, J. and Edwards, I. (2008) In *Clinical Reasoning in the Health Professions*, Vol. 1 (Eds, Higgs, J., Jones, M. A., Loftus, S. and Christensen, N.) Butterworth-Heinemann.
Clapton, J. and Kendall, E. (2002) *Disability & Rehabilitation,* **24,** 987-991.
Cleland, J. A., Fritz, J. M., Brennan, G. P. and Magel, J. (2009) *Phys Ther,* **89,** 38-47.
Cleland, J. A., Fritz, J. M., Whitman, J. M. and Palmer, J. A. (2006) *Spine (Phila Pa 1976),* **31,** 598-602.

Cook, A., Khoury, A., Bagli, D., McLorie, G. A., El-Ghoneimi, A. and Farhat, W. A. (2005) *The Canadian Journal Of Urology,* **12,** 2824-2828.

Cook, D. A., Beckman, T. J., Thomas, K. G. and Thompson, W. G. (2008) *Med Educ,* **42,** 838-48.

Cook, D. A., Gelula, M. H., Dupras, D. M. and Schwartz, A. (2007) *Med Educ,* **41,** 897-905.

Cook, D. A., Thompson, W. G. and Thomas, K. G. (2009) *Acad Med,* **84,** 1419-25.

Cook, D. A., Thompson, W. G., Thomas, K. G., Thomas, M. R. and Pankratz, V. S. (2006) *Acad Med,* **81,** 231-8.

Costa Lda, C., Maher, C. G., McAuley, J. H., Hancock, M. J. and Smeets, R. J. (2011) *European Journal of Pain,* **15,** 213-219.

Costa, L. O. P., Maher, C. G., Latimer, J., Hodges, P. W., Herbert, R. D., Refshauge, K. M., McAuley, J. H. and Jennings, M. D. (2009) *Physical Therapy,* **89,** 1275-1291.

Crespo, K. E., Torres, J. E. and Recio, M. E. (2004) *J Dent Educ,* **68,** 1235-44.

Creswell, J. W. (2007) *Qualitative Inquiry and Research Design: Choosing Among Five Approaches,* Sage, Thousand Oaks, CA.

Creswell, J. W. (2009) *Research Design: Qualitative, Quantitative, and Mixed Methods Approaches,* Sage, Los Angeles.

Creswell, J. W., Fetters, M. D. and Ivankova, N. V. (2004) *Ann Fam Med,* **2,** 7-12.

Creswell, J. W., Guttman, M., Plano Clark, V. L. and Hanson, W. (2003) In *Handbook of Mixed Methods in Social & Behavioral Research*(Eds, Tashakkori, A. and Teddlie, C.) Sage, Thousand Oaks, CA.

Creswell, J. W. and Plano Clark, V. L. (2007) *Designing and Conducting Mixed Methods Research,* Sage, Thousand Oaks, CA.

Dall'Alba, G. and Sandberg, J. (2006) *Review of Educational Research,* **76,** 383-412.

DiGuiseppi, C. and Coupland, C. (2010) *Injury Prevention,* **16,** 61-67.

Dolan, P. (1997) *Medical Care,* **35,** 1095-1108.

Downing, A. M. and Hunter, D. G. (2003) *Manual Therapy,* **8,** 117-119.

Dreyfus, H. L. and Dreyfus, S. E. (1996) In *Expertise in nursing practice.*(Eds, Benner, P., Tanner, C. A. and Chesla, C. A.) Springer, New York, pp. 29-48.

Dunstan, D. A., Covic, T., Tyson, G. A. and Lennie, I. G. (2005) *Int J Rehabil Res,* **28,** 369-70.

Edwards, I., Jones, M., Carr, J., Braunack-Mayer, A. and Jensen, G. M. (2004) *Phys Ther,* **84,** 312-30; discussion 331-5.

Edwards, I., Jones, M. and Hillier, S. (2006) *Man Ther,* **11,** 2-10.

Edwards, I. and Jones, M. A. (2007) In *Expertise in Physical Therapy Practice (Second Edition)*W.B. Saunders, Saint Louis, pp. 192-213.

Edwards, S. J., Braunholtz, D. A., Lilford, R. J. and Stevens, A. J. (1999) *BMJ,* **318,** 1407-9.

Eldridge, S., Ashby, D., Bennett, C., Wakelin, M. and Feder, G. (2008) *BMJ: British Medical Journal (International Edition),* **336,** 876-880.

Eldridge, S. M., Ashby, D., Feder, G. S., Rudnicka, A. R. and Ukoumunne, O. C. (2004) *Clinical Trials (London, England),* **1,** 80-90.

Engers, A. J., Wensing, M., van Tulder, M. W., Timmermans, A., Oostendorp, R. A., Koes, B. W. and Grol, R. (2005) *Spine (Phila Pa 1976),* **30,** 559-600.

Eraut, M. (2001) *Medical Education,* **35,** 8-11.

Eraut, M. (2004) *Learning in Health & Social Care,* **3,** 171-178.

Eraut, M. (2005) *Learning in Health & Social Care,* **4,** 173-179.

Eraut, M. (2006a) *Learning in Health & Social Care,* **5,** 111-118.

Eraut, M. (2006b) *Learning in Health & Social Care,* **5,** 1-8.

Erzberger, C. and Prein, G. (1997) *Quality & Quantity,* **31,** 141-154.

Escorpizo, R., Ekholm, J., Gmunder, H. P., Cieza, A., Kostanjsek, N. and Stucki, G. (2010) *J Occup Rehabil,* **20,** 502-11.

Fairbairn, K., May, K., Yang, Y., Balasundar, S., Hefford, C. and Abbott, J. H. (2010) *New Zealand Journal of Physiotherapy,* **38,** 69-69.

Farmer, T., Robinson, K., Elliott, S. J. and Eyles, J. (2006) *Qual Health Res,* **16,** 377-94.

Fern, E. F. (1982) *Journal of Market Research,* **19,** 1-13.

Fielding, N. G. and Fielding, J. L. (1986) *Linking Data,* Sage, Los Angeles.

Fikkers, B. G., van Vugt, S., van der Hoeven, J. G., van den Hoogen, F. J. and Marres, H. A. (2004) *Anaesthesia,* **59,** 1008-11.

Fincher, R. M., White, C. B., Huang, G. and Schwartzstein, R. (2010) *Acad Med,* **85,** 821-8.
Fineout-Overholt, E. and Johnston, L. (2007) *Worldviews Evid Based Nurs,* **4,** 54-9.
Fish, D. and Higgs, J. (2008) In *Clinical Reasoning in the Health Professions*, Vol. 1 (Eds, Higgs, J., Jones, M. A., Loftus, S. and Christensen, N.) Butterworth-Heinemann, Philadelphia.
Flood, G. (2003) *Information World Review*, 17-18.
Flood, S. (2005) *The Health Service Journal,* **Suppl,** 3.
Foster, R. L. (1997) *ANS Adv Nurs Sci,* **20,** 1-12.
Fowles, J. B., Terry, P., Xi, M., Hibbard, J., Bloom, C. T. and Harvey, L. (2009) *Patient Education and Counseling,* **77,** 116-122.
Fransen, M. and Edmonds, J. (1999) *Rheumatology (Oxford, England),* **38,** 807-813.
Freemantle, N. (2001) *BMJ (Clinical Research Ed.),* **322,** 989-991.
Friedly, J., Standaert, C. and Chan, L. (2010) *Physical Medicine & Rehabilitation Clinics of North America,* **21,** 659-677.
Gabel, C. P., Melloh, M., Yelland, M., Burkett, B. and Roiko, A. (2010) *Eur Spine J.*
Gibbs, G. R. (2007) In *The Sage qualitative research kit.*(Ed, Flick, U.) Sage, London.
Gielen, A. C., Wilson, M. E. H., McDonald, E. M., Serwint, J. R., Andrews, J. S., Hwang, W. and Wang, M. (2001) *Archives of Pediatrics & Adolescent Medicine,* **155,** 42-49.
Goodyear-Smith, F. and Buetow, S. (2001) *Health Care Analysis,* **9,** 449-462.
Gray, A. M., Clarke, P. M., Wolstenholme, J. L. and Wordsworth, S. (2010) *Applied Methods of Cost-effectiveness Analysis in Healthcare.,* Oxford University Press, Oxford.
Green, C. A., Perrin, N. A., Polen, M. R., Leo, M. C., Hibbard, J. H. and Tusler, M. (2010) *Adm Policy Ment Health,* **37,** 327-33.
Gruppen, L. D. (2007) *Teaching & Learning in Medicine,* **19,** 331-335.
Gwyer, J., Jensen, G., Hack, L. and Shepard, K. (2004) In *Qualitative Research in Evidence-Based Rehabilitation*(Eds, Karen Whalley, H., Ph, D. M. O. T. D., Christine, C. and Ph, D. M. A. B. A. D.) Churchill Livingstone, Oxford, pp. 103-115.
Harding, M.-L. (2005) *The Health Service Journal,* **115,** 10.
Hefford, C., Kemp, L., Abbot, J. H., Arnold, R., Baxter, G. D. and Taylor, W. (2009) *Physical Therapy Reviews,* **14,** 3-4.
Hefford, C., Lodge, S., Elliott, K. and Abbott, J. H. (2008) *New Zealand Journal of Physiotherapy,* **36,** 41-48.
Hemming, K., Girling, A. J., Sitch, A. J., Marsh, J. and Lilford, R. J. (2011) *BMC Med Res Methodol,* **11,** 102.
Heymans, M. W., van Buuren, S., Knol, D. L., Anema, J. R., van Mechelen, W. and de Vet, H. C. (2010) *Spine Journal,* **10,** 847-856.
Hibbard, J. H., Greene, J. and Tusler, M. (2009) *Am J Manag Care,* **15,** 353-60.
Hibbard, J. H. and Mahoney, E. (2010) *Patient Educ Couns,* **78,** 377-81.
Hibbard, J. H., Mahoney, E. R., Stock, R. and Tusler, M. (2007) *Health Serv Res,* **42,** 1443-63.
Hibbard, J. H., Mahoney, E. R., Stockard, J. and Tusler, M. (2005) *Health Serv Res,* **40,** 1918-30.
Hibbard, J. H., Stockard, J., Mahoney, E. R. and Tusler, M. (2004) *Health Serv Res,* **39,** 1005-26.
Hibbard, J. H. and Tusler, M. (2007) *J Ambul Care Manage,* **30,** 2-8.
Higgs, J., Burn, A. and Jones, M. (2001) *AACN Clin Issues,* **12,** 482-90.
Higgs, J. and Jones, M. A. (2008) In *Clinical Reasoning in the Health Professions*, Vol. 1 (Eds, Higgs, J., Jones, M. A., Loftus, S. and Christensen, N.) Butterworth-Heinemann.
Higgs, J. and Loftus, S. (2008) In *Clinical Reasoning in the Health Professions*(Eds, Higgs, J., Jones, M. A., Loftus, S. and Christensen, N.) Butterworth-Heinemann.
Hockings, R. L., McAuley, J. H. and Maher, C. G. (2008) *Spine (Phila Pa 1976),* **33,** E494-500.
Holden, J. and Bower, P. (1998) *Journal Of Health Services Research and Policy,* **3,** 198.
Holyoak, K. J. (1991) In *Toward a general theory of expertise.*(Eds, Ericsson, K. A. and Smith, J.) Cambridge University Press, New York, pp. 301–336.
Honey, P. and Mumford, A. (2000a) *The Learning Styles Helper's Guide. ,* Peter Honey Publications Limited, Berks.
Honey, P. and Mumford, A. (2000b) *The Learning Styles Questionnaire 80-item version.,* Peter Honey Publications Limited, Berks.
Hopewell, S., Dutton, S., Yu, L. M., Chan, A. W. and Altman, D. G. (2010) *BMJ,* **340,** c723.

Houben, R. M., Vlaeyen, J. W., Peters, M., Ostelo, R. W., Wolters, P. M. and Stomp-van den Berg, S. G. (2004) *Clin J Pain,* **20,** 37-44.
Houben, R. M. A., Ostelo, R. W. J. G., Vlaeyen, J. W. S., Wolters, P. M. J. C., Peters, M. and Stomp-van den Berg, S. G. M. (2005) *European Journal of Pain,* **9,** 173-183.
Howlin, P., Gordon, R. K., Pasco, G., Wade, A. and Charman, T. (2007) *Journal of Child Psychology & Psychiatry,* **48,** 473-481.
Hudak, P. L. and Wright, J. G. (2000) *Spine (Phila Pa 1976),* **25,** 3167-77.
Hughes, J. P. (2007) In *Wiley Encyclopedia of Clinical Trials*John Wiley & Sons, Inc.
Hussey, M. A. and Hughes, J. P. (2007) *Contemporary Clinical Trials,* **28,** 182-191.
IFOMPT (2008) IFOMT.
Jellema, P., van der Windt, D. A., van der Horst, H. E., Blankenstein, A. H., Bouter, L. M. and Stalman, W. A. (2005a) *Pain,* **118,** 350-9.
Jellema, P., van der Windt, D. A., van der Horst, H. E., Twisk, J. W., Stalman, W. A. and Bouter, L. M. (2005b) *BMJ,* **331,** 84.
Jenkinson, C., Gray, A., Doll, H., Lawrence, K., Keoghane, S. and Layte, R. (1997) *Medical Care,* **35,** 1109-1118.
Jensen, G., Resnik, L. and Haddad, A. (2008) In *Clinical Reasoning in the Health Professions*, Vol. 1 (Eds, Higgs, J., Jones, M. A., Loftus, S. and Christensen, N.) Butterworth-Heinemann.
Jensen, G. M., Gwyer, J., Hack, L. M. and Shepard, K. F. (2007a) In *Expertise in Physical Therapy Practice (Second Edition)*W.B. Saunders, Saint Louis, pp. 145-173.
Jensen, G. M., Gwyer, J., Hack, L. M. and Shepard, K. F. (2007b) In *Expertise in Physical Therapy Practice (Second Edition)*W.B. Saunders, Saint Louis, pp. 61-61.
Jensen, G. M., Gwyer, J., Hack, L. M. and Shepard, K. F. (2007c) In *Expertise in Physical Therapy Practice (Second Edition)*W.B. Saunders, Saint Louis, pp. 19-47.
Jensen, G. M., Gwyer, J. and Shepard, K. F. (2000) *Phys Ther,* **80,** 28-43; discussion 44-52.
Jette, A. M. (1993) *Phys Ther,* **73,** 528-37.
Jette, A. M. (1995) *Phys Ther,* **75,** 965-70.
Jette, A. M. (2005) *Phys Ther,* **85,** 118-9.
Jette, A. M. (2006) *Phys Ther,* **86,** 726-34.
Jette, A. M. (2009) *J Gerontol A Biol Sci Med Sci,* **64,** 1165-8.
Jette, A. M. (2010) *Phys Ther,* **90,** 1064-5; author reply 1066-7.
Jette, A. M. and Haley, S. M. (2005) *Journal of Rehabilitation Medicine,* **37,** 339-345.
Jette, A. M. and Latham, N. (2010) *Phys Ther,* **90,** 324-5.
Jette, A. M., Norweg, A. and Haley, S. M. (2008) *Disabil Rehabil,* **30,** 963-9.
Johns, C. (2010) John Wiley & Sons, Ltd. (UK), pp. 318p.
Johnston, V. (2009) *Aust J Physiother,* **55,** 141.
Jones, B. and Kenward, M. G. (2003) *Design and Analysis of Cross-Over Trials,* Chapman and Hall, London.
Jones, M. A., Jensen, G. and Edwards, I. (2008) In *Clinical Reasoning in the Health Professions*(Eds, Higgs, J., Jones, M. A., Loftus, S. and Christensen, N.) Butterworth-Heinemann.
Jones, M. A. and Rivett, D. A. (2004) *Clinical Reasoning for Manual Therapists,* Butterworth-Heinemann, Edinburgh.
Kalet, A. L., Gillespie, C. C., Schwartz, M. D., Holmboe, E. S., Ark, T. K., Jay, M., Paik, S., Truncali, A., Hyland Bruno, J., Zabar, S. R. and Gourevitch, M. N. (2010) *Academic Medicine: Journal Of The Association Of American Medical Colleges,* **85,** 844-851.
Keen, J. (2006) In *Qualitative Research in Health Care*(Eds, Pope, C. and Mays, N.) Blackwell, Oxford.
Kelly, S. P. (2007) *Journal of Physical Therapy Education,* **21,** 63-69.
Kitzinger, J. (1995) *BMJ (Clinical Research Ed.),* **311,** 299-302.
Kitzinger, J. (2006) *Focus Groups,* Blackwell Publishing Ltd.
Koles, P., Nelson, S., Stolfi, A., Parmelee, D. and Destephen, D. (2005) *Med Educ,* **39,** 1045-55.
Lam, W. W., Fielding, R., Johnston, J. M., Tin, K. Y. and Leung, G. M. (2004) *Med Educ,* **38,** 987-97.
Lewin, S., Glenton, C. and Oxman, A. D. (2009) *BMJ (Clinical Research Ed.),* **339,** b3496-b3496.
Lin, C. W., McAuley, J. H., Macedo, L., Barnett, D. C., Smeets, R. J. and Verbunt, J. A. (2011) *Pain (03043959),* **152,** 607-613.
Lincoln, Y. S. and Guba, E. G. (1985) *Naturalistic Inquiry,* Sage, Beverly Hills.
Lingard, L., Albert, M. and Levinson, W. (2008) *BMJ,* **337,** a567.

Linton, S. J. and Boersma, K. (2003) *Clin J Pain,* **19,** 80-6.
Loftus, S. and Smith, M. (2008) In *Clinical Reasoning in the Health Professions*(Eds, Higgs, J., Jones, M. A., Loftus, S. and Christensen, N.) Butterworth-Heinemann.
Lundh, A. and Gotzsche, P. C. (2008) *BMC Med Res Methodol,* **8,** 22.
Magraw, R. M., Fox, D. M. and Weston, J. L. (1978) *Journal Of Medical Education,* **53,** 539-546.
Maher, C. G. and Grotle, M. (2009) *Clin J Pain,* **25,** 666-70.
Main, C. J., Sullivan, M. J. L. and Watson, P. J. (2007) *Pain Management: Practical applications of the biopsychosocial perspective in clinical and occupational settings,* Churchill Livingstone, Edinburgh.
Margison, D. A. and French, D. J. (2007) *J Occup Environ Med,* **49,** 59-67.
Mdege, N. D., Man, M. S., Taylor Nee Brown, C. A. and Torgerson, D. J. (2011) *J Clin Epidemiol,* **64,** 936-48.
Mertens, D. M. (2009) *Research and Evaluation in Education and Psychology: Integrating Diversity With Quantitative, Qualitative, and Mixed Methods,* Sage, Los Angeles.
Miles, M. B. and Huberman, A. M. (1994) *Qualitative data analysis: A sourcebook of new methods,* Sage, Thousand Oaks.
Mitchell, L. (2008) *Advances in Physiotherapy,* **10,** 119-126.
Morgan, D. L. (1996) *Annual Reviews Sociology,* **22,** 129-152.
Morgan, D. L. (2007) *Journal of Mixed Methods Research,* **1,** 48-76.
Morgan, D. L. (2010) *Qual Health Res,* **20,** 718-22.
Mourad, O. and Redelmeier, D. A. (2006) *Med Educ,* **40,** 637-44.
Murphy, E., Dingwall, R., Greatbatch, D., Parker, S. and Watson, P. (1998) *Health Technol Assess,* **2,** iii-ix, 1-274.
Murray, E., Jolly, B. and Modell, M. (1997a) *BMJ,* **315,** 920-3.
Murray, E., Todd, C. and Modell, M. (1997b) *Med Educ,* **31,** 369-74.
Nachemson, A. L., Waddell, G. and Nordlund, A. I. (2000) In *Neck and back pain. The scientific evidence of causes, diagnosis and treatment.*(Eds, Nachemson, A. L. and Jonsson, E.) Lippincott Williams & Wilkins, Philadelphia, pp. 165-187.
Newman, I. and Benz, C. R. (1998) *Qualitative-quantitative research methodology: Exploring the interactive continuum.,* Southern Illinois University Press, Carbondale and Edwardsville.
Ni Mhurchu, C., Turley, M., Gorton, D., Jiang, Y., Michie, J., Maddison, R. and Hattie, J. (2010) *BMC Public Health,* **10,** 738-738.
Nieuwboer, A., Kwakkel, G., Rochester, L., Jones, D., van Wegen, E., Willems, A. M., Chavret, F., Hetherington, V., Baker, K. and Lim, I. (2007) *J Neurol Neurosurg Psychiatry,* **78,** 134-40.
NRES (2009) National Research Ethics Service / National Patient Safety Agency.
O'Cathain, A., Murphy, E. and Nicholl, J. (2008) *J Health Serv Res Policy,* **13,** 92-8.
O'Cathain, A., Murphy, E. and Nicholl, J. (2010) *BMJ,* **341,** c4587.
Ostelo, R. W., Stomp-van den Berg, S. G., Vlaeyen, J. W., Wolters, P. M. and de Vet, H. C. (2003) *Man Ther,* **8,** 214-22.
Overmeer, T., Boersma, K., Denison, E. and Linton, S. J. (2011) *Phys Ther,* **91,** 804-19.
Overmeer, T., Boersma, K., Main, C. J. and Linton, S. J. (2009) *J Eval Clin Pract,* **15,** 724-32.
Patton, M. Q. (1990) *Qualitative research and evaluation methods,* Sage, Thousand Oaks, CA.
Patton, M. Q. (2002) *Qualitative research and evaluation methods.,* Sage, Thousand Oaks, CA.
Pengel, L. H. M., Refshauge, K. M. and Maher, C. G. (2004) *Spine,* **29,** 879-883.
Petty, N. J. and Morley, M. (2009) *Man Ther,* **14,** 461-2.
Petty, N. J., Scholes, J. and Ellis, L. (2011a) *Man Ther,* **16,** 590-5.
Petty, N. J., Scholes, J. and Ellis, L. (2011b) *Physiotherapy,* **97,** 218-25.
Pietrobon, R., Coeytaux, R. R., Carey, T. S., Richardson, W. J. and DeVellis, R. F. (2002) *Spine,* **27,** 515-522.
Plack, M. M. (2008) *Journal of Physical Therapy Education,* **22,** 7-18.
Polsky, D., Willke, R. J., Scott, K., Schulman, K. A. and Glick, H. A. (2001) *Health Economics,* **10,** 27-37.
Pope, C. and Mays, N. (2006) *Observational Methods,* Blackwell Publishing Ltd.
Prystowsky, J. B. and Bordage, G. (2001) *Med Educ,* **35,** 331-6.
Purtilo, R. (2007) In *Expertise in Physical Therapy Practice (Second Edition)*(Eds, Jensen, G. M., Gwyer, J., Hack, L. M. and Shepard, K. F.) W.B. Saunders, Saint Louis, pp. xi-xiii.
Rabin, R. and de Charro, F. (2001) *Annals Of Medicine,* **33,** 337-343.
Rainville, J., Bagnall, D. and Phalen, L. (1995) *The Clinical Journal of Pain,* **11,** 287-295.

Rainville, J., Carlson, N., Polatin, P., Gatchel, R. J. and Indahl, A. (2000) *Spine (Phila Pa 1976),* **25,** 2210-20.
Randomization.com (2008).
Rawlins, L., Woollard, M., Williams, J. and Hallam, P. (2009) *BMJ,* **339,** b4707.
Reid, S., Haugh, L. D., Hazard, R. G. and Tripathi, M. (1997) *Journal of Occupational Rehabilitation,* **7,** 1-14.
Resnik, L. (2007) In *Expertise in Physical Therapy Practice*(Eds, Jensen, G., Gwyer, J., Hack, L. M. and Shepard, K. F.) Saunders, St Louis.
Resnik, L. and Dobrykowski, E. (2005) *Orthop Nurs,* **24,** 14-24.
Resnik, L. and Dobrzykowski, E. (2003) *J Orthop Sports Phys Ther,* **33,** 307-16; discussion 317-8.
Resnik, L. and Jensen, G. M. (2003) *Phys Ther,* **83,** 1090-106.
Richardson, S., Dohrenwend, B. and Klein, D. (1965) *Interviewing: its forms and functions.,* Basic Books, New York.
Rikers, R. M., Loyens, S., te Winkel, W., Schmidt, H. G. and Sins, P. H. (2005) *Acad Med,* **80,** 945-9.
Rossman, G. B. and Rallis, S. F. (2003) *Learning in the field: An introduction to qualitative research,* Sage, Thousand Oaks, CA.
Rossman, G. B. and Wilson, B. L. (1985) *Evaluation Review,* **9,** 627-643.
Rothstein, J. (1999) In *Expertise in physical therapy practice.*(Eds, Jensen, G. M., Gwyer, J., Hack, L. M. and Shepard, K. F.) Butterworth-Heinemann, Boston.
Rushton, A. and Lindsay, G. (2007a) *International Journal of Therapy & Rehabilitation,* **14,** 156-161.
Rushton, A. and Lindsay, G. (2007b) *International Journal of Therapy & Rehabilitation,* **14,** 252-258.
Rushton, A. and Lindsay, G. (2008) *Med Teach,* **30,** e100-7.
Rushton, A. and Lindsay, G. (2010) *Manual Therapy,* **15,** 93-99.
Rushton, A. and Moore, A. (2009) *Manual Therapy,* **In Press, Corrected Proof**.
Schnelle, J. F., Newman, D. R., White, M., Volner, T. R., Burnett, J., Cronqvist, A. and Ory, M. (1992) *J Am Geriatr Soc,* **40,** 381-5.
Schön, D. (1983) *The reflective practitioner: how professionals think in action.,* Temple Smith, London.
Schulz, K. F., Chalmers, I. and Altman, D. G. (2002) *Ann Intern Med,* **136,** 254-9.
Schulz, K. F. and Grimes, D. A. (2002) *Lancet,* **359,** 696-700.
Scott, G. (2004) *Nursing Standard,* **19,** 6-6.
Seale, J. K. and Barnard, S. (1999) *British Journal of Occupational Therapy,* **62,** 371-375.
Shepard, K. F., Hack, L. M., Gwyer, J. and Jensen, G. M. (1999) *Qual Health Res,* **9,** 746-58.
Silverman, D. (2006) *Interpreting Qualitative Data,* Sage, London.
Silverman, D. (2010) *Doing Qualitative Research,* Sage, Los Angeles.
Sim, J. (1996) *Man Ther,* **1,** 104-6.
Sim, J. (1997) *Physiother Res Int,* **2,** 7-11.
Sim, J. (2004) In *Physiotherapy - a psychosocial approach*(Eds, French, S. and Sim, J.) Elsevier, London.
Sim, J. and Wright, C. (2000) *Research in Health Care - Concepts, Designs and Methods,* Nelson Thornes, Cheltenham.
Sitzia, J. (1999) *Int J Qual Health Care,* **11,** 319-28.
Sterling, M. and Brentnall, D. (2007) *Aust J Physiother,* **53,** 65.
Stevenson, K., Lewis, M. and Hay, E. (2006) *J Eval Clin Pract,* **12,** 365-75.
Stewart, M., Maher, C. G., Refshauge, K. M., Bogduk, N. and Nicholas, M. (2007) *Spine,* **32,** 580-585.
Stolberg, H. O., Norman, G. and Trop, I. (2004) *AJR Am J Roentgenol,* **183,** 1539-44.
Stratford, P., Gill, C., Westaway, M. and Binkley, J. (1995) *Physiotherapv Canada,* **47,** 258-263.
Tashakkori, A. and Teddlie, C. (1998) *Mixed Methodology: Combining qualitative and quantitative approaches,* Sage, Thousand Oaks, CA.
Tashakkori, A. and Teddlie, C. (2010) *Sage Handbook of Mixed Methods in Social and Behavioural Research,* Sage, Thousand Oaks, CA.
Taylor, S., Bell, E., Grugulis, I. and Storey, J. (2007) Open University Business School, London.
WCPT (2011) World Confederation of Physical Therapy.
Wendler, M. C. (2001) *J Adv Nurs,* **35,** 521-5.
Westaway, M. D., Stratford, P. W. and Binkley, J. M. (1998) *Journal of Orthopaedic & Sports Physical Therapy,* **27,** 331-338.
Westman, A., Linton, S. J., Ohrvik, J., Wahlen, P. and Leppert, J. (2008) *Eur J Pain,* **12,** 641-9.
Whitcomb, M. E. (2002a) *Acad Med,* **77,** 359-60.

Whitcomb, M. E. (2002b) *Acad Med,* **77,** 1067-8.
Whiteford, G. and Wright St Clair, V. (Eds.) (2005) *Occupation & Practice in Context: Professional, Sociolcultural and Political Perspectives,* Elsevier, Sydney.
Young, I. A., Cleland, J. A., Michener, L. A. and Brown, C. (2010) *American Journal of Physical Medicine & Rehabilitation,* **89,** 831-839.
Young, T. A. (2005) *Pharmacoeconomics,* **23,** 1229-42.

Appendix A: Quantitative Data Collection Tools

Clinical Reasoning Training in Physiotherapy - Patient Outcome Questionnaire: Baseline		
Therapist ID<br>…………………	Cohort Number<br>…………………	Patient Number<br>……………………

INFORMATION FOR PARTICIPANTS

Thank you for agreeing to be part of this study, looking at how effective physiotherapy training programmes are at achieving better outcomes and results for patients.

This questionnaire has been designed to help us evaluate how your pain / musculoskeletal problem is affecting you, and how effective your physiotherapy treatment is at returning you to normal activity. To do this, we will also be asking you to complete a questionnaire when your treatment is completed and then again twelve months from that date. We are very grateful your assistance in completing these subsequent questionnaires as well.

We would be very grateful if you would complete ALL questions in each section of the questionnaire.

We have tried to avoid any repetitive questions; however, in section C some information may appear to be repetitive, because the questions are part of validated instruments and therefore cannot be deleted, as this would invalidate that particular section of the questionnaire. Please therefore ensure that you answer ALL questions.

The research team will not have access to any of your details and your responses will remain confidential. The patient ID number is in order for us to be able to check that you have responded and to match your responses from the next questionnaire. Your name and address will not be made known to the research team.

Section A: Personal and Work Information:

This information helps us to understand your background and how this problem is affecting your work.

Sex:

M ☐ F ☐

Date of birth: ……. /……. /……..
Day Month Year

How long have you had musculoskeletal pain?

…………Years ………Months

Research ID:
(physio use only)

To which of these ethnic groups do you consider you belong (✓)?

White
British ◻ Other White background ◻

Asian or Asian British
Bangladeshi ◻ Indian ◻ Pakistani ◻ Other Asian background ◻

Black or Black British
Caribbean ◻ African ◻ Other Black background ◻

Chinese or other ethnic group
Chinese ◻ Other ◻

Mixed
White / Black Caribbean ◻ White / Black African ◻ White / Asian ◻
Other Mixed Background ◻

Work Status:

Employed ☐

Self-employed ☐

Unemployed ☐

Carer ☐

Retired ☐

Student ☐

Please could you indicate (✓) which income band your approximate annual household income would fall within:

Less than £10,000		£4,000 - £49,000	
£10,00 - £19,000		£50,000 - £59,000	
£2,000 - £29,000		£60,000 - £69,000	
£30,000 £39000		£70,000 or above	

Please choose a statement below that best describes your main work situation over the last 6 months. <u>Tick only one box</u>.

☐ I have been working my usual hours and duties

☐ I am working my usual hours, but I have been unable to do some/all of my usual duties because of my pain / musculoskeletal condition.

What proportion of your duties have you stopped doing? Please state a percentage:………%

☐ I am doing all my usual duties, but I have been unable to work my usual hours because of my pain / musculoskeletal condition.

How many hours less have you worked per week? ……… hours

☐ I am currently unable to work because of ***this*** problem.

☐ I am currently unable to work because of ***another*** problem.

Sick Pay / Benefits:

Are you currently receiving any of the following because of this problem:

Employer's sick pay Yes ☐ No ☐

Statutory sick pay Yes ☐ No ☐

Disability living allowance Yes ☐ No ☐

Section B: Healthcare Information:
This section tells us which health services and medication you have used for your pain/musculoskeletal condition

1. During the last 6 months, have you been to see/made contact with any other health professional or organisation for your pain / musculoskeletal condition? (Please include both NHS and private opinions.)

Yes □ (please complete the tables below)
No □ (continue with question 2)

**NHS Consultations**			
Health care professional	Number of visits made to this person over the past 6 months	Number of telephone consultations with this person over the past 6 months	Number of visits by this person to your home over the past 6 months
A & E			
Counsellor			
Doctor (GP)			
Hospital Consultant			
NHS Direct			
Physiotherapist			
Practice nurse			
Other (please specify)			

**Private Consultations**		
Health care professional	Number of visits made to this person over the past 6 months	Costs of visits
Acupuncturist		
Chiropractor		
Hospital Consultant		
Osteopath		
Physiotherapist		
Other (please specify)		

2. During the last 6 months, have you received any diagnostic tests, investigations (e.g. blood test, X rays, MRI scan) because of your pain/ musculoskeletal condition?

Yes □ (please complete the table below)
No □ (continue with question 3)

Date (if known)	Type of treatment/investigation

3. During the last 6 months have you been admitted to hospital (as an in-patient or out-patient) because of your pain/ musculoskeletal condition?

Yes □ (please complete the table below)
No □ (continue with question 4)

Date (if known)	Inpatient Location	Number of days in hospital	Outpatient attendance

4. During the last 6 months have you been prescribed any medication for your pain/ musculoskeletal condition?

Yes □ (please complete the table below)
No □ (continue with question 5)

Name of medicine	Number of tablets to be taken per day	Strength of each tablet	Number of days supplied
e.g. Ibuprofen	*e.g. 6*	*e.g. 200mg*	*e.g. 7*

5. During the last 6 months, have you bought any over-the-counter medicines, other treatments, or equipment to help treat your pain/ musculoskeletal condition?

Yes □ (please complete the table below)
No □ (please continue with Section D)
(Please include painkillers, anti-inflammatory drugs/gels/creams/sprays etc; herbal or complementary remedies including massage; and equipment e.g. TENS machine)

Item or other remedy bought	Total cost (£)

Section C: Me and My Life.

Functional Scale: Your physiotherapist will complete this with you to identify up to three important activities that you are unable to do or are having difficulty with as a result of your problem. Your physiotherapist will ask you some questions to help you rate how difficult those activities are.

Activity	Initial Score:	Current Score:
1.		
2.		
3.		

Örebro Musculoskeletal Pain Questionnaire.

Please read and answer questions carefully. Do not take too long to answer the questions; however it is important that you answer every question. There is **always** a response for your particular situation.

1. Where do you have pain? Place a tick (✓) for all appropriate sites.

☐ Neck ☐ Shoulder ☐ Arm ☐ Upper Back

☐ Lower Back ☐ Leg ☐ Other (state) _____________

2. How many days of work have you missed because of pain during the past 18 months? Tick (✓) one

☐ 0 days (1) ☐ 1-2 days (2) ☐ 3-7 days (3) ☐ 8-14 days (4)

☐ 15-30 days (5) ☐ 1 month (6) ☐ 2 months (7) ☐ 3-6 months (8)

☐ 6-12 months (9) ☐ over 1 year (10)

3. How long have you had your current pain problem? Tick (✓) one.

☐ 0-1 weeks (1) ☐ 1-2 weeks (2) ☐ 3-4 weeks (3) ☐ 4-5 weeks (4)

☐ 6-8 weeks (5) ☐ 9-11 weeks (6) ☐ 3-6 months (7) ☐ 6-9 months (8)

☐ 9-12 months (9) ☐ over 1 year (10)

4. Is your work heavy or monotonous? Circle the best alternative.

0 1 2 3 4 5 6 7 8 9 10

Not at all Extremely

5. How would you rate the pain that you have had during the past week? Circle one.

0 1 2 3 4 5 6 7 8 9 10

No pain Pain as bad as it could be

6. In the past three months, on average, how bad was your pain on a 0-10 scale? Circle one.

0 1 2 3 4 5 6 7 8 9 10

No pain Pain as bad as it could be

7. How often would you say that you have experienced pain episodes, on average, during the past three months? Circle one.

0 1 2 3 4 5 6 7 8 9 10

Never Always

8. Based on all things you do to cope, or deal with your pain, on an average day, how much are you able to decrease it? Circle the appropriate number.

0 1 2 3 4 5 6 7 8 9 10

Can't decrease it at all Can decrease it completely

9. How tense or anxious have you felt in the past week? Circle one.

0 1 2 3 4 5 6 7 8 9 10

Absolutely calm and relaxed As tense and anxious as I've ever felt

10. How much have you been bothered by feeling depressed in the past week? Circle one.

0 1 2 3 4 5 6 7 8 9 10

Not at all Extremely

11. In your view, how large is the risk that your current pain may become persistent? Circle one.

0 1 2 3 4 5 6 7 8 9 10

No risk Very large risk

12. In your estimation, what are the chances that you will be able to work in six months? Circle one.

0 1 2 3 4 5 6 7 8 9 10

No chance Very large chance

13. If you take into consideration your work routines, management, salary, promotion possibilities and work mates, how satisfied are you with your job? Circle one.

0 1 2 3 4 5 6 7 8 9 10

Not satisfied at all Completely satisfied

Here are some of the things that other people have told us about their pain. For each statement, circle one number from 0 to 10 to say how much physical activities, such as bending, lifting, walking or driving, would affect your pain.

14. Physical activity makes my pain worse.

0 1 2 3 4 5 6 7 8 9 10

Completely disagree — Completely agree

15. An increase in pain is an indication that I should stop what I'm doing until the pain decreases.

0 1 2 3 4 5 6 7 8 9 10

Completely disagree — Completely agree

16. I should not do my normal work with my present pain.

0 1 2 3 4 5 6 7 8 9 10

Completely disagree — Completely agree

Here is a list of five activities. Circle the one number that best describes your current ability to participate in each of these activities.

17. I can do light work for an hour.

0 1 2 3 4 5 6 7 8 9 10

Can't do it because of pain problem — Can do it without pain being a problem

18. I can walk for an hour.

0 1 2 3 4 5 6 7 8 9 10

Can't do it because of pain problem — Can do it without pain being a problem

19. I can do ordinary household chores.

0 1 2 3 4 5 6 7 8 9 10

Can't do it because of pain problem — Can do it without pain being a problem

20. I can do the weekly shopping.

0 1 2 3 4 5 6 7 8 9 10

Can't do it because of pain problem — Can do it without pain being a problem

21. I can sleep at night.

Can't do it because of pain problem — 0 1 2 3 4 5 6 7 8 9 10 — Can do it without pain being a problem

<u>**Me and My Health**</u>

INSTRUCTIONS: For each statement below please mark an X in the box to the right that best describes how much you disagree—agree with the statement as it applies to you personally.

There are no right or wrong answers. Your answers should be what are true to you and not just what you think the physiotherapist wants you to say.

	Strongly Disagree	Disagree	Agree	Strongly Agree
**1 When all is said and done, I am the person who is responsible for managing my health condition.**	☐	☐	☐	☐
**2 Taking an active role in my own health care is the most important factor in determining my health and ability to function.**	☐	☐	☐	☐
**3 I am confident that I can take actions that will help prevent or minimize some symptoms or problems associated with my health condition.**	☐	☐	☐	☐
**4 I know what each of my prescribed medications do.**	☐	☐	☐	☐
**5 I am confident that I can tell when I need to go get medical care and when I can handle a health problem myself.**	☐	☐	☐	☐
**6 I am confident that I can tell doctor concerns I have even when he or she does not ask.**	☐	☐	☐	☐
**7 I am confident that I can follow through on medical treatments I need to do at home.**	☐	☐	☐	☐
**8 I understand the nature and causes of my health condition(s).**	☐	☐	☐	☐
**9 I know the different medical treatment options available for my health condition.**	☐	☐	☐	☐
**10 I have been able to maintain the lifestyle changes for my health condition that I have made.**	☐	☐	☐	☐
**11 I know how to prevent further problems with my health condition.**	☐	☐	☐	☐
**12 I know how to prevent further problems when new situations or problems arise with my health condition.**	☐	☐	☐	☐
**13 I am confident that I can maintain lifestyle changes, like diet and exercise, even during times of stress.**	☐	☐	☐	☐

Me and My Quality of Life.

By placing a tick in one box in each group below, please indicate which statements best describe your own health state today.

Mobility

I have no problems in walking about ❑

I have some problems in walking about ❑

I am confined to bed ❑

Self-Care

I have no problems with self-care ❑

I have some problems washing or dressing myself ❑

I am unable to wash or dress myself ❑

Usual Activities (*e.g. work, study, housework, family or leisure activities)*

I have no problems with performing my usual activities ❑

I have some problems with performing my usual activities ❑

I am unable to perform my usual activities ❑

Pain/Discomfort

I have no pain or discomfort ❑

I have moderate pain or discomfort ❑

I have extreme pain or discomfort ❑

Anxiety/Depression

I am not anxious or depressed ❑

I am moderately anxious or depressed ❑

I am extremely anxious or depressed ❑

© EQ-5D

To help people say how good or bad a health state is, we have drawn a scale (rather like a thermometer) on which the best state you can imagine is marked 100 and the worst state you can imagine is marked 0.

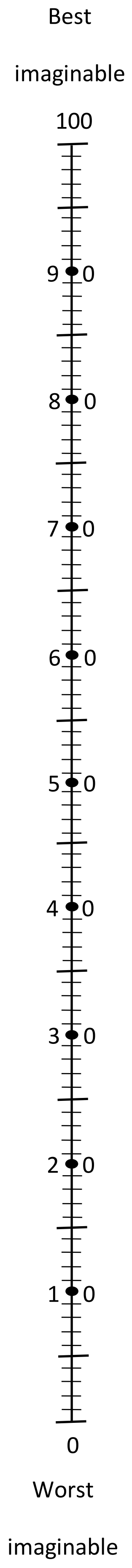

© EQ-5D

Me and My Physiotherapy.

Satisfaction questionnaire: How satisfied have you been with your physiotherapy treatment for your pain/musculoskeletal condition?

**Please answer the questions below by circling the response which best describes your opinions about your treatment.**					
	**Strongly Disagree**	**Disagree**	**Neutral**	**Agree**	**Strongly Agree**
**1 The department receptionist was courteous.**	1	2	3	4	5
**2 The registration process was not appropriate.**	1	2	3	4	5
**3 The waiting area was comfortable (in terms of lighting, temperature, decor and furnishings).**	1	2	3	4	5
**4 My therapist did not spend enough time with me.**	1	2	3	4	5
**5 My therapist thoroughly explained the treatment(s) I received.**	1	2	3	4	5
**6 My therapist treated me respectfully.**	1	2	3	4	5
**7 My therapist listened to my concerns.**	1	2	3	4	5
**8 My therapist did not answer all my questions.**	1	2	3	4	5
**9 My therapist advised me on ways to avoid future problems.**	1	2	3	4	5
**10 My therapist gave me detailed instructions regarding my home exercise programme.**	1	2	3	4	5
**11 Overall, I am completely satisfied with the services I received from my therapist.**	1	2	3	4	5
**12 I would return to this department for future services or care.**	1	2	3	4	5

Thank you for being willing to participate in this research project and taking the time to complete this questionnaire.

Appendix B – Patient Information Sheet and Consent Form

Bwrdd Iechyd Prifysgol Caerdydd a'r Fro
Cardiff and Vale University Health Board

Patient Information Sheet: version 1

The Effect of Work-Based Facilitation in Clinical Reasoning on Outcome in Musculo-skeletal Physiotherapy

We would like to invite you to take part in our research study. Before you decide we would like you to understand why the research is being done and what it would involve for you. **The researcher will go through the information sheet with you and answer any questions you have.** Talk to others about the study if you wish. (Part 1 tells you the purpose of this study and what will happen to you if you take part. Part 2 gives you more detailed information about the conduct of the study). Ask us if there is anything that is not clear.

PART 1

WHAT IS THE PURPOSE OF THE STUDY?

The aim of this study is to evaluate how effective a training programme for physiotherapists in the workplace is at equipping physiotherapists to achieve better results for their patients. This will allow us to see whether there are any potential benefits to using such a training programme on a larger scale, and whether it is affordable.

WHY HAVE I BEEN INVITED TO TAKE PART?

It is the physiotherapists in the department who are the main focus of the research. While we are looking at whether the training they receive improves their performance, we feel that the best measure of any progress is the results that patients achieve. We would like you to provide us with that information.

DO I HAVE TO TAKE PART?

It is up to you to decide to join the study. We will describe the study and go through this information sheet. If you agree to take part, we will then ask you to sign a consent form. You are free to withdraw at any time, without giving a reason. This would not affect the standard of care you receive.

WHAT WILL HAPPEN TO ME IF I TAKE PART?

You will receive your normal treatment. Your physiotherapist will collect information about your problem at the start and end of treatment, and you will be required to complete some questionnaires as part of this. The differences between your questionnaire scores at the start and end of treatment will help us to measure how much progress you have made with physiotherapy. This is all part of standard

practice in physiotherapy at Cardiff and Vale University Health Board. What is different is that you will be asked to complete two additional questionnaires, and also be contacted a year after your treatment, in order to repeat these questionnaires, so that we can measure the longer term effect of your physiotherapy. You will not be required to meet with the researchers or attend additional clinics. The research team will not have access to any of your details and your responses will remain confidential.

WHAT WILL I HAVE TO DO?

You will need to complete a questionnaire which has 3 sections. The first section contains personal and work information, which helps us to understand your background and how this problem is affecting your work. The second section contains healthcare information, which tells us which health services and medication you have used for your pain/musculoskeletal condition. The third section contains questions that are measurement tools for musculoskeletal problems, and help us to measure your progress with treatment, and how satisfied you are with your physiotherapy. We have tried to avoid any repetitive questions; however, in the third section some information may appear to be repetitive, because the questions are part of validated instruments and therefore cannot be deleted, as this would invalidate that particular section of the questionnaire.

WHAT ARE THE POSSIBLE RISKS OF TAKING PART?

You will be receiving your normal physiotherapy. There are no risks associated with this study.

WHAT ARE THE POSSIBLE BENEFITS OF TAKING PART?

Ultimately this information will go towards helping the way that we train physiotherapists to become experts. We hope that this will help future patients attending for physiotherapy at Cardiff and Vale University Health Board to have better outcomes with treatment.

WHAT IF THERE IS A PROBLEM?

Any complaint about the way you have been dealt with during the study will be addressed. The detailed information on this is given in Part 2.

If the information in Part 1 has interested you and you are considering participation, please read the additional information in Part 2 before making any decision.

PART 2

WHAT WILL HAPPEN IF I DON’T WANT TO CARRY ON WITH THE STUDY?

You can withdraw from physiotherapy treatment but we would like to keep in contact with you to know your progress. Information collected may still be used.

WHAT IF THERE IS A PROBLEM?

If you have a concern about any aspect of this study, you should ask to speak to the lead researcher who will do his best to answer your questions [telephone: 02920 742629]. If you remain unhappy and wish to complain formally, you can do this through the NHS Complaints Procedure. Details can be obtained from Adrian Broad, Head of Outpatient Physiotherapy Services [telephone: 02920 744263].

CONFIDENTIALITY – WHO WILL KNOW I AM TAKING PART IN THE STUDY?

All information which is collected about you during the course of the research will be kept strictly confidential and stored securely. All of the questionnaires you complete will be coded, and will not contain any personal details, so that you cannot be recognised. This information will be analysed by the lead researcher, Aled Williams. Once the information is processed, the questionnaires will be disposed of securely.

DOES MY GP NEED TO BE INFORMED?

Your GP doesn't need to be notified of your participation in this study as you are receiving your usual physiotherapy. He / she will be notified of your progress with treatment by your physiotherapist as is usual practice.

WHAT WILL HAPPEN TO THE RESULTS OF THE STUDY?

Once the study is completed, participants can obtain a copy of the results from the contact address below. As already stated, the results will go to Swansea University as part of a PhD dissertation. It is possible that in future they could also be published in a scientific medical journal. You will not be able to be identified in any report or publication.

WHO HAS REVIEWED THE STUDY?

All research in the NHS is looked at by independent group of people, called a Research Ethics Committee, to protect your interests. This study has been reviewed and given favourable opinion by the South East Wales Research Ethics Committee.

WHO IS ORGANISING THIS STUDY?

Aled Williams, a Chartered Physiotherapist working at the University Hospital of Wales as a Clinical Specialist in Musculo-skeletal physiotherapy. This study forms part of the PhD at Swansea University which Aled is working towards over the next few years.

CONTACT FOR FURTHER INFORMATION

If you have any further questions about this study please contact Aled Williams on 02920 742625.

Thank you for taking the time to read this information sheet and I look forward to your response in the near future.

Aled Williams, Chartered Physiotherapist 12/03/10

Centre Number:

Study Number:

Patient Identification Number for this trial:

PATIENT CONSENT FORM

Title of Project: *The Effect of Training Physiotherapists in Clinical Reasoning on Patient Outcome*

Name of Researcher: Aled Williams

Please initial box

1. I confirm that I have read and understand the information sheet dated.. (version 1) for the above study. I have had the opportunity to consider the information, ask questions and have had these answered satisfactorily. ☐

2. I understand that my participation is voluntary and that I am free to withdraw at any time without giving any reason, without my medical care or legal rights being affected. ☐

3. I agree to take part in the above study. ☐

______________________________	____________	______________________
Name of Patient	Date	Signature
______________________________	____________	______________________
Name of Person taking consent	Date	Signature

When completed: 1 for patient; 1 for researcher site file; 1 (original) to be kept in medical notes.

Appendix C: Participant Information Sheet & Consent Form

Bwrdd Iechyd Prifysgol Caerdydd a'r Fro
Cardiff and Vale University Health Board

Participant Information Sheet: version 2.0
The Effect of Work-Based Facilitation in Clinical Reasoning on Outcome in Musculo-skeletal Physiotherapy

I would like to invite you to take part in my research study. Before you decide I would like you to understand why the research is being done and what it would involve for you. **I will go through the information sheet with you and answer any questions you have.** Talk to others about the study if you wish. (Part 1 tells you the purpose of this study and what will happen to you if you take part. Part 2 gives you more detailed information about the conduct of the study). Please ask me if anything is not clear.

PART 1

WHAT IS THE PURPOSE OF THE STUDY?
The aim of this study is to evaluate how effective mentoring programmes for physiotherapists in the workplace are at enabling physiotherapists achieve better clinical outcomes for their patients. This will allow me to see whether there are any potential benefits to using such a training programme on a larger scale, and whether it is financially viable.

WHY HAVE I BEEN INVITED TO TAKE PART?
It is the physiotherapists in the departments of Cardiff and Vale University Health Board who are the main focus of the research and in particular their clinical reasoning. Firstly, as clinical reasoning is context-dependent, I wanted to look at staff who work the majority of their hours in the department (rather than in other settings). Secondly, I wanted to take a long term view of patient outcomes, and so wished to look at static staff who would be present for the duration of the study. Finally, the form of training we are looking at is a clinical placement mentoring programme similar to those used on Masters programmes; I wanted to look at staff who have not already undertaken such a placement.

DO I HAVE TO TAKE PART?
It is up to you to decide to join the study. I will describe the study and go through this information sheet. If you agree to take part, I will then ask you to sign a consent form. You are free to withdraw at any time, without giving a reason. This would not affect any future training you receive.

WHAT WILL HAPPEN TO ME IF I TAKE PART?
You will receive your usual training in the department (in-service training, mentoring and techniques sessions) and you will receive a 150 hour "clinical placement" style mentoring programme with a MACP qualified mentor designed to develop your clinical reasoning. This will involve being observed assessing and treating patients, and being given immediate feedback. The discussions will be framed around your clinical reasoning processes. To measure the effect of this mentoring programme, the clinical outcomes of the patients you treat will be collected and compared from 4 groups of patients – one group at the start of the study, and then one at the end of each of three time points, nine months apart (see diagram overleaf). You will undertake the mentoring at the start of one of these time points – which time point you will undertake the mentoring will be decided at random, and by the site you work in. The difference the mentoring process makes to your reasoning performance will also be assessed by an independent assessor from an academic institution who will assess your performance by observing you with a patient at the start, mid-point and end of the study. The assessor's findings will only be shared with you and the

lead researcher. Finally, you and your mentor will be interviewed to investigate your reflections on the clinical supervision programme.

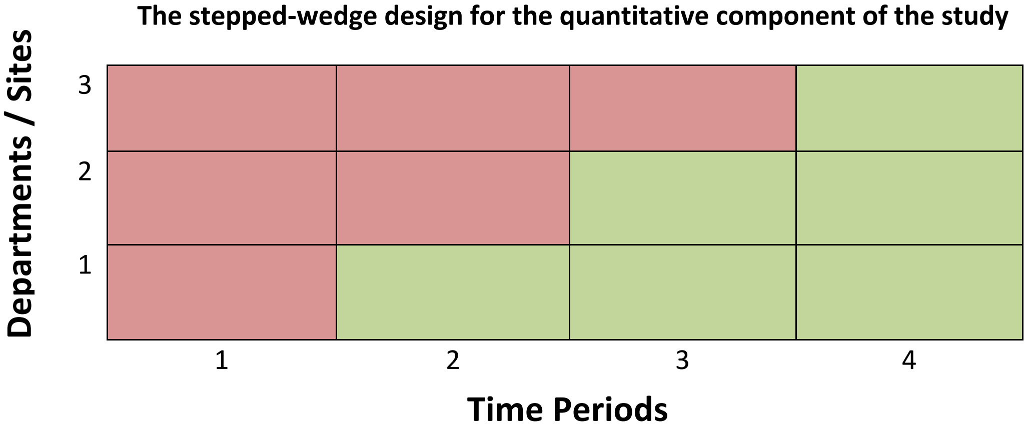

WHAT WILL I HAVE TO DO?

Before the study

You will be required to complete a learning styles questionnaire at the start of the process.

Training during the study

You will be required to attend the health board training programme, and undertake a 150 hour clinical mentoring programme with a MACP clinical mentor observing your practice and discussing your clinical reasoning, with the aim of facilitating your clinical reasoning processes and skills.

Data collection during the study

It is the impact of training on patient outcome that is the primary focus of this study. You will be required to collect patient outcome data at the beginning and end of treatment. While this is usual practice in the physiotherapy service, the range of outcome measures is larger (we are using 3) and we are also collecting demographic and cost utility data. In practice this means that the patient will need to complete a questionnaire containing all this data at first assessment, discharge, and then 12 months post discharge (collected by telephone contact). This data collection will be for 10 consecutive consenting patients in four cohorts. The first cohort will be at the start of the study, the second, third and fourth will be at the end of each 9 month time period. This will allow us to see if the training is leading to greater improvements in validated patient outcome measures.

At the end of the study

You will be interviewed for your perspective by the lead researcher to add qualitative data to the quantitative patient outcome data.

WHAT ARE THE POSSIBLE RISKS OF TAKING PART?

You will be receiving your usual training, and a recognised mentoring programme. There are no risks associated with this study.

WHAT ARE THE POSSIBLE BENEFITS OF TAKING PART?

You will have the opportunity to be mentored by a MACP qualified clinical mentoring with the specific aim of developing your clinical reasoning skills in order to achieve better outcomes with your patients. The potential benefits are, therefore, improved clinical reasoning skills, and better patient outcomes.

WHAT IF THERE IS A PROBLEM?

Any complaint about the way you have been dealt with during the study will be addressed. The detailed information on this is given in Part 2.

If the information in Part 1 has interested you and you are considering participation, please read the additional information in Part 2 before making any decision.

PART 2
WHAT DATA WILL I BE REQUIRED TO COLLECT AS PART OF THIS STUDY?
Outcome data for each patient will be collected at initial assessment and discharge, as is current protocol at Cardiff and Vale University Health Board. The outcomes chosen for this study are:

- *Patient Specific Functional Scales (PSFS)* will be used for the patients' most disabling functional problems.
- Health related quality of life will be measured using the *EQ-5D*.
- *Numerical Rating Scores (NRS)* will be taken as part of the *Örebro Musculoskeletal Pain Questionnaire,* which will also give valuable data on yellow flags which might affect patient outcome.
- *Patient Activation Measure (PAM)* will be taken to evaluate how adept the patient is at self managing their problem.

To ascertain the cost-effectiveness of the training programme you will also be required to collect additional data:

- Referral on for secondary care opinion or intervention, or for diagnostic imaging.
- Use of prescription medication.
- Number and type of attendances
- Return to work

Each patient will be contacted by you 12 months post discharge and this data will be collected again, in order to ascertain the long term outcome of physiotherapy.

WHAT WILL HAPPEN IF I DON'T WANT TO CARRY ON WITH THE STUDY?
Your participation is voluntary and you are free to withdraw at any time without giving any reason, without your usual training or professional or legal rights being affected. Information collected may still be used.

WHAT IF THERE IS A PROBLEM?
If you have a concern about any aspect of this study, you should ask to speak to the lead researcher who will do his best to answer your questions [telephone: 02920 742629]. If you remain unhappy and wish to complain formally, you can do this by contacting Adrian Broad, Head of Outpatient Physiotherapy Services [telephone: 02920 744263].

CONFIDENTIALITY – WHO WILL KNOW I AM TAKING PART IN THE STUDY?
All information which is collected about you during the course of the research will be kept strictly confidential and stored securely. Your personal details will not be used as you will be allocated a code which will be a unique identifier to link you to your patient outcomes. The key which correlates therapists to codes will not be kept on NHS computers, and stored securely so that your outcomes will remain anonymous in the workplace. All of the outcome questionnaires your patients complete will be coded, so that you cannot be recognised. The results of your independent assessment will be fed back directly to you, and also coded so that you cannot be recognised. Information from interviews will be recorded, transcribed and analysed by the lead researcher, Aled Williams. Once the information is processed, the recordings will be disposed of securely.

WHAT WILL HAPPEN TO THE RESULTS OF THE STUDY?
Once the study is completed, participants can obtain a copy of the results from the contact address below. As already stated, the results will go to Swansea University as part of a PhD dissertation. It is possible that in future they could also be published in a scientific medical journal. Any quotes used from interviews in any report or publication will not contain information that will enable you to be identified.

WHO HAS REVIEWED THE STUDY?
All research in the NHS is looked at by independent group of people, called a Research Ethics Committee, to protect your interests. This study has been reviewed and given favourable opinion by the Cardiff and Vale Research Review Service and the South East Wales Research Ethics Committee.

WHO IS ORGANISING THIS STUDY?
Aled Williams, a Chartered Physiotherapist working at the University Hospital of Wales as the Clinical Lead in Musculo-skeletal physiotherapy. This study forms part of the PhD at Swansea University which Aled is working towards over the next few years.

CONTACT FOR FURTHER INFORMATION
If you have any further questions about this study please contact Aled Williams on 02920 742625.

Thank you for taking the time to read this information sheet and I look forward to your response in the near future.

Aled Williams, Chartered Physiotherapist 22/02/12

Centre Number:

Study Number:

Participant Identification Number for this trial:

PARTICIPANT CONSENT FORM

Title of Project: *The Effect of Training Physiotherapists in Clinical Reasoning on Patient Outcome*

Name of Researcher: Aled Williams

Please initial box

1. I confirm that I have read and understand the information sheet dated.. (version............) for the above study. I have had the opportunity to consider the information, ask questions and have had these answered satisfactorily.

2. I understand that my participation is voluntary and that I am free to withdraw at any time without giving any reason, without my usual training or professional or legal rights being affected.

3. I agree to take part in the above study.

Name of Participant	Date	Signature
Name of Person taking consent	Date	Signature

When completed: 1 for participant; 1 for researcher site file.

Appendix D: Criteria for assessment of therapist performance

THE UNIVERSITY OF BIRMINGHAM
SCHOOL OF HEALTH SCIENCES
MODULE GUIDE 2009/2010 academic year

Criteria for the assessment of the Evidence Based Clinical Practice module

This format and content of this module is negotiated according to the learning needs of the individual student. It is therefore anticipated that the importance of the different components of the following criteria will vary according to these needs and the requirements of the programme that the student is registered on.

80 - 100
Satisfies all the criteria in the 70 - 79 band to an exceptional degree, representing superlative academic and practice integrative accomplishment.

80 - 100
Excellent knowledge of specialist area
Excellent clinical reasoning skills
Excellent synthesis of theoretical and practice knowledge
Comprehensive analysis and appraisal of concepts and evidence
Highly appropriate critical use of theoretical perspectives
Innovative and creative links between theory and practice
Excellent adaptability to new situations
A high level of justification of decision making
Highly developed self evaluative skills and identification of learning needs
Expertise of practical skills
Expertise of communication skills

70 - 79
Very good knowledge of specialist area
Very good clinical reasoning skills
Very good synthesis of theoretical and clinical knowledge
In depth analysis and critical appraisal of concepts and evidence
Very good, critical use of theoretical perspectives
Very good adaptability to new situations
Very good justification of decision making
Well developed self evaluative skills and identification of learning needs
Very good practical skills
Very good communication skills

60 - 69
Good knowledge of specialist area
Good clinical reasoning skills
Good synthesis of theoretical and clinical knowledge
Good analysis and appraisal of concepts and evidence
Good, critical use of theoretical perspectives
Good adaptability to new situations
Good justification of decisions making
Effective self evaluation and identification of learning needs
Good practical skills
Good communication skills

50 - 59
Effective knowledge of subject area
Effective clinical reasoning skills
Satisfactory synthesis of theoretical and clinical knowledge
Appropriate analysis and appraisal of concepts and evidence
Possibly some difficulty in handling more than one perspective
Ability to evaluate strengths and weaknesses of a theory
Effective adaptability to new situations
Effective justification of decision making
Ability to self evaluate and identify learning needs
Effective and efficient practical skills but require developing
Effective communication skills

Fail

40 - 49
Adequate knowledge of subject area
Adequate clinical reasoning skills but require development to improve effectiveness
Some difficulty in synthesizing theoretical and clinical knowledge
Limited analysis and appraisal of concepts and evidence
Little use made of theoretical perspectives
Difficulty in evaluating the strengths and weaknesses of a theory
Some adaptability to new situations
Limited justification of decisions making
Some ability to self evaluate and identify learning needs but this can be developed considerably
Competent practical skills but not always efficient and effective
Appropriate communication skills but require development to be effective

Fails to meet standards required of the criteria in the 50 - 59 band. Safety to practice may be an issue.

Below 40

Fails to meet the standards required of the criteria in the 40 - 49 band. Safety to practice may be an issue.

Appendix E: Chart and Calculations for Power Calculations

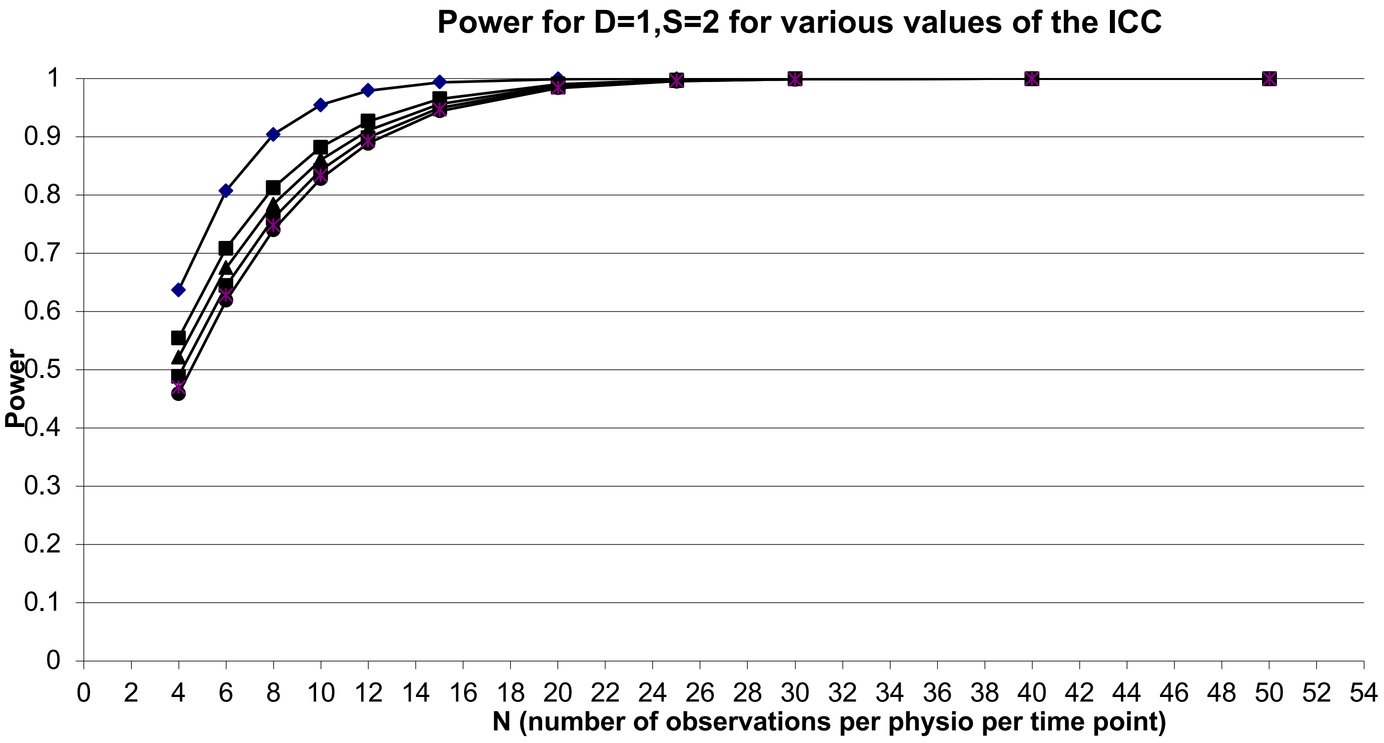

N	9			tau	0	0.22	0.44	1	2	4
sig-e^2	4									
tau^2	1			icc	0	0.05	0.1	0.2	0.33	0.5
			N	4	0.636629	0.554501	0.521758	0.488968	0.470428	0.459334
icc	0.2			6	0.80743	0.708378	0.674865	0.644305	0.628224	0.618982
sig^2	0.444444			8	0.904228	0.812968	0.78467	0.760126	0.747678	0.740666
				10	0.954631	0.88215	0.860583	0.842425	0.833412	0.828393
T	4			12	0.979327	0.926889	0.911481	0.898751	0.892519	0.889074
I	12			15	0.994	0.965175	0.956596	0.949627	0.94626	0.94441
				20	0.999322	0.990444	0.987636	0.985387	0.984312	0.983726
U	24			25	0.999932	0.997526	0.996707	0.996055	0.995745	0.995577
W	224			30	0.999994	0.999389	0.999168	0.998993	0.99891	0.998865
V	56			40	1	0.999967	0.999953	0.999942	0.999937	0.999934
				50	1	0.999998	0.999998	0.999997	0.999997	0.999997
top1	5.333333									
top2	4.444444									
bot1	28.44444									
bot2	160									
variance	0.125786									
sd	0.354663									
diff	1									
delta	2.819574									
alpha	0.05									
z	1.959964									
arg1	-0.85961									
arg2	-4.77954									
power	0.804999									

Appendix F

Monthly Training Requirements Form

Period covered: From............................ To:............................

Costs of Attendance (Mentor / Trainee):				Costs of Delivery (Mentor / Trainer):					
Training Type: (IST / mentoring / techniques)	Down time from diary (non-patient contact time):	Travel to venue / parking:	Cost: (research use only)	Teaching Time allocated:	Delivered by (Band):	Number of others receiving training:	Resources used (e.g. laptop, projector):	Travel to venue / parking:	Cost: (research use only)
